# Ionothermal Synthesis of Imide‐Linked Covalent Organic Frameworks

**DOI:** 10.1002/anie.202007372

**Published:** 2020-08-11

**Authors:** Johannes Maschita, Tanmay Banerjee, Gökcen Savasci, Frederik Haase, Christian Ochsenfeld, Bettina V. Lotsch

**Affiliations:** ^1^ Nanochemistry Department Max Planck Institute for Solid State Research Heisenbergstraße 1 70569 Stuttgart Germany; ^2^ Department of Chemistry University of Munich (LMU) Butenandtstraße 5–13 81377 München Germany; ^3^ E-conversion and Center for Nanoscience Lichtenbergstraße 4a 85748 Garching bei München Germany; ^4^ Current address: Institute for Functional Interfaces Karlsruhe Institute of Technology (KIT) Hermann-von-Helmholtz-Platz 1 76344 Eggenstein-Leopoldshafen Germany

**Keywords:** covalent organic frameworks, eutectic salt mixtures, ionothermal synthesis, polyimides

## Abstract

Covalent organic frameworks (COFs) are an extensively studied class of porous materials, which distinguish themselves from other porous polymers in their crystallinity and high degree of modularity, enabling a wide range of applications. COFs are most commonly synthesized solvothermally, which is often a time‐consuming process and restricted to well‐soluble precursor molecules. Synthesis of polyimide‐linked COFs (PI‐COFs) is further complicated by the poor reversibility of the ring‐closing reaction under solvothermal conditions. Herein, we report the ionothermal synthesis of crystalline and porous PI‐COFs in zinc chloride and eutectic salt mixtures. This synthesis does not require soluble precursors and the reaction time is significantly reduced as compared to standard solvothermal synthesis methods. In addition to applying the synthesis to previously reported imide COFs, a new perylene‐based COF was also synthesized, which could not be obtained by the classical solvothermal route. In situ high‐temperature XRPD analysis hints to the formation of precursor–salt adducts as crystalline intermediates, which then react with each other to form the COF.

## Graphical abstract

Aiming for synthetic diversity and simplification of the synthesis, a generic ionothermal approach for imide‐linked covalent organic frameworks has been developed. By using ZnCl_2_ and eutectic salt mixtures, highly crystalline COFs were synthesized with a significantly reduced reaction time of 10 hours. Unlike common solvothermal methods this approach does not require soluble precursor molecules.

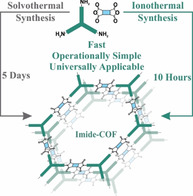

## Introduction

Covalent organic frameworks (COFs) are crystalline, porous organic materials composed of light, earth‐abundant elements that are constructed from molecular building blocks. 2D‐COFs form extended planar networks via in‐plane covalent bonding and are further stacked in the third dimension by virtue of out‐of‐plane π‐π interactions and van‐der‐Waals forces.^1^ Owing to their modularity and tunability, over the past years, COFs have found applications in various fields including gas separation and storage,^2^ catalysis,^3^ and optoelectronics.^4^ COFs are synthesized following the principles of dynamic covalent chemistry,^1^, ^5^ according to which the COF formation reaction has to be reversible to enable crystal defect correction under thermodynamic control. Bond breaking is thus as crucial as bond formation: Stability is achieved at the expense of crystallinity, which frequently leads to poorly crystalline products, rendering COF synthesis of stable and crystalline COFs challenging. COFs are synthesized in a variety of ways, including solvothermal synthesis,^1^ microwave‐assisted synthesis,^6^ mechanochemical synthesis,^7^ vapor‐assisted conversion^8^ etc. Nevertheless, solvothermal synthesis is the most prominent and frequently used method in COF synthetic chemistry. Although countless COFs have been synthesized using this approach, this method typically requires long reaction times ranging from 3–7 days and also requires precursor molecules with an appreciable solubility. In particular, the complexity of the crystallization and aggregation processes strongly affects the crystallinity and porosity of COF systems.^9^ In order to obtain satisfactory products, unique synthesis conditions are often required, resulting in re‐optimization of reaction conditions with each new COF. This leads to time‐consuming and empirical rather than rational solvent screening. The challenge associated with the synthesis of highly crystalline, porous materials has partially motivated the quest for new COF linkages such as ester borosilicate,^10^ imine,^11^ hydrazone,^12^ borazine.^13^ However, in order to improve the stability and crystallinity of the products, further optimization of the thermodynamics and kinetics of the condensation processes by exploring new synthesis routes and linkage chemistry is quintessential. In this regard the imide‐linkage introduced by Yan and co‐workers is of particular interest.^14^ The imide formation reaction is used to produce commercially relevant one‐dimensional imide polymers—especially aromatic polyimides—which are high‐performance polymers known for their good chemical resistance, high thermal stability, and extraordinary mechanical properties.^15^ Adorned with many of these properties, polyimide‐linked COFs (PI‐COFs) have been used for sensing,^16^ decontamination^17^ and energy storage applications.^18^ In polyimide condensation reactions, however, the formation of the cyclic imide is essentially irreversible under solvothermal conditions, which makes the formation of the thermodynamic and crystalline product challenging.^19^ To overcome this obstacle, the classical synthesis of polyimides involves harsh conditions, namely, usage of high‐boiling and toxic solvents, toxic catalysts, high processing temperatures, and long reaction times of up to seven days.^14^, ^16^, ^17^, ^18^, ^20^

Research on carbon nitrides has demonstrated the pertinence of ionothermal synthesis approaches using salt melts both as high‐temperature solvent and structure directing agent. While the 1D polymer melon is obtained by classical solid state reactions, eutectic mixtures of alkali metal chlorides furnish the 2D polymers poly(triazine imide), PTI, or poly(heptazine imide), PHI.^21^ Likewise, covalent triazine frameworks (CTFs) have been synthesized from ZnCl_2_ salt melts, which act as reactive high temperature solvent and catalyst.2a Following these observations, we present an ionothermal synthesis of porous, crystalline imide‐linked COFs using an inorganic salt, namely ZnCl_2_, as reaction medium. This new method allows us to avoid the usage of environmentally harmful solvents and catalysts in large quantities and to reduce the reaction time significantly compared to the conventional solvothermal methods. As compared to CTFs, the low temperature ionothermal synthesis further prevents the formation of carbonaceous products, while still leading to complete COF formation. We then optimized our reaction protocol to further increase the substrate scope and report the synthesis of porous, crystalline imide‐linked COFs in eutectic salt mixtures for the first time.

## Results and Discussion

**Scheme 1:**
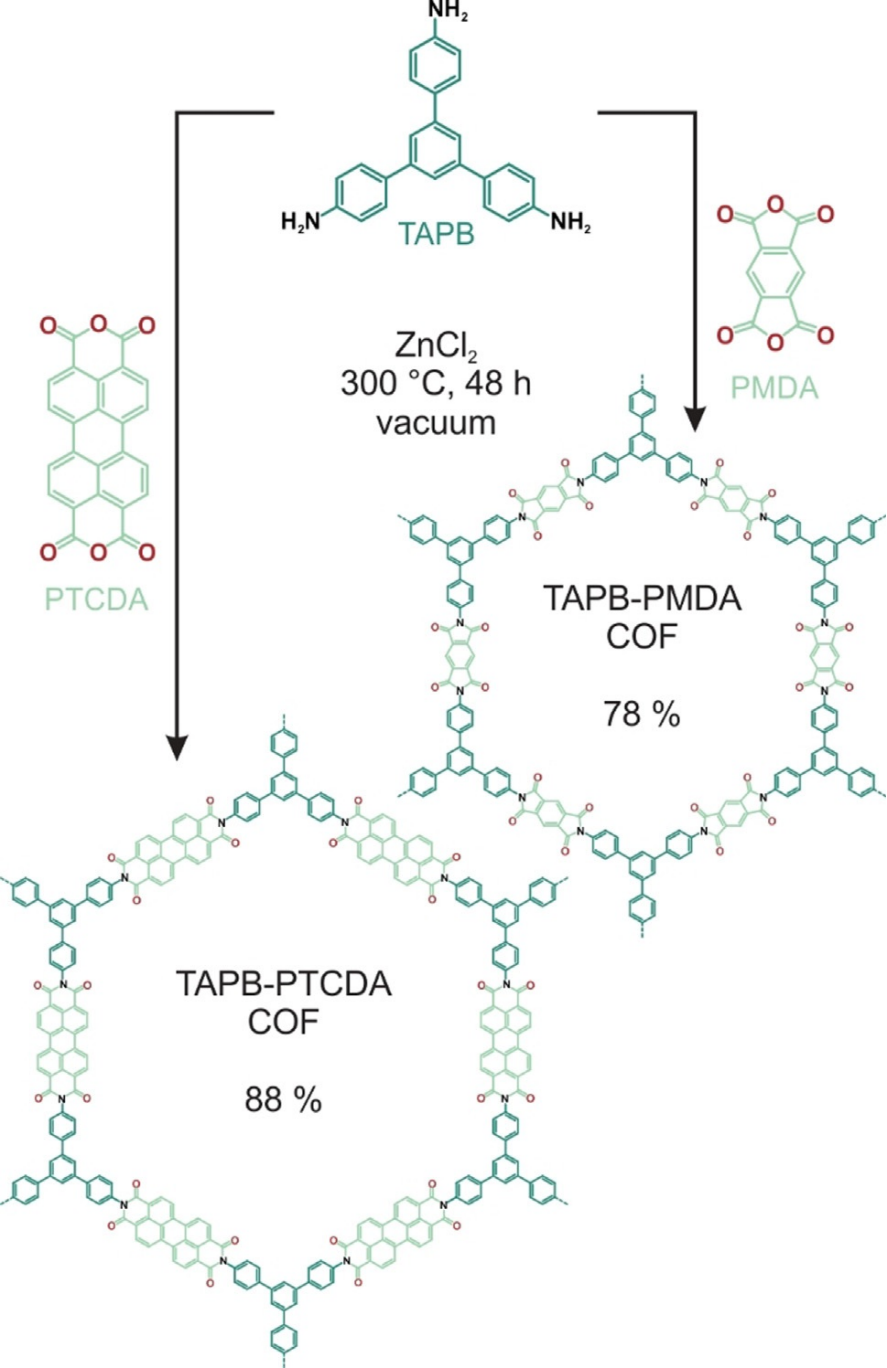
Synthesis of TAPB‐PTCDA COF and TAPB‐PMDA COF in ZnCl_2_ under ionothermal conditions.

In the first step of this study, two PI‐COFs, namely TAPB‐PMDA‐ and TAPB‐PTCDA‐COF, were synthesized using the trigonal linker molecule 2,4,6‐tris(4‐aminophenyl)‐benzene (TAPB) and the two linear linker molecules pyromellitic dianhydride (PMDA) and perylene‐3,4,9,10‐tetracarboxylic dianhydride (PTCDA), respectively (Scheme 1). While TAPB‐PMDA‐COF has been reported before,^14^ this is the first report of the synthesis of the TAPB‐PTCDA‐COF, which is not accessible using previously reported reaction conditions.

**Figure 1:**
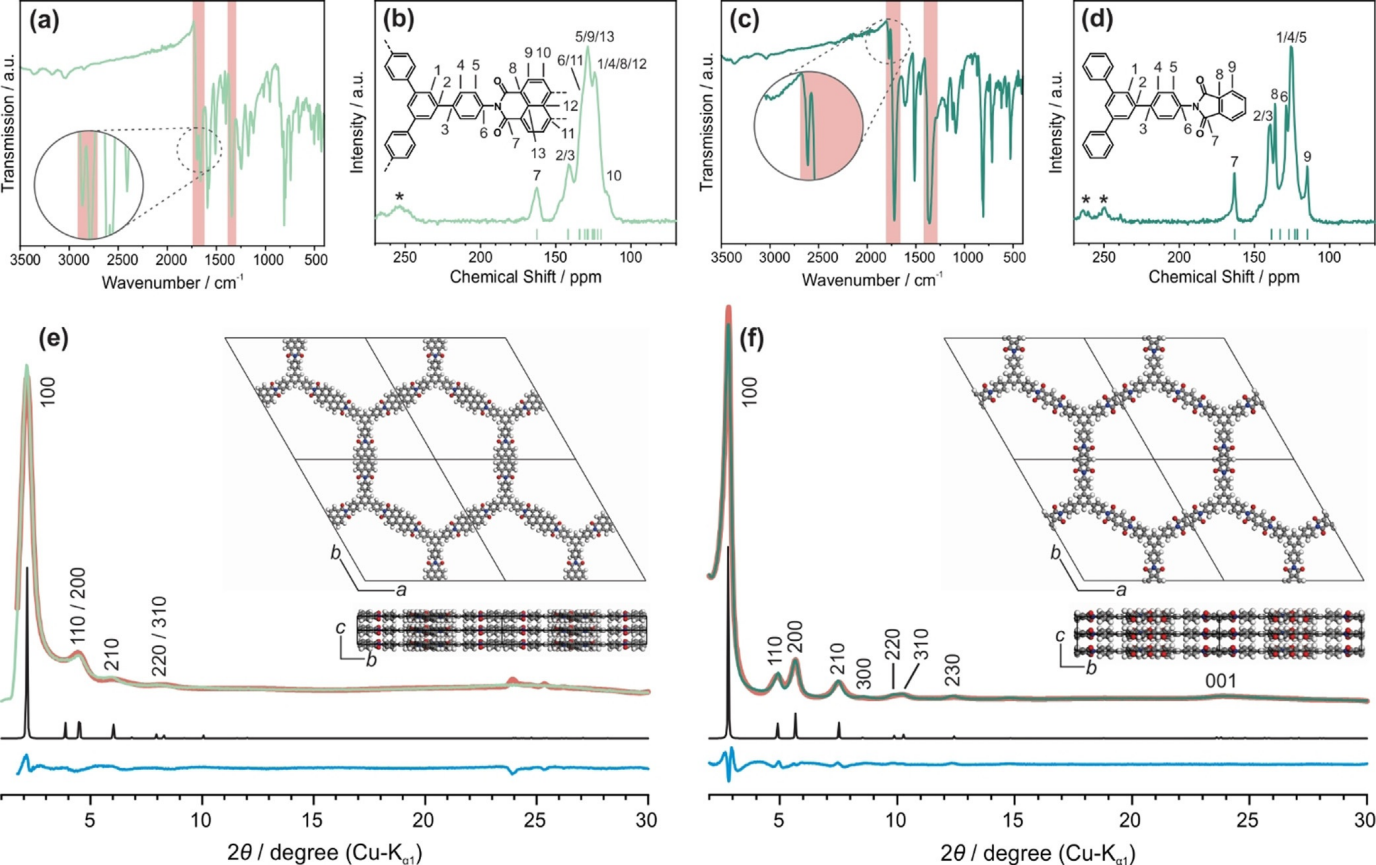
Characterization of TAPB‐PTCDA‐COF (light green) and TAPB‐PMDA‐COF (dark green): a,c) IR spectra showing the imide vibrational bands indicating quantitative imide formation. b,d) ^13^C ssNMR spectra showing the chemical shifts of the imide ring carbons between 162 and 164 ppm. Spinning side bands are marked with asterisks. Green dashes indicate calculated NMR chemical shifts for TAPB‐PTCDA‐COF and TAPB‐PMDA‐COF. Calculations were performed on the B97‐2/pcsSeg‐2 level of theory^23^ using the FermiONs++23c, 23d software package. e,f) Experimental XRPD pattern of the respective COF (green) together with the Rietveld fit (red), simulated patterns based on space group $P\bar{3}1m$ (black), and difference curve (blue). Inset: Simulated structures of the respective COF along the *a* and *c* axis.

In a typical reaction procedure, the precursor molecules—the linear dianhydride and the trigonal amine—were mixed together with anhydrous ZnCl_2_ and heated to 300 °C in a flame sealed glass tube under vacuum for 48 h to give crystalline solids at yields of 88 % for TAPB‐PTCDA‐COF, and 78 % for TAPB‐PMDA‐COF (Scheme 1). The imide formation reaction was confirmed by Fourier transform infrared spectroscopy (FT‐IR) as depicted in Figure 1 a and c. The spectra show signals at 1696 and 1662 cm^−1^ for TAPB‐PTCDA‐COF, and at 1776 and 1721 cm^−1^ for TAPB‐PMDA‐COF, corresponding to the asymmetric and symmetric C=O stretching vibrations of the six‐ and five‐ membered imide rings, respectively. Additional signals at 1344 and 1371 cm^−1^ are observed, corresponding to the C‐N‐C stretching vibration of the imide rings. Together with the absence of anhydride C=O stretching and amine N‐H stretching vibrations corresponding to the respective starting materials (see Figures S1 and S2), this proves the successful and complete reaction between TAPB and PTCDA/PMDA to form the respective COFs. The formation of the imide ring was further confirmed by solid‐state ^13^C cross‐polarization magic‐angle‐spinning nuclear magnetic resonance (CP‐MAS ssNMR) spectroscopy with the spectra showing the carbonyl carbon of the imide ring at 162.5 ppm for TAPB‐PTCDA‐COF, and at 163.3 ppm for TAPB‐PMDA‐COF (Figure 1 b, d).^14^ Quantum‐chemical calculations of the expected NMR shifts (Figure 1 b, d and S3–S8) further support this assignment. X‐ray powder diffraction (XRPD) shows that both TAPB‐PTCDA‐COF and TAPB‐PMDA‐COF are crystalline. The XRPD pattern of TAPB‐PTCDA‐COF (Figure 1 e and S9) shows reflections at 2.14°, 4.44°, 5.92° and 8.09° 2*θ* corresponding to the 100, 200, 210 and 220/ 310 Bragg peaks of a trigonal lattice with $P\bar{3}1m$ symmetry. Structure simulation and geometry optimization was carried out by applying the universal force field method with the Materials Studio software package.^22^ Using this simulated model, the experimental powder pattern was then Rietveld refined yielding the unit cell parameters of *a*=*b*=43.6(2) Å and *c*=3.71 Å with *α*=*β*=90° and *γ*=120° (R_wp_=3.92).

The XRPD pattern of TAPB‐PMDA‐COF shows reflections at 2.77°, 4.90°, 5.64°, 7.48°, 8.55°, 9.88°, 10.20°, 12.35° and 23.92° 2*θ* corresponding to the 100, 110, 200, 210, 300, 220, 310, 230 and 001 Bragg peaks, again indexed in a trigonal lattice with $P\bar{3}1m$ symmetry (Figure 1 f and S10). Rietveld refinement yielded the unit cell parameters *a*=*b*=35.7(4) Å and *c*=3.76(5) Å with *α*=*β*=90° and *γ*=120° (R_wp_=4.98). We also calculated models with AB stacked $P\bar{3}1c$ symmetry, but we found that the eclipsed model fits the experimental data for both COFs best (Figure S9 and S10). Following a previous report^14^ we also took a model with *Cmcm* symmetry into account, which matches the experimental data as well. However, DFT calculations indicate a non‐planar geometry of the COF layers with the benzene units and the anhydride building blocks twisted against each other, leading to a structure with the higher $P\bar{3}1m$ symmetry (Figures S3–S8). In the DFT calculations atom positions and lattices of all periodic structures were optimized on the RI‐PBE‐D3/def2‐TZVP^24^ level of theory using an acceleration scheme based on the resolution of the identity (RI) technique and the continuous fast multipole method (CFMM^25^) implemented^26^ in Turbomole^27^ version V7.3.

**Figure 2:**
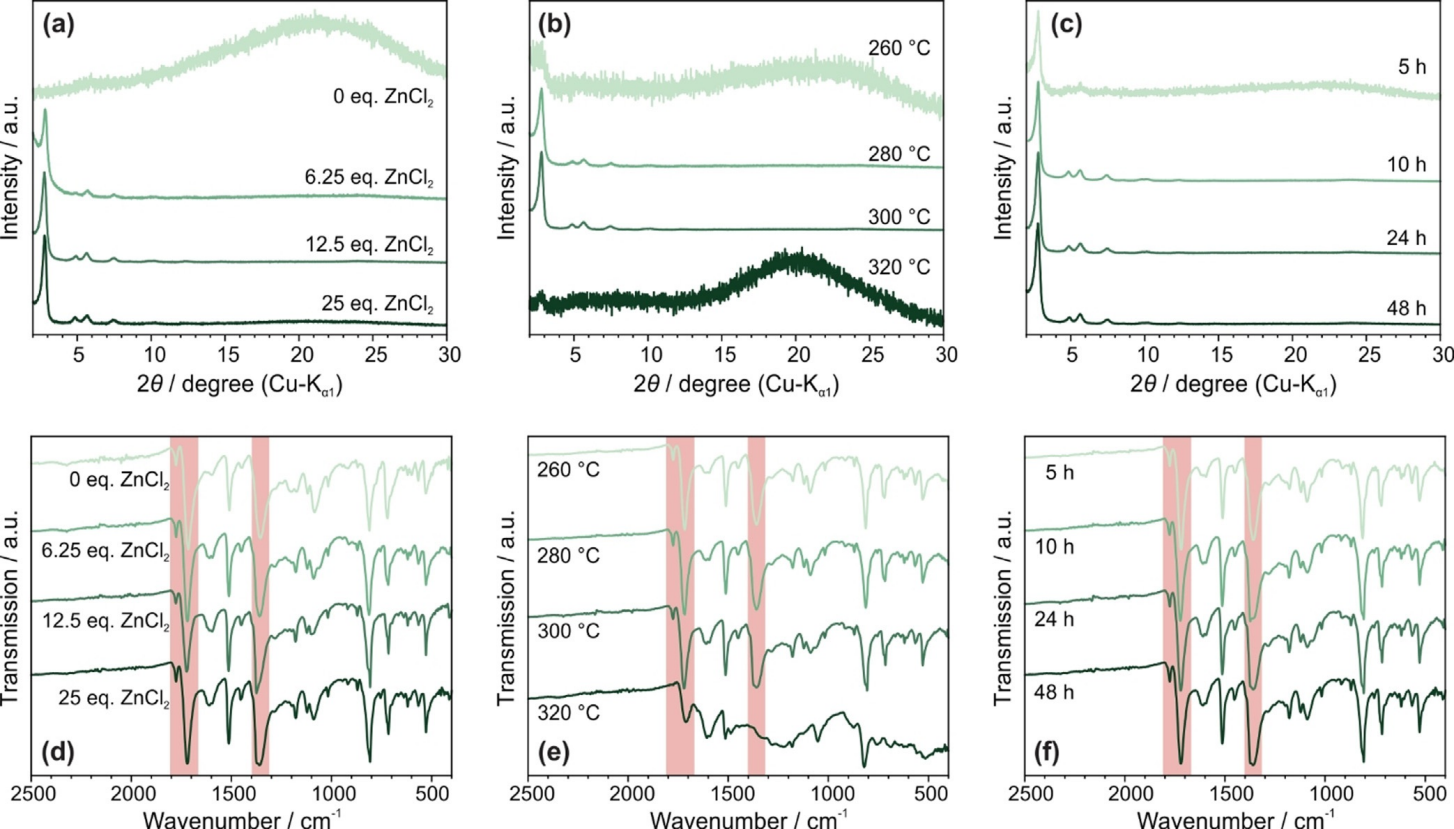
XRPD patterns (a–c) and IR spectra (d–f) of ex situ reaction condition optimization experiments with TAPB‐PMDA‐COF. a) and d) show the XRPD patterns and IR spectra of experiments in which the ZnCl_2_ ratio was varied between 0 and 25 equivalents with respect to PMDA. The reaction time and temperature were kept constant at 48 h and 300 °C, respectively. b) and e) show the XRPD patterns and IR spectra of experiments in which the reaction temperature was varied between 260 and 320 °C, with constant ZnCl_2_ to precursor ratio (12.5 equiv.) and reaction time (48 h). c) and f) show the XRPD pattern and the IR spectra of experiments in which the reaction time was varied between 5 and 48 h with constant ZnCl_2_ concentration (12.5 equiv.) and reaction temperature (300 °C).

Argon sorption measurements at 87 K (Figures S12) were performed to determine the porosity of the samples. Both TAPB‐PTCDA‐COF and TAPB‐PMDA‐COF show type‐IV behavior and steep rise in the gas uptake below 0.4 *p*/*p*_0_. Pore size distributions (PSD) were calculated using the quenched solid‐state functional theory (QSDFT) method, which reveals pores of 31 Å width for TAPB‐PTCDA‐COF, and 29 Å for TAPB‐PMDA‐COF. For TAPB‐PMDA‐COF these results are in good agreement with the pore size extracted from transmission electron microscopy (TEM) (30 Å) (Figure S13) and with the calculated pore size of 31 Å. For TAPB‐PTCDA‐COF, the pore size obtained by PSD calculations does not match the pore sizes extracted from TEM (36 Å) (Figure S14) or theoretical calculations (37 Å). The Brunauer‐Emmett‐Teller (BET) model revealed surface areas of 460 m^2^ g^−1^ for TAPB‐PTCDA‐COF and 1250 m^2^ g^−1^ for TAPB‐PMDA‐COF. The relatively small surface area of TAPB‐PTCDA‐COF together with the unexpectedly small pore size obtained from sorption measurements is an indication of possible pore blocking through insoluble precursor molecules (e.g. PTCDA) or oligomers; it may also indicate random AA′ slip stacking contributions which effectively decrease the measured pore size. For TAPB‐PMDA‐COF, the BET surface area is similar the previously reported surface area of 1297 m^2^ g^−1^ that employs the classical solvothermal synthesis.^14^ To understand the parameters of the ionothermal synthesis protocol that influence the crystallinity, the relative amount of ZnCl_2_, the reaction time and temperature on the PI‐COF synthesis method were investigated by testing different reaction conditions using TAPB‐PMDA‐COF. This COF was chosen for our studies because of its higher crystallinity and porosity compared to TAPB‐PTCDA‐COF. The quality of the products obtained were compared based on XRPD and FT‐IR spectroscopy. While varying the equivalents of ZnCl_2_, the reaction temperature was kept constant at 300 °C and the reaction was carried out for 48 h. With progressive increase in the equivalents of ZnCl_2_ with respect to PMDA, the crystallinity of the final product was observed to increase up to 12.5 equivalent amounts. Using more ZnCl_2_ did not increase the crystallinity further (Figure 2 a). As ZnCl_2_ starts to melt slightly above 300 °C (Figure S15), at the reaction temperature of 300 °C, we did not observe the formation of a melt (Figures S15 and S16). Thus, the increase in crystallinity is likely a blend of several factors, including thorough mixing of the reactants and increased exposure of the precursors to reactive zinc and chloride ions, which become increasingly mobile close to the melting point. This complete exposure is possibly reached at 12.5 equiv. ZnCl_2_ and further increase shows no effect. However, the fact that no melting is observed in our case stands in contrast to CTF syntheses where the nitrile precursor molecules get dissolved in the ZnCl_2_ melt, forming a clear solution.2a Also, ionothermal synthesis enables highly crystalline COF formation from reactants sparingly soluble in typical organic solvents, such as PTCDA.

It is also interesting to note that even when no ZnCl_2_ is used, the IR spectrum of the final product shows the typical imide vibrations (asymmetric and symmetric C=O stretching vibration at 1776 and 1721 cm^−1^, and C‐N‐C stretching vibration at 1371 cm^−1^) (Figure 2 d). However, no crystalline product is formed in the absence of the salt. The fact that the imide condensation reaction takes place even when no ZnCl_2_ is used highlights the important role of ZnCl_2_ in imparting reversibility to the imide formation reaction, and thereby crystallinity to the final product.

As mentioned previously, the commonly used method for synthesizing PI‐COFs is solvothermal synthesis using high boiling solvents such as mesitylene, N‐methyl‐2‐pyrrolidone (NMP), or dimethylformamide (DMF) at temperatures between 160 °C and 250 °C for three to seven days. To impart reversibility to the formation of the very stable imide ring, a base (usually Isoquinoline) is added as a catalyst.^14^, ^17^, ^18^, ^20^, ^28^ On the other hand, with our newly developed synthesis route, only ZnCl_2_ is needed for the PI‐COF synthesis, completely circumventing the need for any additional solvent or catalysts.

The reaction temperature was then varied (48 h, 12.5 equiv. ZnCl_2_) and crystalline product was found to form only between 280 and 300 °C (Figure 2 b). The fact that no crystalline product formed below 280 °C shows that not only the presence of ZnCl_2_ is necessary but an increased ion mobility of the zinc and chloride ions induced by the elevated temperature is required. At temperatures above 300 °C, the system begins to lose its short‐ and long‐range ordering, possibly owing to the harsh (i.e. high temperature and Lewis acidic) conditions induced by the complete melting of ZnCl_2_. This decomposition of the polymer is indicated by the analysis of the IR spectrum wherein a significant broadening of the vibrational bands is observed when the reaction is carried out at 320 °C, vs. the reaction carried out at 300 °C (Figure 2 e). Nevertheless, the possibility of achieving higher reaction temperatures compared to solvothermal synthesis is beneficial in terms of overcoming the activation energy of the imide ring closing/opening reaction, resulting in a significantly decreased reaction time. It is also important to note that, in contrast to the ionothermal synthesis of CTFs where a high reaction temperature (≈400 °C) leads to partial carbonization^29^ and a lower reaction temperature leads predominantly to oligomers,^30^ the lower reaction temperatures in the ionothermal synthesis of imide‐linked COFs reported herein leads to porous and highly crystalline PI‐COFs.

Keeping the [ZnCl_2_] to [precursor] ratio fixed at 12.5 and the reaction temperature constant at 300 °C, the reaction time was varied. Crystalline product was detected already after five hours of reaction and beyond ten hours, the crystallinity remained nearly unchanged (Figure 2 c,f). This shows a significant advantage of this new method over previously reported solvothermal methods, where three to seven days are usually required to form crystalline products.^14^, ^16^, ^17^, ^18^, ^20^ The faster ionothermal reaction can be rationalized on the basis of a two‐fold effect of ZnCl_2_: First, due to the Lewis acidic properties, ZnCl_2_ activates the carbonyl functions of the anhydride and the imide, rendering them more reactive. Second, access to higher reaction temperature facilitates the overcoming of the activation barrier for the condensation reaction in both directions, leading to higher reversibility.

**Figure 3:**
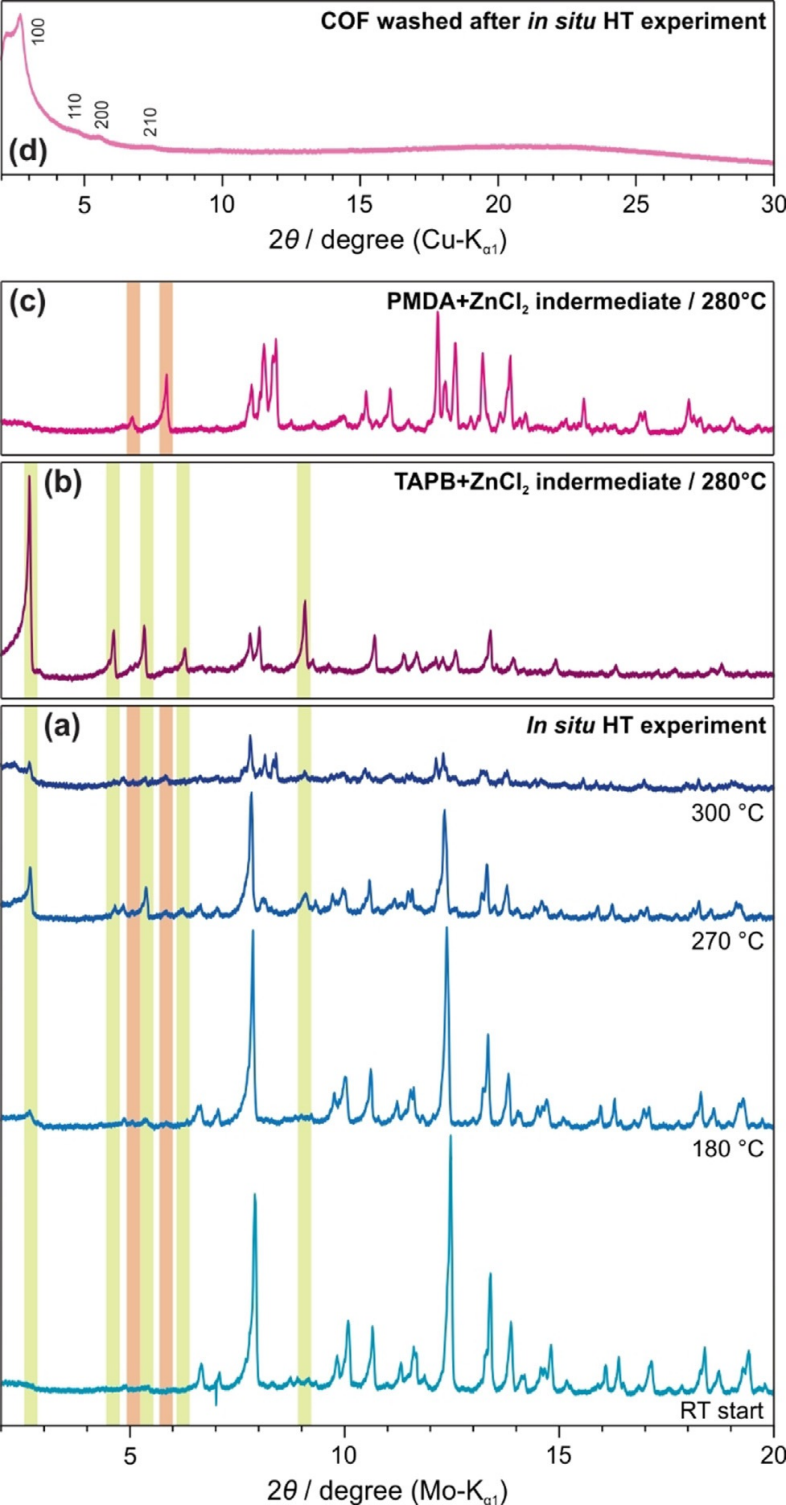
In situ HT‐XRPD (Mo‐K_α1_) experiments of a) the TAPB‐PMDA‐COF reaction mixture, b) a mixture of TAPB and ZnCl_2_, and c) a mixture of PMDA and ZnCl_2_. d) The XRPD pattern (Cu‐K_α1_) of the resulting TAPB‐PMDA‐COF from (a). Reflections corresponding to the TAPB‐ZnCl_2_ adduct are marked green while those corresponding to the PMDA‐ZnCl_2_ adduct are marked orange.

To get further insights into the COF formation process and to clarify the role of ZnCl_2_ in this method, in situ high temperature XRPD (HT‐XRPD) measurements of TAPB‐PMDA‐COF were carried out in a quartz capillary. In this HT‐XRPD measurement, the temperature was raised from room temperature to 300 °C and cooled down again with a dwelling period of 10 h at 300 °C (Figure 3 a, see Figure S17 for full data). Significant changes in the XRPD pattern of the reaction mixture were observed with increasing temperature. The reflections corresponding to the resulting COF are not visible, likely due to its lower scattering contrast and poorer crystallinity as compared to the other (inorganic) species present, and due to significant pore blocking during the reaction, further reducing the scattering contrast. However, after isolating and washing the COF, the corresponding reflections can be observed (Figure 3 d). To be able to identify the appearing side phases, all precursor molecules (TAPB, PMDA and ZnCl_2_) and all possible combinations of the precursors were also investigated via HT‐XRPD (Figures S18–S23). In the HT‐XRPD experiment of the COF formation, the reflections corresponding to ZnCl_2_ dominate the pattern at room temperature and the low intensity reflections of the precursor molecules are not visible. With increasing temperature, a phase transition of ZnCl_2_ is observed—above 240 °C, the monoclinic γ‐form undergoes a phase transition to the orthorhombic δ‐form. Additional reflections at 2.67°, 4.63°, 5.05°, 5.38°, 5.86°, 6.34° and 9.13° 2*θ* were observed in the HT‐XRPD patterns at 180 °C and higher (see pattern at 270 °C). The intensities of these appearing reflections decrease significantly at the final reaction temperature of 300 °C (Figure 3 a), indicating that these transient reflections correspond to reaction intermediates, which then react to form the COF. Crystallite sizes and the relative proportions of the crystalline phases calculated using Scherrer analysis support the formation of reaction intermediates (Figure S24). A gradual increase of the crystallite size and relative amounts of the intermediate phases with a maximum at the optimized reaction temperature between 280 and 300 °C can be observed. This was further confirmed by recording the HT‐XRPD pattern of individual mixtures of PMDA and TAPB with ZnCl_2_. For the mixture of PMDA and ZnCl_2_ at 280 °C, the formation of a new species was evident with reflections at 5.05° and 5.86° 2*θ*, observed neither in the diffraction pattern of pure PMDA nor in the pattern of pure ZnCl_2_ (Figures 3 c and S22). These reflections however correspond exactly to two of the additional reflections from the HT‐XRPD pattern of the COF reaction mixture at 180 °C and higher, suggesting the species to be a reaction intermediate (Figure 3 a, c—marked orange) in the imide‐linked COF formation reaction. FT‐IR spectroscopy of the intermediate revealed significant changes of the C‐O related vibrational bands when compared to the precursor molecule, hinting towards the possible formation of a PMDA‐ZnCl_2_‐adduct (Figure S25). Again, for the mixture of TAPB and ZnCl_2_ at 280 °C, the formation of a highly crystalline species is evident with reflections at 2.67°, 4.63°, 5.38°, 6.34° and 9.13° 2*θ* (Figures 3 b and S23). These reflections correspond to the reflections from the HT‐XRPD pattern at 270 °C of the COF reaction mixture as well, again suggesting this species to be a reaction intermediate (Figure 3 a, b—marked green) in the COF formation reaction. FT‐IR spectroscopy of this intermediate shows a significant shift of all amine group related vibrational bands when compared to the precursor molecule, also hinting towards the formation of an adduct consisting of TAPB and ZnCl_2_ (Figure S26). It is important to note that the formation of these reaction intermediates occurs at temperatures lower than the melting points of the individual precursor molecules and ZnCl_2_ (Figures S18–S20). In order to confirm whether the aforementioned adducts generated from mixtures of TAPB/ZnCl_2_ and PMDA/ZnCl_2_ are true intermediates of the COF formation reaction or correspond to side‐reactions, we synthesized the COF in two steps. In the first step, the two air sensitive intermediate species from TAPB and PMDA were synthesized separately with a slight excess of ZnCl_2_ to ensure their complete formation. In the second step, these intermediates were combined and heated at 290 °C for 20 h, that is, under standard imide‐COF synthesis conditions, resulting in the formation of the crystalline TAPB‐PMDA‐COF (Figure S27). As crystalline COFs form already at 280 °C (Figure 2 b, e) and as the intermediates are stable in this temperature regime (Figure 3 b, c), it is thus evident that the formed adducts from reactions of the individual precursors with ZnCl_2_ are indeed true intermediates of the ionothermal COF reaction. It must also be noted that reaction of a single precursor‐ZnCl_2_ adduct with the other precursor also leads to COF formation. While this might suggest that one intermediate is dominant for COF formation, it must also be kept in mind that in situ formation of the ZnCl_2_ adduct of the other precursor is possible via partial ZnCl_2_ exchange. Quantum‐chemical calculations were carried out in order to gain insights into the underlying possible mechanism (Figures S28–S31). Calculations on phthalic anhydride and aniline as model compounds indicate that adduct formation of the precursors with ZnCl_2_ is exothermic in each case (Reaction 1, 2, Figure S28). The subsequent imide formation is again exothermic irrespective of whether the precursors react as ZnCl_2_ adducts or not (Reaction 3–6, Figure S28), indicating that all investigated reaction pathways are possibly operative. The comparison of calculated partial charges^31^ (Figure S32) between the anhydride and the anhydride adduct visualizes the polarization by ZnCl_2_. This activation of the carbonyl group then likely reduces the activation barrier during imide formation, also affecting the back reaction, hence increasing reversibility.

Apart from providing a reactive flux, the exact role of ZnCl_2_ is not clear at this point. By lowering the structural degrees of freedom with the formation of adducts, it can possibly aid formation of not only ordered intermediates, but also the formation of pre‐organized COF pores for further nucleation and thereby facilitate formation of larger crystalline domains when compared to solvothermal synthesis (Figure S33, Tables S5–S8). In that regard, it is important to note that formation of a ZnCl_2_ adduct of the product imide is thermodynamically favored as well (Reaction 7, 8, Figure S28). Thus, COF formation by heating the amorphous product, obtained by reacting the precursors only (Figure S34), in ZnCl_2_ is possibly hindered only by a lack of accessibility of ZnCl_2_ in the polymeric structure. Further investigations into understanding the structure of the adducts, the exact role of ZnCl_2_ in forming these ordered structures, and the mechanism of imide‐linked COF formation from these adducts is currently underway.

**Scheme 2:**
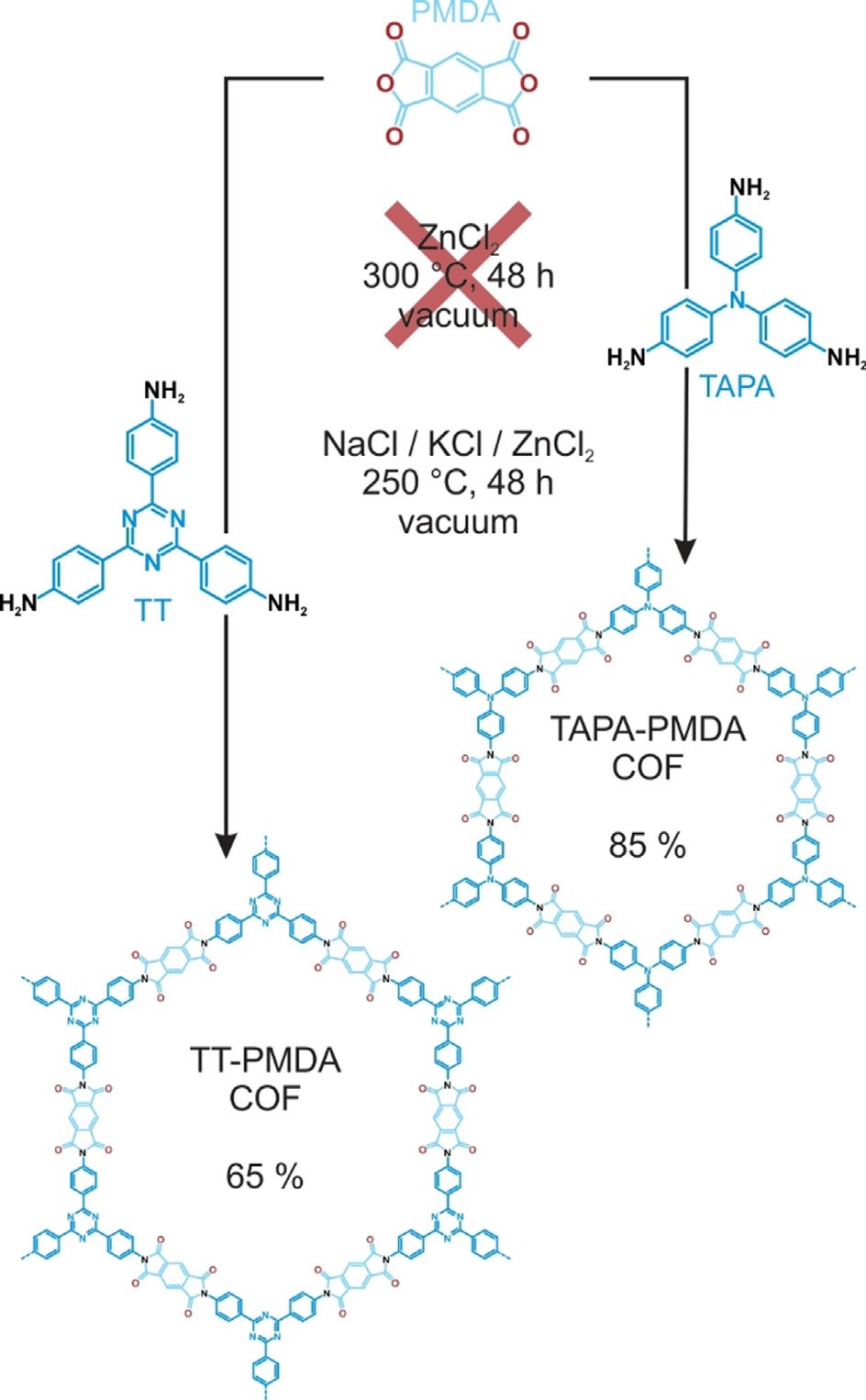
Synthesis of TT‐PMDA‐COF and TAPA‐PMDA‐COF in an eutectic salt mixture under ionothermal conditions.

While the elevated reaction temperature of 300 °C is beneficial in terms of imparting reversibility to the reaction, it could be a hindrance towards fragile linkers, which are not stable enough to withstand the strong Lewis acidic conditions at such high reaction temperatures. For example, the previously reported 2,4,6‐tris(4‐aminophenyl)‐triazine (TT) containing TT‐PMDA‐COF^17^ could not be synthesized using this method due to the decomposition of the triazine core at around 300 °C,2a which results in an ill‐defined non‐crystalline PI‐polymer/oligomer mixture instead of a well‐defined crystalline COF (Figures S35–S37). In order to avoid decomposition of fragile linker molecules such as TT and 2,4,6‐tris(4‐aminophenyl)‐amine (TAPA) (Figure S38) and to make the ionothermal synthesis universally applicable, we tested other salts with melting points below 280 °C, for example, zinc sulfate heptahydrate (mp: ≈100 °C), aluminum chloride (≈190 °C), gallium chloride (≈78 °C) and an eutectic mixture containing NaNO_3_/NaNO_2_/KNO_3_ (142 °C). No COF formation was observed in any of these reaction media, hinting possibly at the importance of ZnCl_2_ and ZnCl_2_ ‐precursor adducts observed previously in the COF formation reaction. In order to test this hypothesis, we used a ZnCl_2_ containing three‐salt eutectic mixture, namely NaCl/KCl/ZnCl_2_,^32^ with a theoretical melting point of 240 °C. In a typical reaction, the three salts (see SI for exact composition) were ground thoroughly and mixed together with the respective starting materials PMDA and TT/TAPA, followed by heating to 250 °C under vacuum (Scheme 2). Indeed, compared to the experiments with pure ZnCl_2_, this reduction in the reaction temperature not only prevented decomposition of TT and TAPA, but also TT‐PMDA‐COF and TAPA‐PMDA‐COF could be obtained in yields of 64 % and 85 %, respectively.

**Figure 4:**
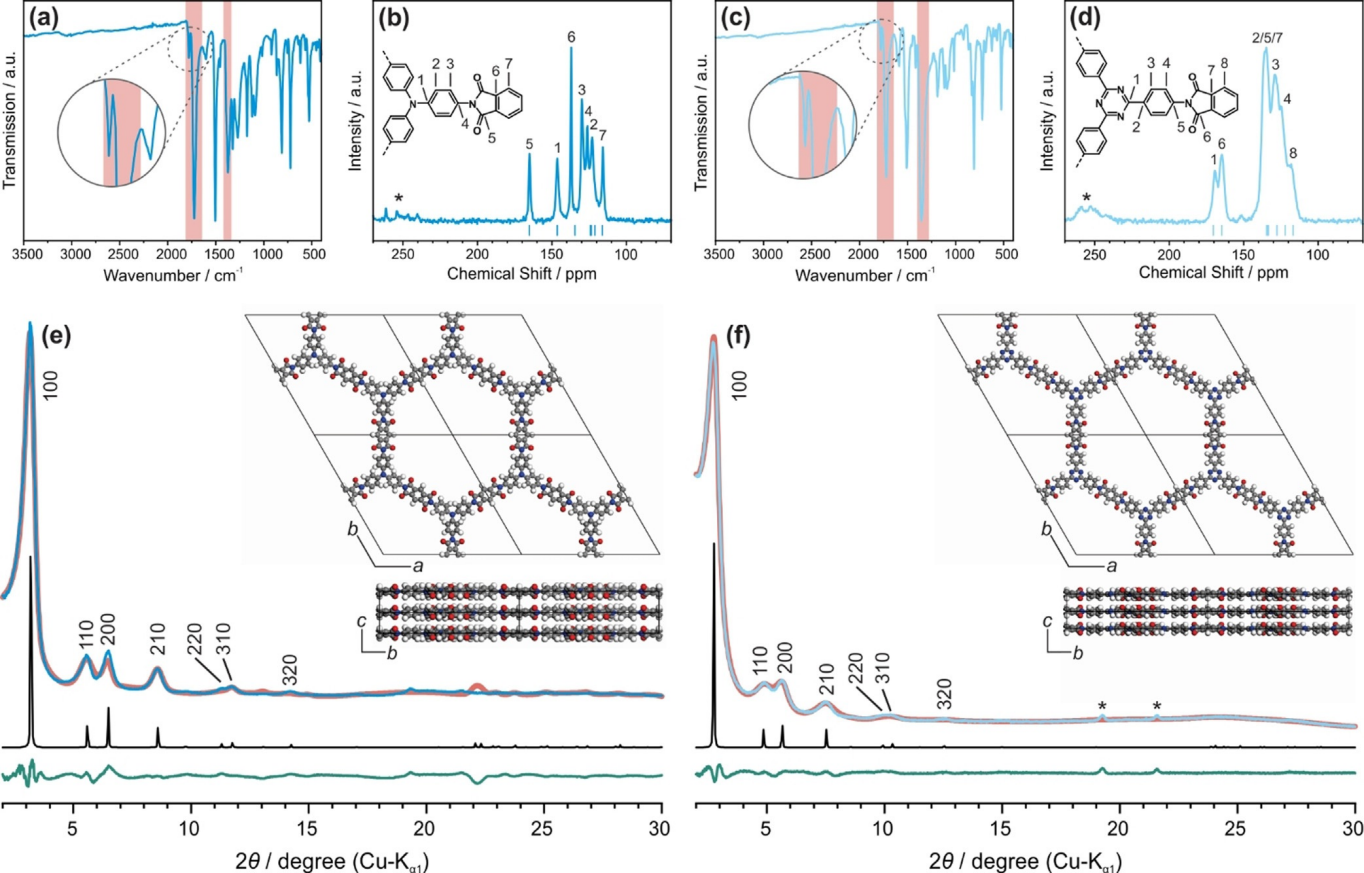
Characterization of TAPA‐PMDA‐COF (dark blue) and TT‐PMDA‐COF (light blue): a,c) IR spectra showing the imide vibrational bonds indicating full imide formation. b,d) ^13^C ssNMR spectra showing the chemical shifts of the carbonyl carbons of the imide ring around 164 ppm together with triazine carbons of the TT linker at 169 ppm and the carbons connected to the central nitrogen of the TAPA linker at 146 ppm. Spinning side bands are marked with asterisks. Blue dashes indicate calculated NMR chemical shifts for TAPA‐PMDA‐COF and TT‐PMDA‐COF. Calculations were performed on the B97‐2/pcsSeg‐2 level of theory^23^ using the FermiONs++23c, 23d software package. e,f) Experimental XRPD pattern of the respective COF (blue) together with the Rietveld fit (red), simulated patterns based on $P\bar{3}1m$ structure type (black), and difference curve (green). Insets: Simulated structures of the respective COF along the *a* and *c* axis. Reflections marked with an asterisk show impurities, which could not be eliminated.

The imide formation was again confirmed by FT‐IR spectroscopy as shown in Figure 4 a and c with C=O stretching vibrations of the imide rings at 1778 and 1723 cm^−1^ for both TT‐PMDA‐COF and TAPA‐PMDA‐COF, together with C‐N‐C stretching vibrations of the imide rings at 1357 and 1373 cm^−1^, respectively. The anhydride C=O stretching and amine N‐H stretching vibrations corresponding to the respective starting materials are again absent, which proves a complete reaction between the starting materials to form the respective COFs in the eutectic salt mixture (Figures S39 and S40). ^13^C ssNMR spectra show the carbonyl carbons of the imide ring at 164.6 ppm for TT‐PMDA‐COF (Figure 4 d), and 165.0 ppm for TAPA‐PMDA‐COF (Figure 4 b), which is consistent with the quantum‐chemical calculations (Figures 4 b, d and S41–S46).

Retention of the molecular structure of the building blocks in the framework is evident from the signal of the triazine carbon at 169.3 ppm for TT‐PMDA‐COF corresponding to the TT linker, as well as that for the carbons connected to the central nitrogen of the TAPA linker at 146.4 ppm for TAPA‐PMDA‐COF.^14^, ^17^ The XRPD pattern of TAPA‐PMDA‐COF shows reflections at 3.16°, 5.56°, 6.48°, 9.82°, 11.34°, 11.71° and 14.22° 2*θ* (Figure 4 e and S47) which can be assigned to the 100, 210, 200, 210, 220, 310 and 320 Bragg peaks of a trigonal lattice with $P\bar{3}1m$ symmetry. Using the $P\bar{3}1m$ simulated model, which is in agreement with DFT calculations (Figures S41), the experimental powder pattern was Rietveld refined yielding the unit cell parameters *a*=*b*=31.1(6) Å and *c*=4.02 Å with *α*=*β*=90° and *γ*=120° (R_wp_=9.03) with a theoretical pore size of 27 Å. The theoretical value is in good agreement with the experimentally observed pore sizes extracted from TEM (26 Å; Figure S48) and argon sorption measurements (27 Å; Figure S49). Applying the BET model revealed a surface area of 1592 m^2^ g^−1^ for our ionothermally synthesized TAPA‐PMDA‐COF, which is significantly higher when compared to that prepared solvothermally (1027 m^2^ g^−1^).^14^ For TT‐PMDA‐COF XRPD reflections at 2.73°, 4.89°, 5.65°, 7.50°, 9.97°, 10.26° and 12.50° 2*θ* are observed, which can be assigned to the 100, 110, 200, 210, 220, 310 and 320 Bragg peaks of a trigonal lattice with $P\bar{3}1m$ symmetry (Figures 4 f and S50) and the unit cell parameters *a*=*b*=35.2(5) Å and *c*=3.7(2) Å with *α*=*β*=90° and *γ*=120° (R_wp_=3.65). This is in agreement with the previously reported AA‐type stacking for this COF prepared solvothermally.^17^ PSD analysis (Figure S49) revealed pores with 30 Å diameter, which is again in good agreement with the pore channels observed in TEM (30 Å) (Figure S52) and the theoretical value of 30.6 Å. The BET surface area extracted from argon sorption measurements was calculated to be 706 m^2^ g^−1^. AB stacked structure types were found unfeasible for both of the COFs as evident by comparison with the simulated patterns (Figures S47 and S50). It is noteworthy that all four COFs synthesized via the new ionothermal method show a comparable or even higher degree of crystallinity as compared to the solvothermally synthesized ones.^14^, ^17^ It is further important to note that energy‐dispersive X‐ray (EDX) spectroscopy revealed either none or only traces (<0.1 %) of zinc or chloride in all of the final COFs (Figures S53—S60). This is most likely due to the highly ordered framework structure of the COFs, which not only increases the reaction efficiency by increasing accessibility to the mobilized zinc and chloride ions but also renders removal of ZnCl_2_ easier after the COF formation as compared to amorphous CTF systems where ZnCl_2_ residues are extremely difficult to eliminate.2a, 33 Ionothermal synthesis thus appears as a promising approach to access crystalline imide COFs, but also other types of COFs built from highly stable linkages with limited reversibility.

## Conclusion

We have established a new ionothermal synthesis route for imide‐linked COFs, with which we were able to synthesize two crystalline and porous COFs (TAPB‐PTCDA‐ and TAPB‐PMDA‐COF) in pure ZnCl_2_. The COF formation is straightforward and does not require the use of toxic solvents and additional base catalysts and is also potentially scalable. It is rapid and happens within 10 h under these conditions as opposed to 3–7 days in classical solvothermal synthesis. Furthermore, by lowering the reaction temperature using a three salt eutectic mixture instead of pure ZnCl_2_, we were able to apply this ionothermal synthesis protocol to less stable linker molecules such as TAPA and TT. We could also show, using the example of TAPB‐PMDA‐COF, that crystalline adducts consisting of the respective precursors and ZnCl_2_ act as intermediates in the ionothermal synthesis of the COF. Formation of these intermediates possibly activates the anhydride and imide rings, thus lowering the overall activation barrier leading to a higher degree of reversibility in COF formation. We expect this new method of COF synthesis to greatly broaden the scope of imide‐linked COFs in particular and COFs based on inert linkages in general, which are inaccessible by traditional methods, but indispensable for future applications.

## Conflict of interest

The authors declare no conflict of interest.

## Supporting information

As a service to our authors and readers, this journal provides supporting information supplied by the authors. Such materials are peer reviewed and may be re‐organized for online delivery, but are not copy‐edited or typeset. Technical support issues arising from supporting information (other than missing files) should be addressed to the authors.

Supplementary
