# Supplementary material for: Ionothermal Synthesis of Imide‐Linked Covalent Organic Frameworks
Source: Angew Chem Int Ed Engl. 2020 Aug 11;59(36):15750–8. doi: 10.1002/anie.202007372 (PMC7497034; doi:10.1002/anie.202007372)
Supplement: Supplementary file 1 — Supplementary [file ANIE-59-15750-s001.pdf]

Angewandte Chemie
Eine Zeitschrift der Gesellschaft Deutscher Chemiker

Supporting Information

Ionothermal Synthesis of Imide-Linked Covalent Organic Frameworks

Johannes Maschita, Tanmay Banerjee, Gökcen Savasci, Frederik Haase, Christian Ochsenfeld, and Bettina V. Lotsch**

anie_202007372_sm_miscellaneous_information.pdf

SUPPORTING INFORMATION

Table of Contents

Experimental Procedures 2

FT-IR 2

Diffuse Reflectance Spectroscopy........ 2

Photoluminescence Spectroscopy........ 2

SEM/EDX........ 2

Sorption 2

XRPD........ 2

In situ XRPD 2

NMR 3

TEM 3

Quantum-Chemical Calculations........ 3

Scherrer Analysis........ 3

Materials 3

Synthesis 4

Results and Discussion **5**

References **48**

Author Contributions **48**

Experimental Procedures

FT-IR

Infrared spectra were recorded on a PerkinElmer UATR Two in attenuated total reflection (ATR) geometry equipped with a diamond crystal.

Diffuse Reflectance Spectroscopy

Diffuse reflectance UV–visible absorption spectra were collected on a Cary 5000 spectrometer (referenced to barium sulphate). Absorption spectra were calculated from the reflectance data using the Kubelka-Munk function.

Photoluminescence Spectroscopy

Steady-state emission data was collected at room temperature using an Edinburgh FLS980 spectrometer. Samples were excited using light output from a housed 450 W Xe lamp passed through a single grating (1800 l/mm, 250 nm blaze) Czerny-Turner monochromator and finally a bandwidth slit. Emission from the sample was passed through a double grating (1200 l/mm, 500 nm blaze) Czerny-Turner monochromator (appropriate bandwidth) and finally detected by a cooled microchannel plate photomultipler tube (MCP-PMT) detector.

SEM/EDX

SEM SE (secondary electron) detector images were obtained on either a Zeiss Merlin or a VEGA TS 5130MM (TESCAN) with a SEM-EDX using a Si/Li detector (10 kV acceleration voltage, Oxford).

Sorption

Sorption measurements were performed on a Quantachrome Instruments Autosorb iQ MP with Argon at 87 K. The pore size distribution was determined from argon adsorption isotherms using the quenched solid-state density functional theory (QSDFT) for cylindrical pores in carbon model for argon at 87 K.

XRPD

X-ray powder diffraction (XRPD) measurements were performed on a Stoe Stadi-P diffractometer in Debye-Scherrer geometry with Cu-$K_{\alpha 1}$ radiation equipped with a Ge(111) primary monochromator. The glass capillaries (0.7 mm in diameter) were spun during data collection for an improved particle statistics. Rietveld[1] refinements of the different COF structures were performed using TOPAS V5.[2] Model structures created by Material Studio were used for the Rietveld refinements with fixed atomic coordinates. The peak profile of the XRPD patterns was described by applying the fundamental parameter[3] approach as implemented in TOPAS. Lattice parameter (*a*, *b*-axes) were freely refined. The *c*-axis was taken from the calculation, since no reliable information about stacking distance was obtained from XRPD patterns. The background was modeled by Chebychev polynomials. The microstructure of the different COFs was modeled using microstrain (Lorentzian and Gaussian components).

In situ XRPD

In situ high temperature XRPD experiments were performed on a Bruker D8 diffractometer (Mo-$K_{\alpha 1}$ equipped with a primary Ge(220)-Johansson-type monochromator and a LynxEye position sensitive detector) in Debye-Scherrer geometry using a water cooled capillary furnace (mri Physikalische Geräte GmbH). The measurements were performed in the range of 2 – 25 ° 2θ with 1 h exposure time. For better particle statistics, the quartz capillary was spun during data collection. XRPD patterns were measured at RT, 180, 210, 240, 270, 290, 300 with staying at 300 °C for 10 h and then cool down to RT again.

NMR

Solid state nuclear magnetic resonance spectra (ssNMR) were recorded on a Bruker Avance III 400 MHz spectrometer (magnetic field 9.4 T). For ssNMR spectroscopy, the samples were packed in 4 mm ZrO_2 rotors, which were spun in a Bruker WVT BL4 double resonance MAS probe. Chemical shifts were referenced relative to tetramethylsilane. The spinning rate was 12-14 kHz and a standard cross-polarization sequence with a 2 ms ramped contact pulse was used for ^{13}C and a total of 4096-8192 scans were routinely accumulated. The measurements were performed under conditions of high-power broadband proton decoupling (SPINAL 64) with the spectral conditions being optimized for the shortest relaxation delay by measuring ^{1}H T_1 relaxation time.

TEM

TEM was performed with a Philips CM30 ST (300kV, LaB_6 cathode). The samples were prepared dry onto a copper lacey carbon grid (Plano). The line scan analysis was done with ImageJ, version 1.52a.The simulation of the projected potential map was performedwith the jems package (Stadelmann).

Quantum-Chemical Calculations

Atom positions and lattices of all periodic structures were optimized on RI-PBE-D3/def2-TZVP[4] level of theory using an acceleration scheme based on the resolution of the identity (RI) technique and the continuous fast multipole method (CFMM[5]) implemented[6] in Turbomole[7] version V7.3.
The CFMM uses multipole moments of maximum order 20, together with a well-separateness value of 3 and a basis function extent threshold of 10E-9 a.u. Grid 7 was used for the numerical integration of the exchange-correlation term. The norm of the gradient was converged to 10E-4 a.u. and the total energy to 10E-8 Hartree within the structure optimization using the gamma point approximation. Structures for all investigated molecular compounds were optimized on PBE0-D3/def2-TZVP[4a, 4d, 8] level of theory. Subsequent frequency calculations were performed on the same level of theory to ensure all minima to be true minima on the potential energy hypersurface. Partial Charges were extracted from the summary of a subsequent Natural Population Analysis[9] on the same level of theory.
NMR chemical shifts were obtained on B97-2/pcSseg-2[10] level of theory using the FermiONs++ program package[11].

Scherrer Analysis

To determine the mean size of the crystalline domains Scherrer analysis was performed using the Scherrer equation displayed below. All reflections of the COFs or phases of the *in situ* measurement without a significant overlap with other reflections were fitted using the Voigt function model. FWHM and Bragg angles were received from the corresponding fit function and inserted into the Scherrer equation together with the respective wavelength and the shape factor fixed at K = 0.9. To yield the most accurate crystallite sizes as many reflections as possible were considered for the final calculations. Since the quality of the synthesized COFs varies significantly, not every COF could be described with the same number of reflections.

Scherrer Equation:

$$\tau = \frac{K\lambda}{\beta \cos\theta}$$

τ = Mean size of the crystalline domains
K = Shape factor (K = 0.9)
λ = X-ray wavelength (Cu-Kα1 = 1.540596 nm or Mo-Kα1 = 0.7107 nm)
β = line broadening at half the maximum intensity (FWHM)
θ = Bragg angle

Materials

Tris(4-aminophenyl)triazine was synthesized according to a literature procedure.[12] All other chemicals were obtained from commercial sources and were used as received.

Synthesis

Ionothermal Synthesis of PI-COFs in $ZnCl_2$

Synthesis of TAPB-PMDA-COF. Typically, pyromelltic dianhydride (21.8 mg, 0.1 mmol), 1,3,5-Tris(4-aminophenyl)benzene (25.2 mg, 0.67 mmol) and anhydrous zinc chloride (114 mg, 0.83 mmol) were ground thoroughly in a mortar under inert atmosphere and transferred into a quartz tube. The quartz tube was evacuated (< 10^{-2} mbar), flame sealed and heated to 300 °C for 48 h in a tube furnace. The ampoule was allowed to cool down to room temperature and opened. The crude product was ground carefully in a mortar and subsequently washed with 1M HCl, water and THF. Solvent exchange was carried out by Soxhlet extraction with methanol overnight. The solvent was removed via supercritical CO_2 drying to afford TAPB-PMDA-COF as a dark brown powder.

Synthesis of TAPB-PTCDA-COF. Typically, Perylene-3,4,9,10-tetracarboxylic dianhydride (31.2 mg, 0.08 mmol), 1,3,5-Tris(4-aminophenyl)benzene (20 mg, 0.053 mmol) and anhydrous zinc chloride (722 mg, 5.3 mmol) were ground thoroughly in a mortar under inert atmosphere and transferred into a quartz tube. The quartz tube was evacuated (< 10^{-2} mbar), flame sealed and heated to 300 °C for 48 h in a tube furnace. The ampoule was allowed to cool down to room temperature and opened. The crude product was ground carefully in a mortar and subsequently washed with 1M HCl, water and THF. Solvent exchange was carried out by Soxhlet extraction with methanol overnight. The solvent was removed via supercritical CO_2 drying to afford TAPB-PTCDA-COF as a dark brown powder.

Ionothermal Synthesis of PI-COFs in eutectic salt mixture

Synthesis of TT-PMDA-COF. First, sodium chloride (12.4 mg, 0.21 mmol), potassium chloride (39.5 mg, 0.53 mmol) and anhydrous zinc chloride (114 mg, 0.83 mmol) were ground together thoroughly in a mortar under inert conditions. Then, pyromelltic dianhydride (21.8 mg, 0.1 mmol) and 1,3,5-Tris(4-aminophenyl)triazine (23.6 mg, 0.067 mmol) were added and the mixture was ground again to achieve homogeneity and transferred into a quartz tube. The quartz tube was evacuated (< 10^{-2} mbar), flame sealed and heated to 250 °C for 48 h in a tube furnace. The ampoule was allowed to cool down to room temperature and opened. The crude product was ground carefully in a mortar and subsequently washed with 1M HCl, water and THF. Solvent exchange was carried out by Soxhlet extraction with methanol overnight. The solvent was removed via supercritical CO_2 drying to afford TT-PMDA-COF as a light brown - yellow powder.

Synthesis of TAPA-PMDA-COF. First, sodium chloride (12.4 mg, 0.21 mmol), potassium chloride (39.5 mg, 0.53 mmol) and anhydrous zinc chloride (114 mg, 0.83 mmol) were ground together in a mortar thoroughly under inert conditions. Then, pyromellitic dianhydride (21.8 mg, 0.1 mmol) and Tris-(4-aminophenyl)amine (19.3 mg, 0.067 mmol) were added and the mixture was ground again to achieve homogeneity and transferred into a quartz tube. The quartz tube was evacuated (< 10^{-2} mbar), flame sealed and heated to 250 °C for 48 h in a tube furnace. The ampoule was allowed to cool down to room temperature and opened. The crude product was ground carefully in a mortar and subsequently washed with 1M HCl, water and THF. Solvent exchange was carried out by Soxhlet extraction with methanol overnight. The solvent was removed via supercritical CO_2 drying to afford TT-PMDA-COF as a brown powder.

Results and Discussion

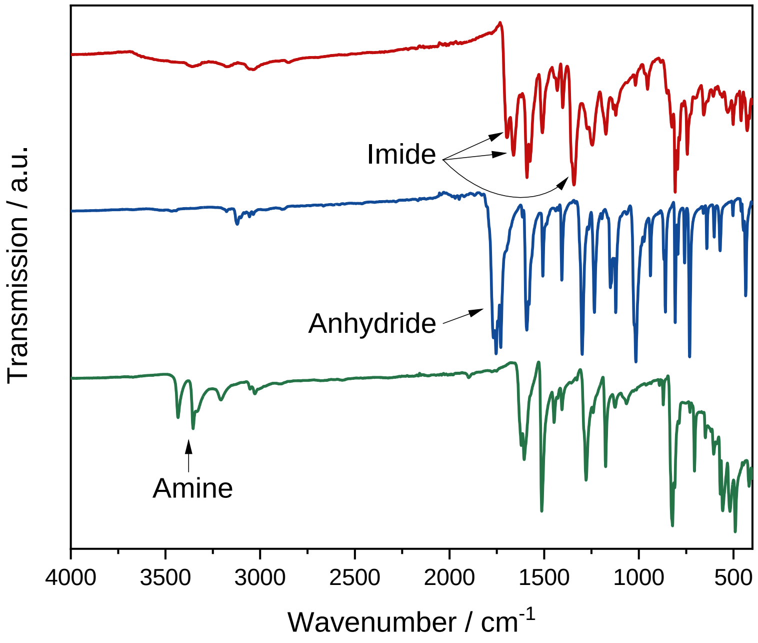

Figure S1. FT-IR spectra of **TAPB-PTCDA-COF** (red) and its precursor molecules PTCDA (blue) and TAPB (green). The absence of amine and anhydride vibrations in the COF indicate complete imide formation.

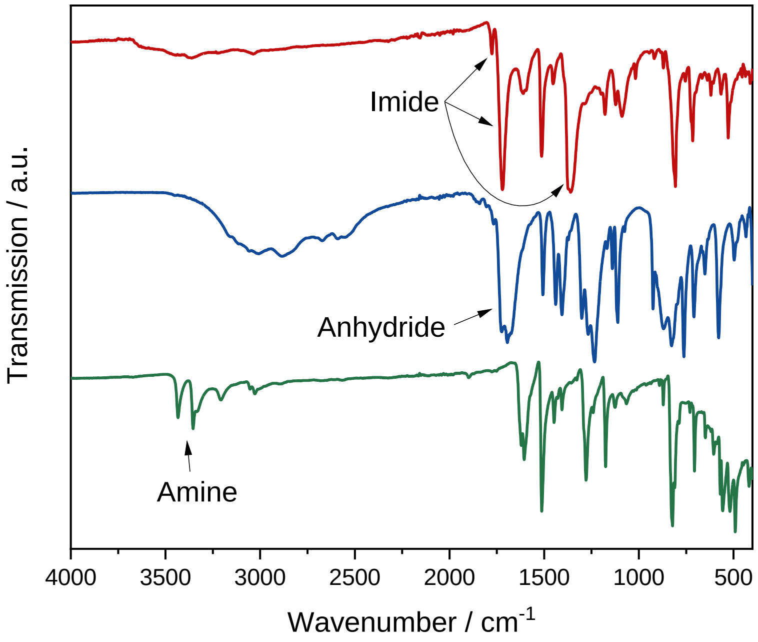

Figure S2. FT-IR spectra of **TAPB-PMDA-COF** (red) and its precursor molecules PMDA (blue) and TAPB (green). The absence of amine or anhydride vibrational bands in the COF indicate complete imide formation.

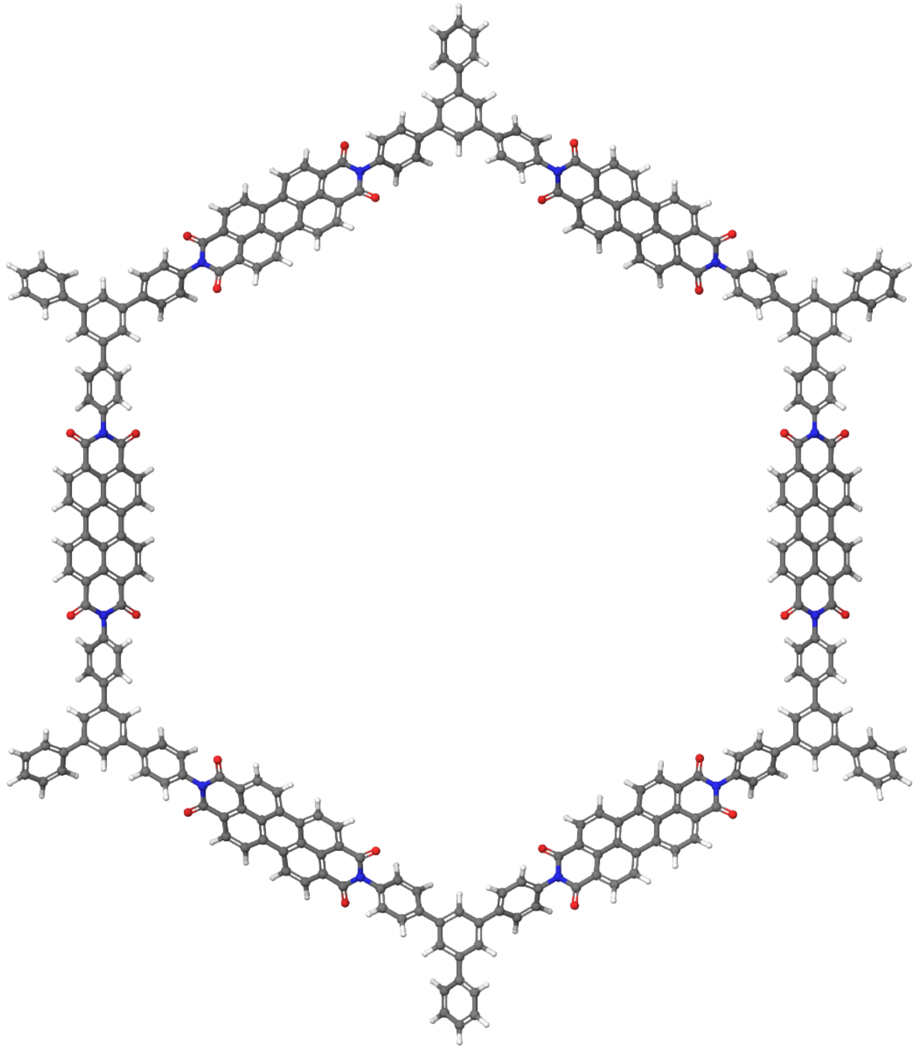

Figure S3. Structure of a single pore of the **TAPB-PTCDA** COF, obtained by composing a supercell from a 2D periodic geometry optimization of the corresponding asymmetric unit on RI-PBE-D3/def2-TZVP level of theory.

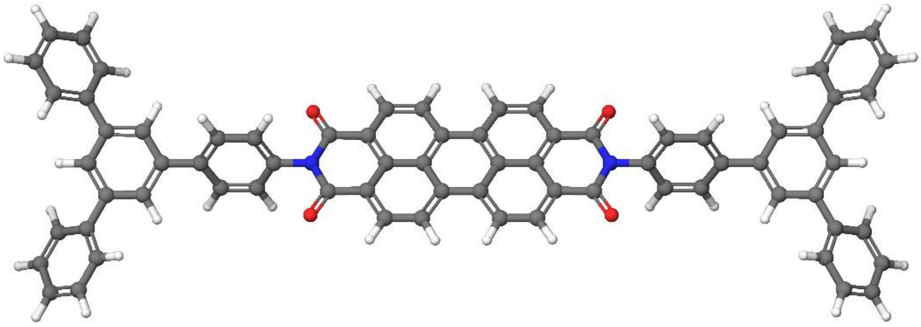

Figure S4. Structure of the **TAPB-PTCDA** NMR model, modeled by cutting the supercell obtained from the 2D optimized structure of the TAPB-PTCDA COF model.

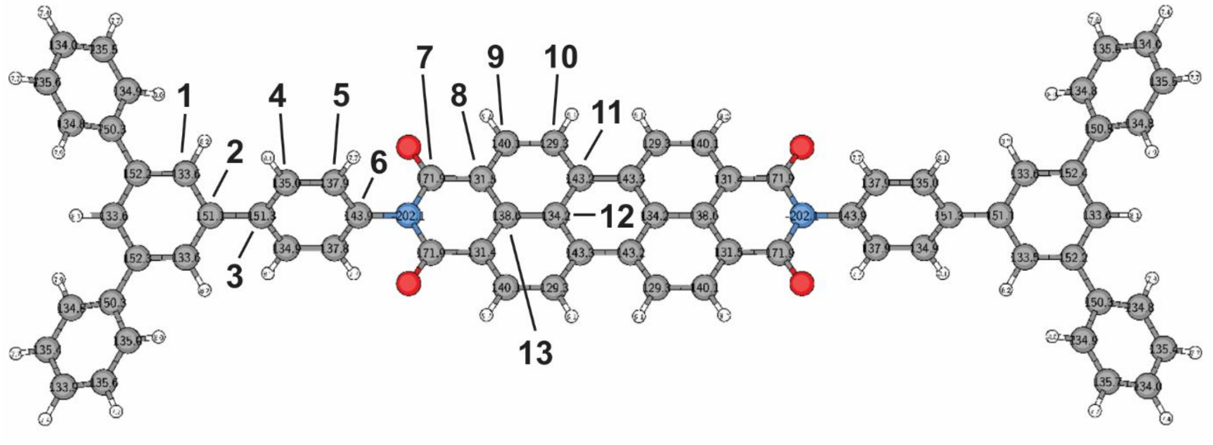

Figure S5. Calculated NMR chemical shifts for the **TAPB-PTCDA** NMR model, obtained on B97-2/pcsSeg-2 level of theory.

Table S1. Calculated NMR chemical shifts for the **TAPB-PTCDA** NMR model, obtained on B97-2/pcsSeg-2 level of theory.

Atom Label	Atom	NMR Chemical Shift [ppm]
1	C	133.6
2	C	151.1
3	C	151.3
4	C	135.0
5	C	137.9
6	C	143.9
7	C	171.9
8	C	131.5
9	C	140.1
10	C	129.3
11	C	143.2
12	C	134.2
13	C	138.6

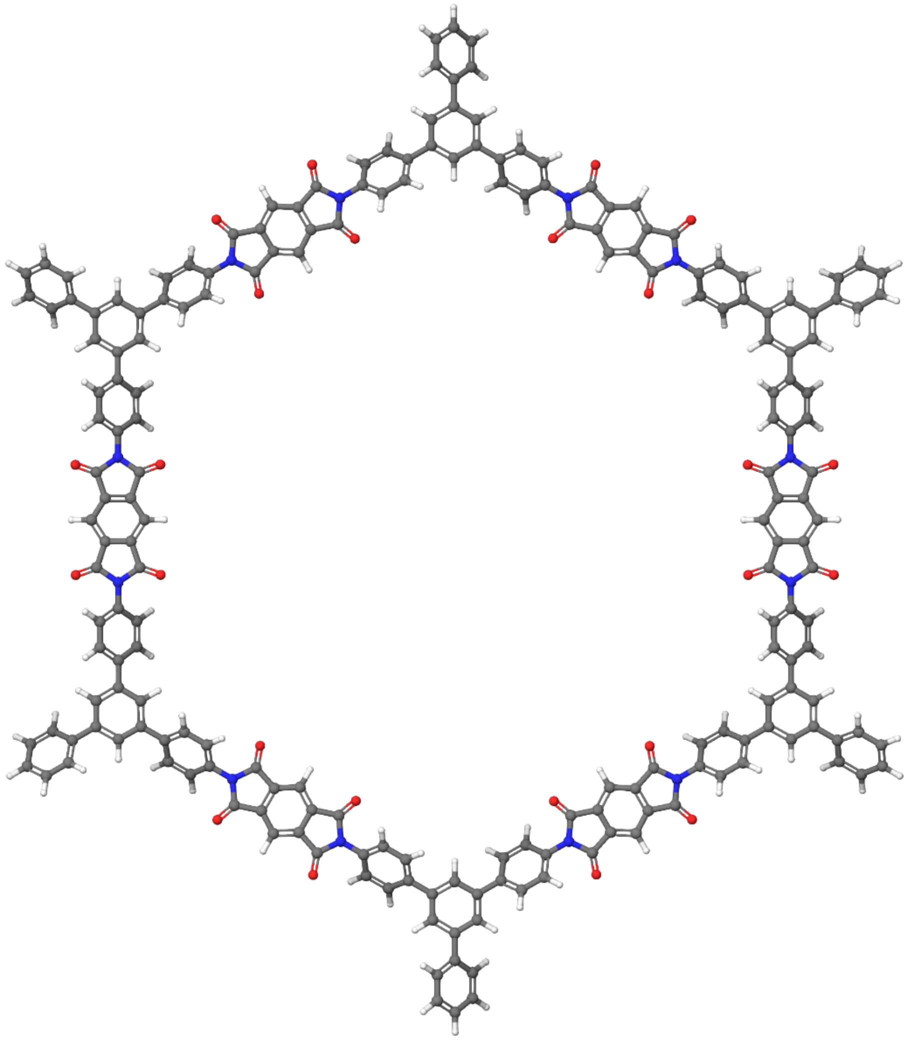

Figure S6. Structure of a single pore of the **TAPB-PMDA** COF, obtained by composing a supercell from a 2D periodic geometry optimization of the corresponding asymmetric unit on RI-PBE-D3/def2-TZVP level of theory.

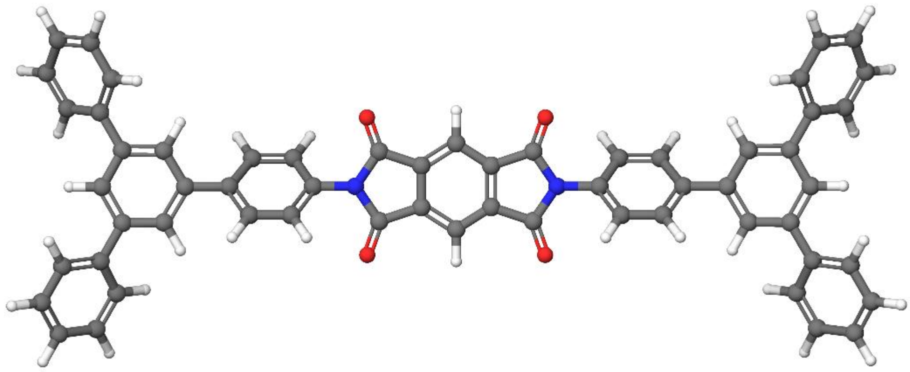

Figure S7. Structure of the **TAPB-PMDA** NMR model, modeled by cutting the supercell obtained from the 2D optimized structure of the TAPB-PMDA COF model.

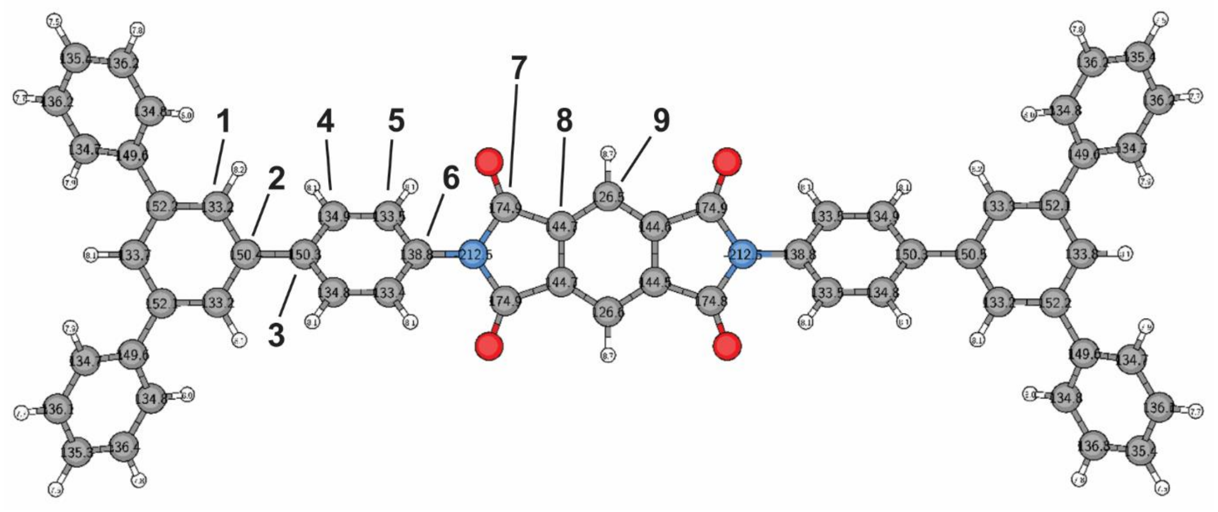

Figure S8. Calculated NMR chemical shifts for the **TAPB-PMDA** NMR model, obtained on B97-2/pcsSeg-2 level of theory.

Table S2. Calculated NMR chemical shifts for the **TAPB-PMDA** NMR model, obtained on B97-2/pcsSeg-2 level of theory.

Atom Label	Atom	NMR Chemical Shift [ppm]
1	C	133.2
2	C	150.2
3	C	150.3
4	C	134.9
5	C	133.5
6	C	138.8
7	C	174.9
8	C	144.7
9	C	126.5

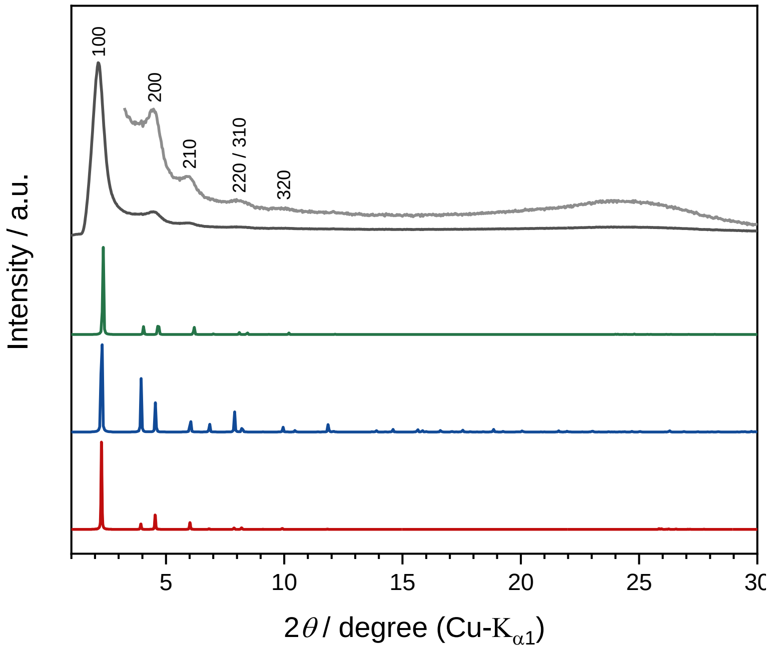

Figure S9. Experimental XRPD pattern (λ = Cu-$K_{\alpha1}$) of **TAPB-PTCDA-COF** (black) compared to simulated patterns of the COF assuming $P\bar{3}1m$ symmetry (green), $P\bar{3}1c$ symmetry (blue) and *Cmcm* symmetry (red).

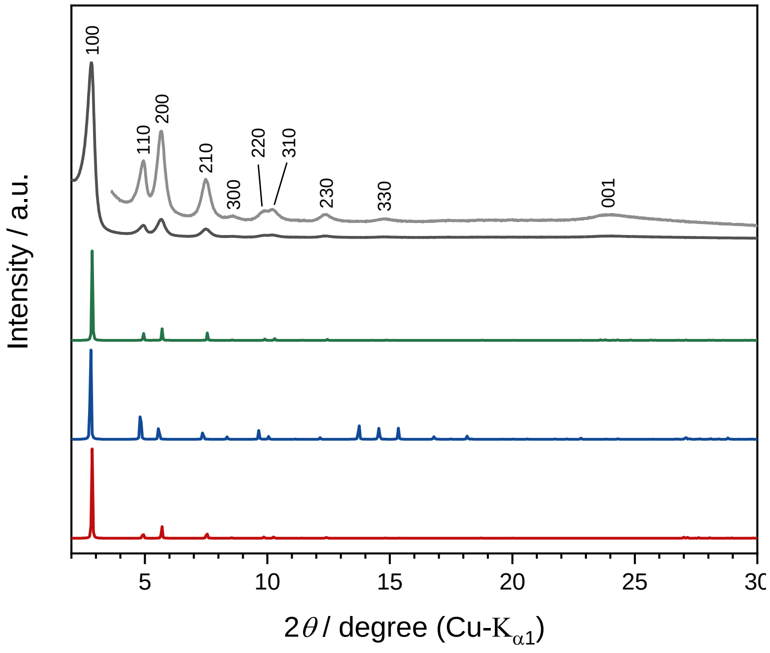

Figure S10. Experimental XRPD pattern (λ = Cu-$K_{\alpha1}$) of **TAPB-PMDA-COF** (black) compared to simulated patterns of the COF assuming $P\bar{3}1m$ symmetry (green), $P\bar{3}1c$ symmetry (blue) and *Cmcm* symmetry (red).

Table S3. Rietveld refinement of TAPB-PTCDA- and TAPB-PMDA-COF at room temperature.

Fitted Pattern	TAPB-PTCDA-COF	TAPB-PMDA-COF
**Space group**	$P\bar{3}1m$	$P\bar{3}1m$
**Constraints**	*c* = fixed	-
**Rwp (%)**	3.922	4.986
**Cell Volume (Å³)**	6100(3)	4164(9)
**A (Å)**	43.6(2)	35.7(4)
**B (Å)**	43.6(2)	35.7(4)
**C (Å)**	3.705	3.76(5)
**α (°)**	90	90
**β (°)**	90	90
**γ (°)**	120	120

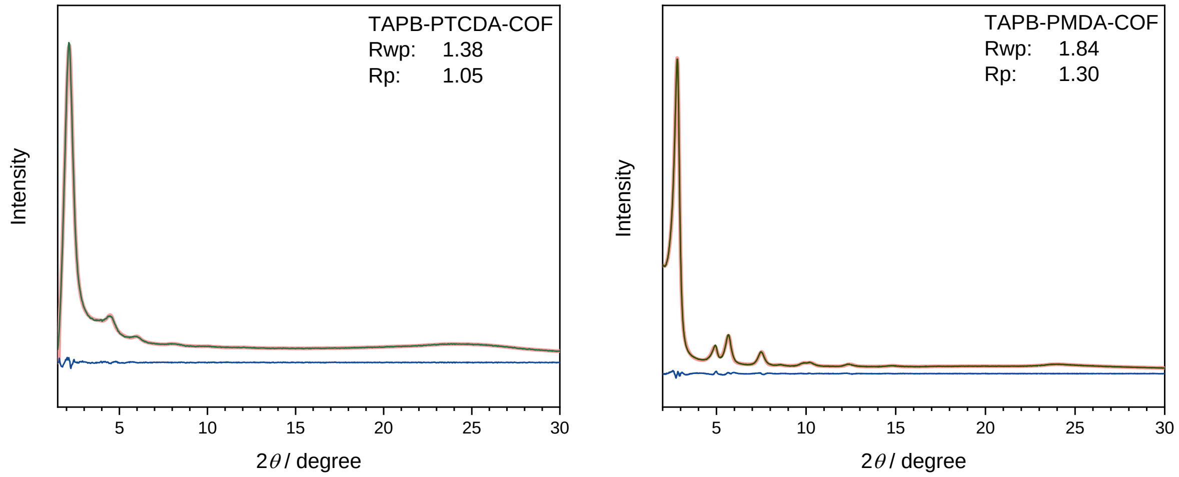

Figure S11. Pawley refinement of TAPB-PTCDA-COF (left) and TAPB-PMDA-COF (right) assuming $P\bar{3}1m$ symmetry with fixed lattice sizes and fit parameters obtained from Rietveld refinement.

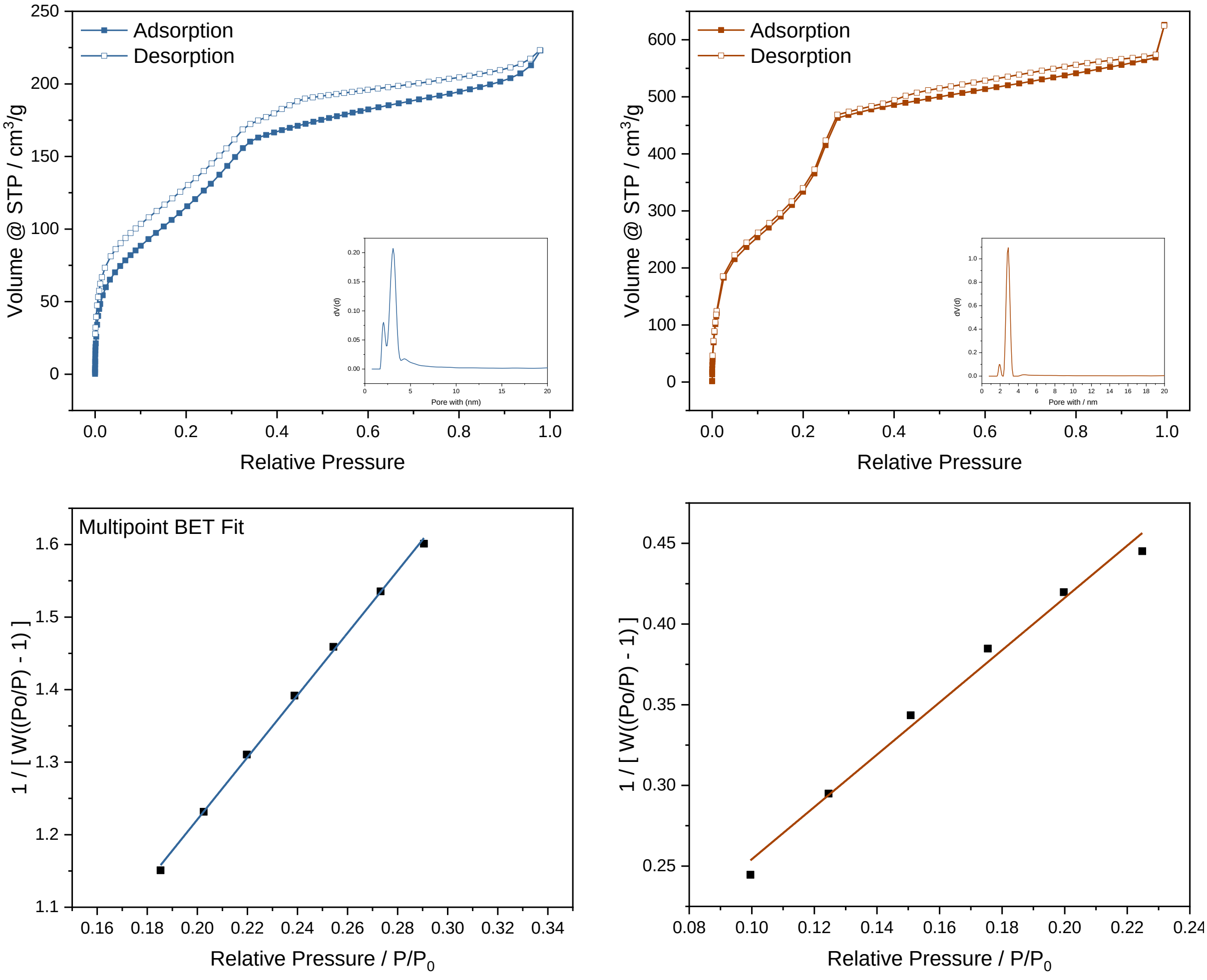

Figure S12. Top: Argon gas adsorption and desorption isotherms for TAPB-PTCDA (blue) and TAPB-PMDA-COF (brown). Insets: Respective calculated pore size distributions of PI-COFs from fitting the quenched solid-state density functional theory (QSDFT) model. Bottom: Multipoint BET fits of TAPB-PTCDA (blue) and TAPB-PMDA (brown).

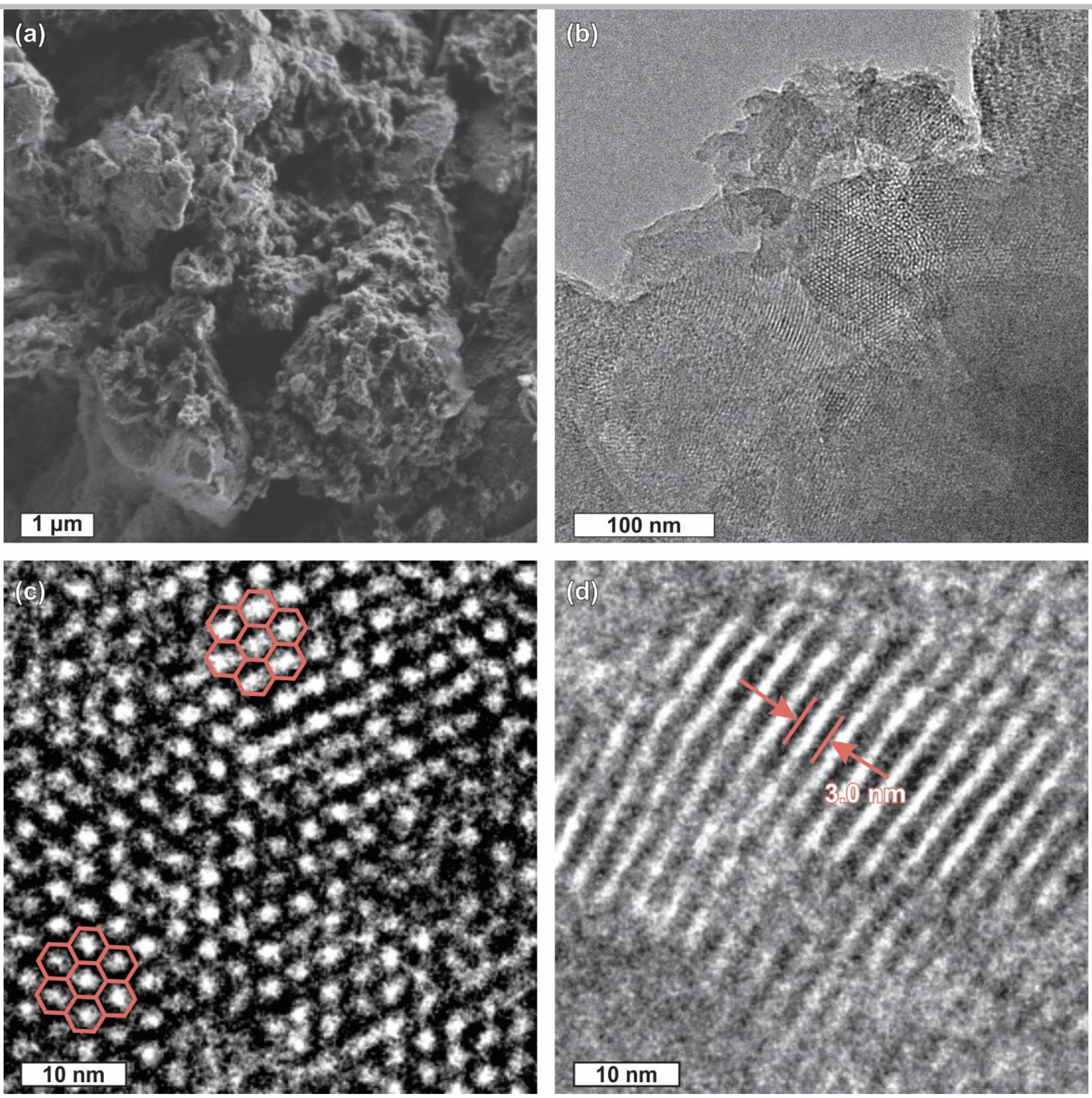

Figure S13. SEM image (a) and TEM images (b) – (d) of **TAPB-PMDA-COF**. (c) shows the hexagonal pores highlighted in red and (d) shows straight channels with a diameter of ~ 3.0 nm.

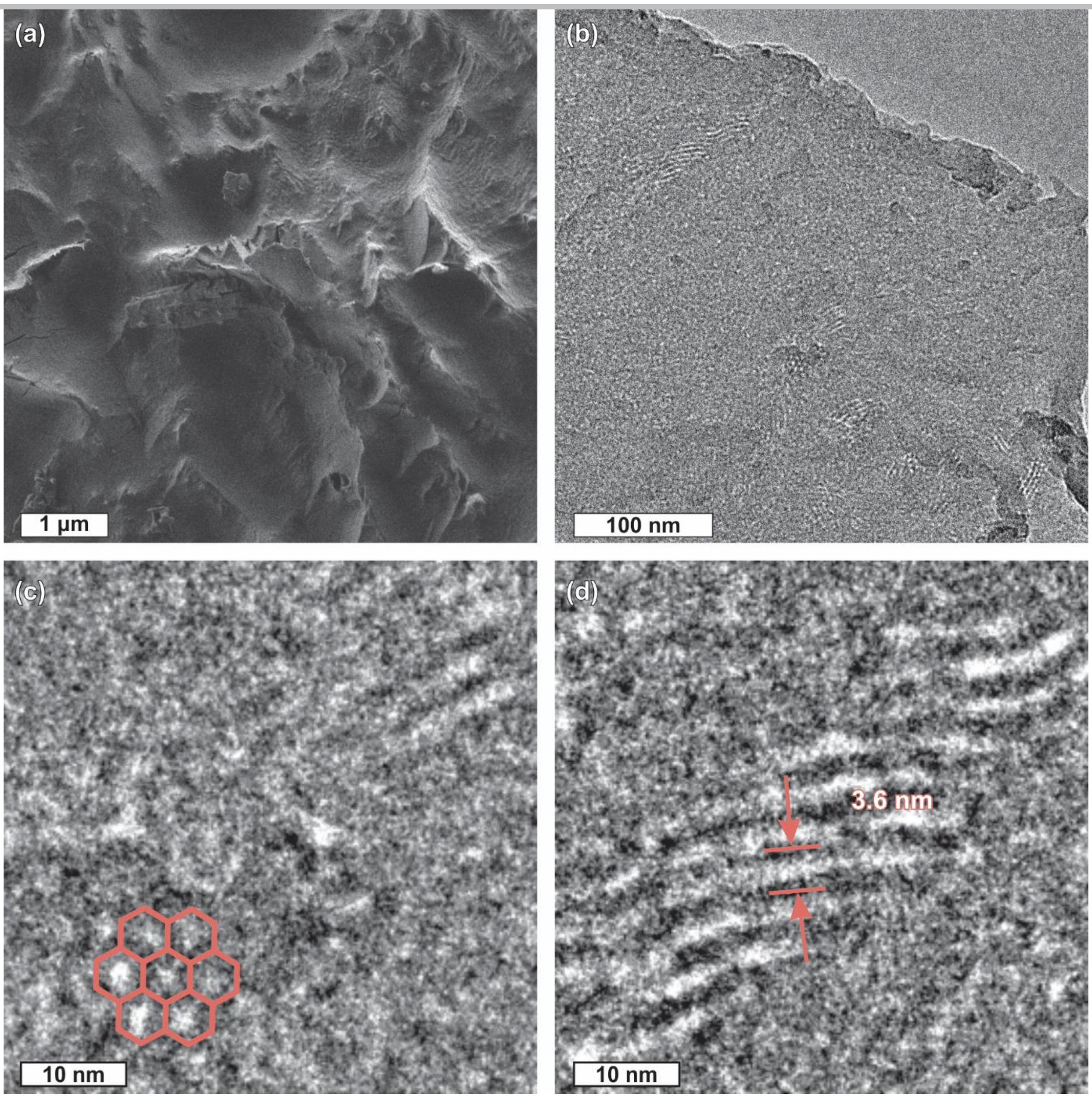

Figure S14. SEM image (a) and TEM images (b) – (d) of **TAPB-PTCDA-COF**. (c) shows the hexagonal pores highlighted in red and (d) shows straight pore channels with a diameter of ~ 3.6 nm.

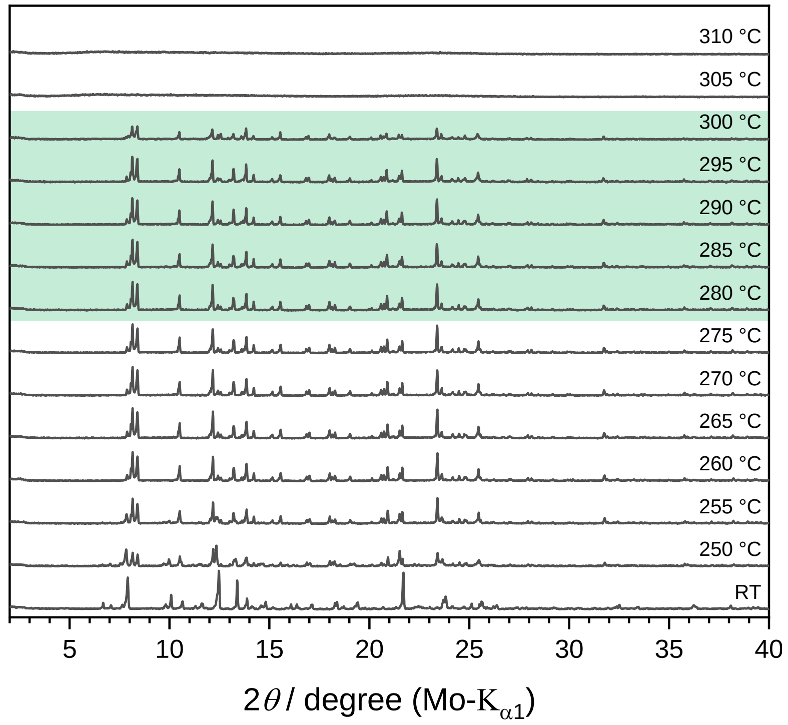

Figure S15. Melting point determination of $ZnCl_2$ *via* high-temperature XRPD. The melting point is indicated by the complete vanishing of the X-ray reflections between 300 and 305 °C. The green area shows the temperature window, in which the COF formation in pure $ZnCl_2$ takes place.

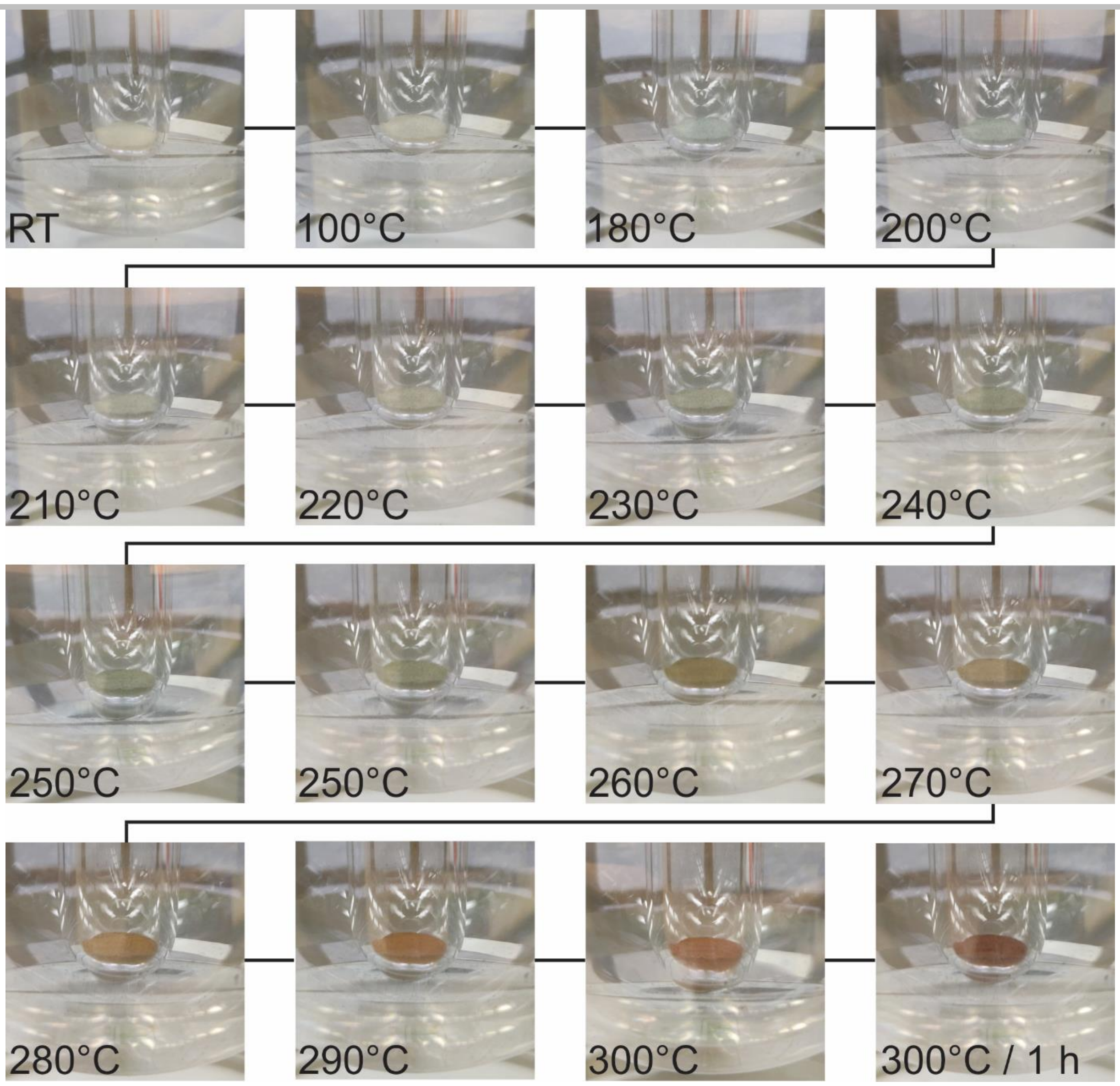

Figure S16. Visual observation of the TAPB-PMDA-COF formation reaction showing the absence of a solution or melt involved in the reaction process. The reaction mixture was heated stepwise to the respective temperatures. After reaching the respective temperatures, the system was allowed to equilibrate for 15 min before the pictures were taken.

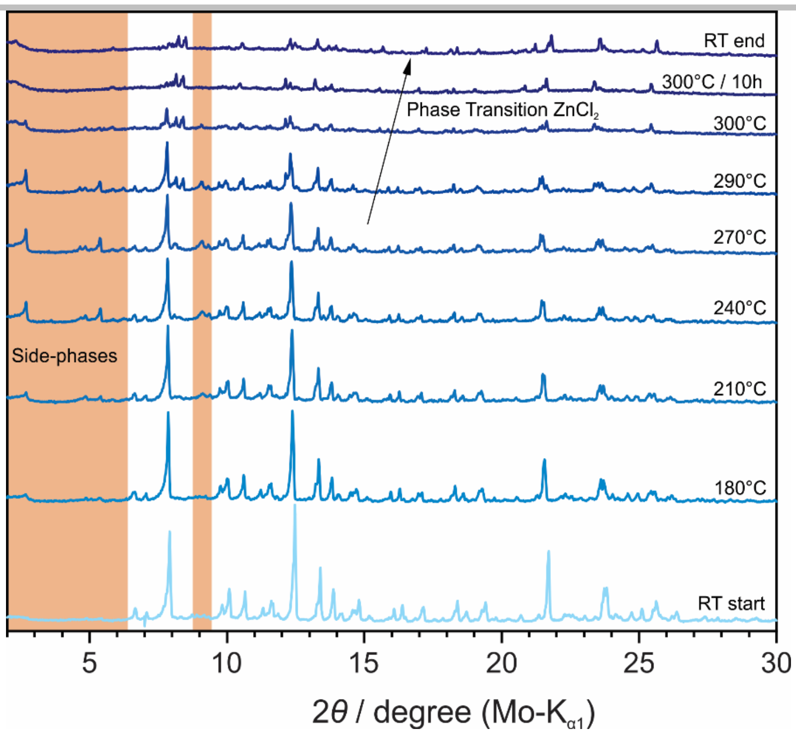

Figure S17. *In situ* HT-XRPD patterns (λ = Mo-K$_{\alpha1}$) of the COF precursor mixture (TAPB, PMDA, $ZnCl_2$) revealing a dramatic change of the appearing reflections over time and temperature. The pattern at room temperature is dominated by the reflections of zinc chloride, which undergoes a phase transition at elevated temperatures. Besides that side phases can be observed to appear at 180 °C, which get more prominent with increasing temperature till 290 °C. At 300 °C the side phases start vanishing again.

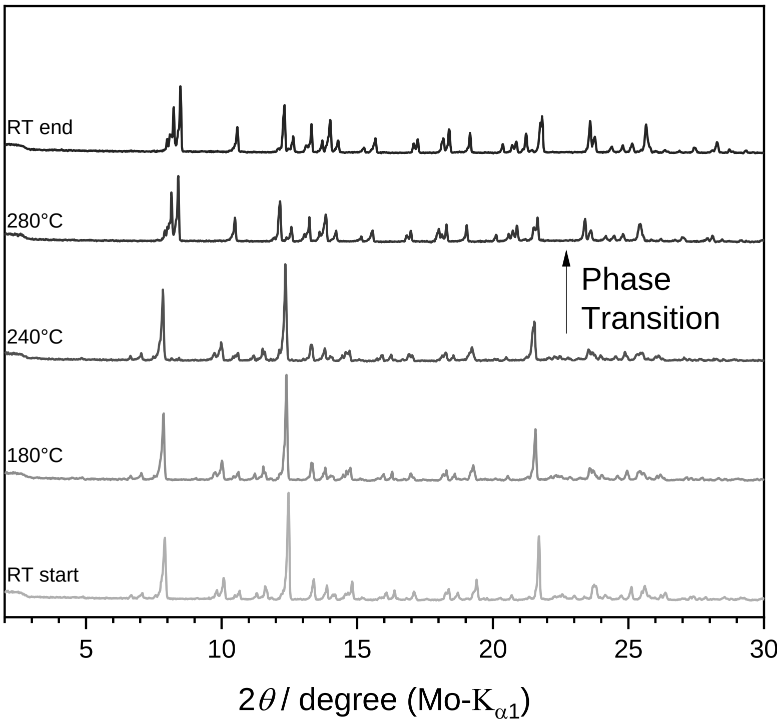

Figure S18. *In situ* HT-XRPD patterns (λ = Mo-K$_{\alpha1}$) of pure zinc chloride showing a phase transition from the monoclinic γ-form to the orthorhombic δ-form above 240 °C.

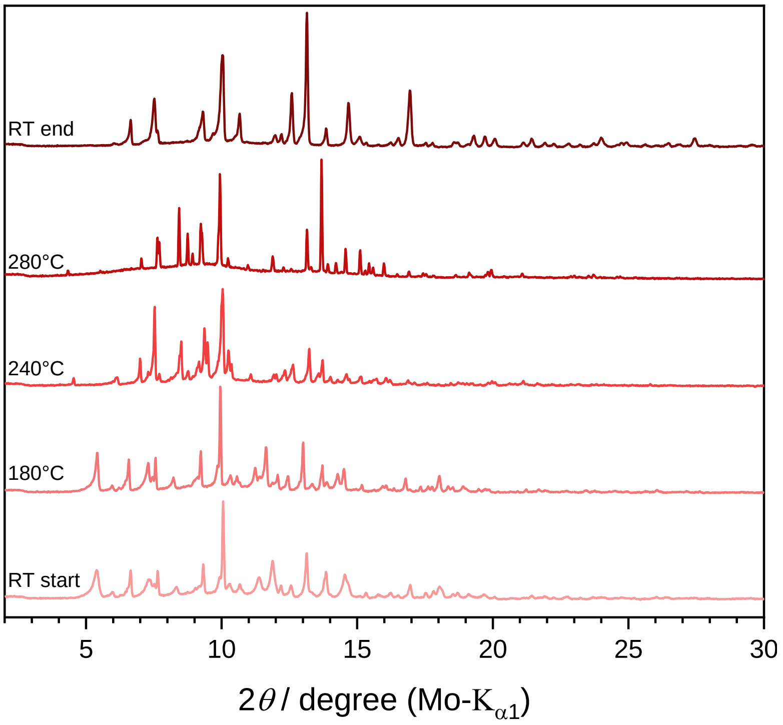

Figure S19. *In situ* HT-XRPD patterns (λ = Mo-$K_{\alpha1}$) of pure PMDA showing a row of structural changes with proceeding time and temperature.

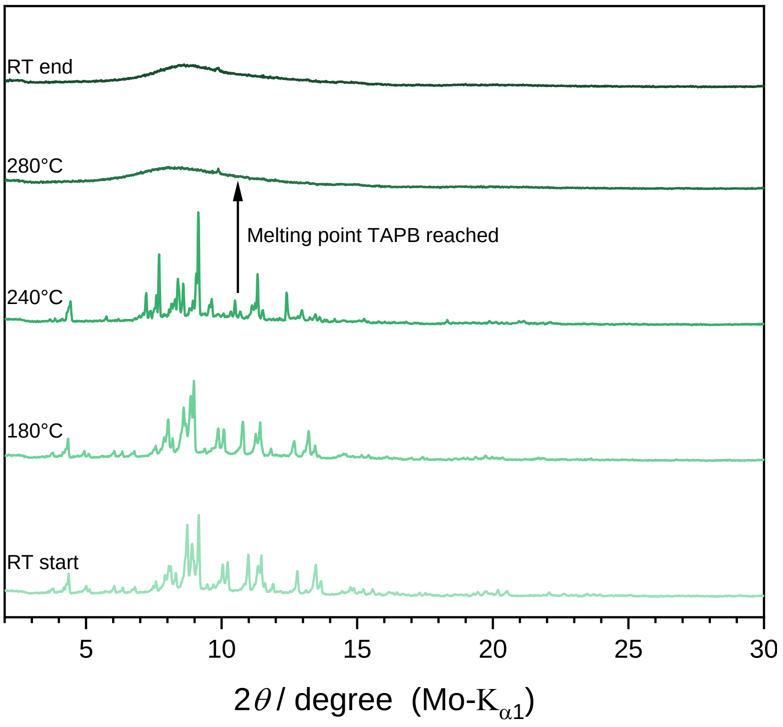

Figure S20. *In situ* HT-XRPD patterns (λ = Mo-$K_{\alpha1}$) of pure TAPB revealing the complete loss of reflections above 240 °C due to melting of the precursor.

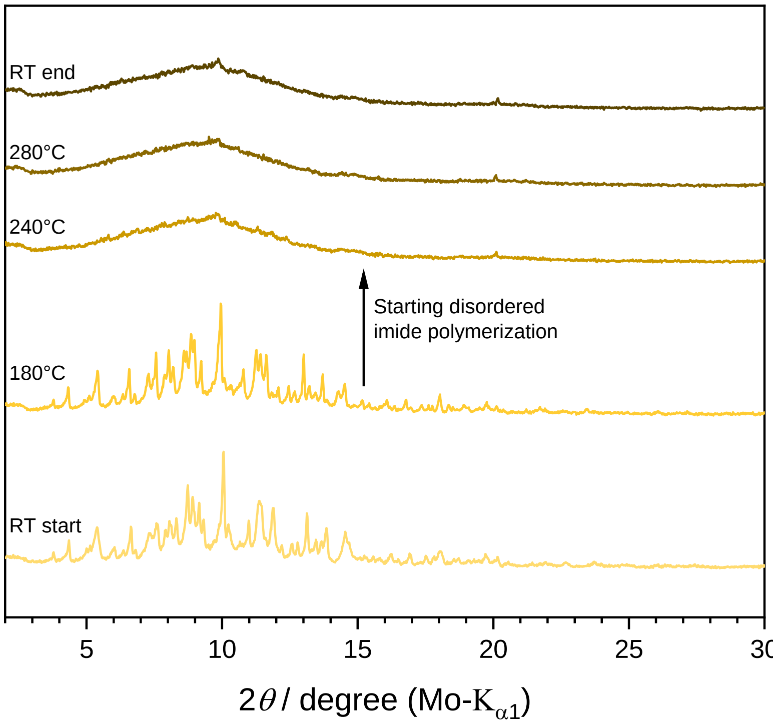

Figure S21. *In situ* HT-XRPD patterns (λ = Mo-$K_{\alpha1}$) of a mixture of TAPB and PMDA showing reflections of both precursor molecules up to 180 °C. In addition the patterns reveal a complete loss of all reflections between 180 and 240 °C. This is due to the starting disordered imide polymerization as evident from the screening experiments described in the main text.

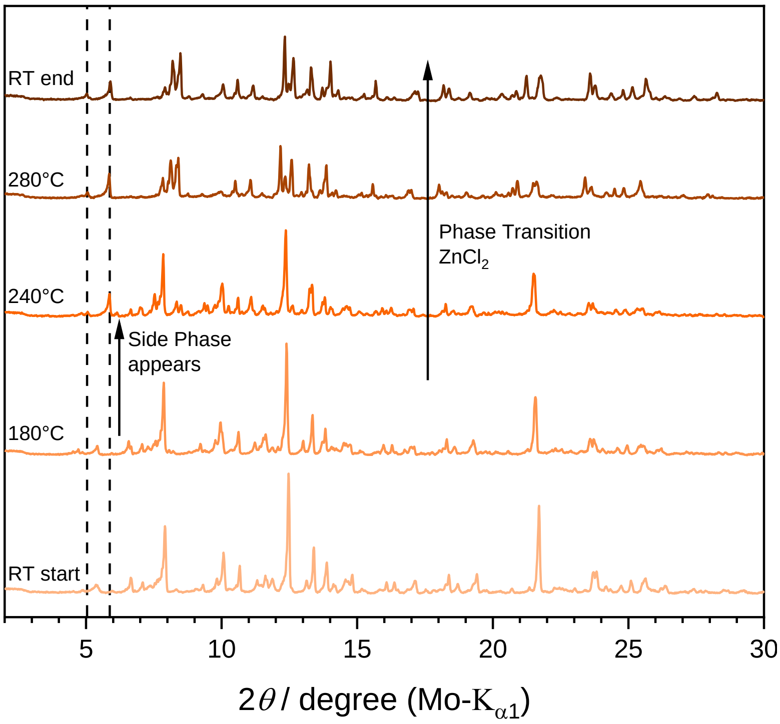

Figure S22. *In situ* HT-XRPD patterns (λ = Mo-$K_{\alpha1}$) of a mixture of PMDA and $ZnCl_2$. The patterns are dominated by the reflections of zinc chloride that undergoes phase transition from the monoclinic γ-form to the orthorhombic δ-form 240 °C upwards. An appearing side-phase can be identified, which were observed neither in the diffraction pattern of pure PMDA nor in the pattern of pure zinc chloride hinting towards a reaction between PMDA and $ZnCl_2$ to form an intermediate species.

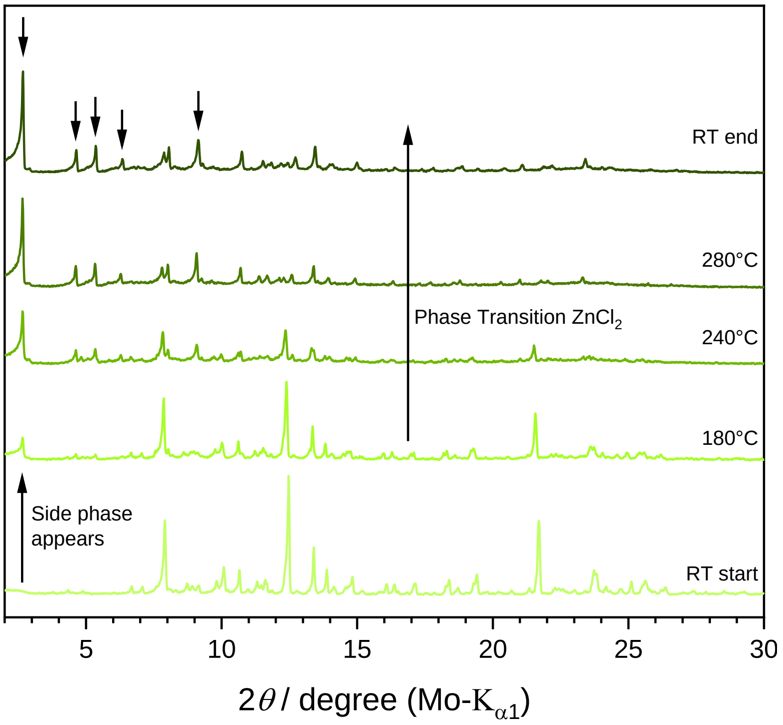

Figure S23. *In situ* HT-XRPD patterns (λ = Mo-$K_{\alpha1}$) of a mixture of TAPB and $ZnCl_2$. At room temperature, the patterns are dominated by the reflections of zinc chloride that undergoes phase transition from the monoclinic γ-form to the orthorhombic δ-form 240 °C upwards. At 180 °C, an appearing side-phase can be identified, which were observed neither in the diffraction pattern of pure TAPB nor in the pattern of pure zinc chloride hinting towards a reaction between TAPB and $ZnCl_2$ to form an intermediate species. With increasing temperature the reflections of the observed side-phase intensify and dominate the reflections of $ZnCl_2$ emphasizing the highly oriented structure of this species.

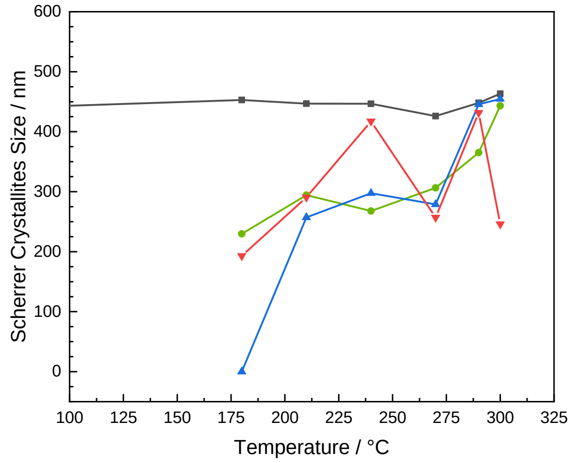

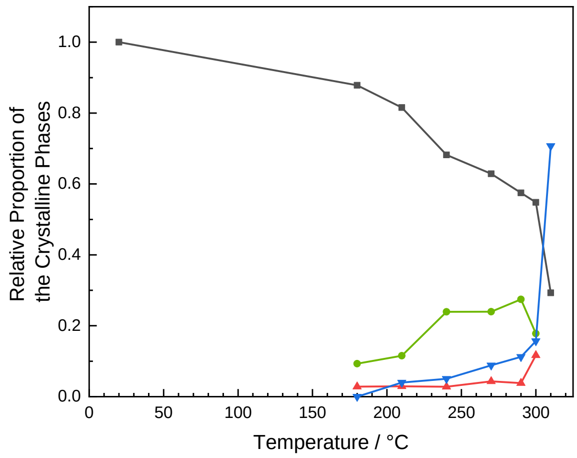

Figure S24. Calculated Scherrer crystallite sizes (left) and calculated relative amounts (right) of the individual crystalline domains (γ-$ZnCl_2$ (black), δ-$ZnCl_2$ (blue), TAPB-adduct (green), PMDA-adduct (red)) at different temperatures from the *in situ* high temperature XRD measurement. Scherrer analysis of the *in situ* measurement revealed that the crystallite size of the γ-$ZnCl_2$ (black) remains constant at 450 nm with increasing temperature, while the relative amount of γ-$ZnCl_2$ decreases. The constant crystallite size of this $ZnCl_2$ phase confirms that no melting takes place. In addition, a successive increase of the crystallite size and relative amounts of the intermediate phases with a maximum at the optimized reaction temperature (280 – 300 °C) can be observed. Two effects can explain the decreasing relative amount of the γ-$ZnCl_2$ phase: I) At 180°C and higher the $ZnCl_2$ reacts with the precursor molecules to form the $ZnCl_2$ – precursor adducts (green and red) resulting in a successive increase of the crystallite size and relative amount of the precursor adducts in the same temperature region. II) The phase transition from γ-$ZnCl_2$ to δ-$ZnCl_2$ proceeds quickly above 290 °C and results in an inversion of the relative amounts of the respective phase (blue vs. black).

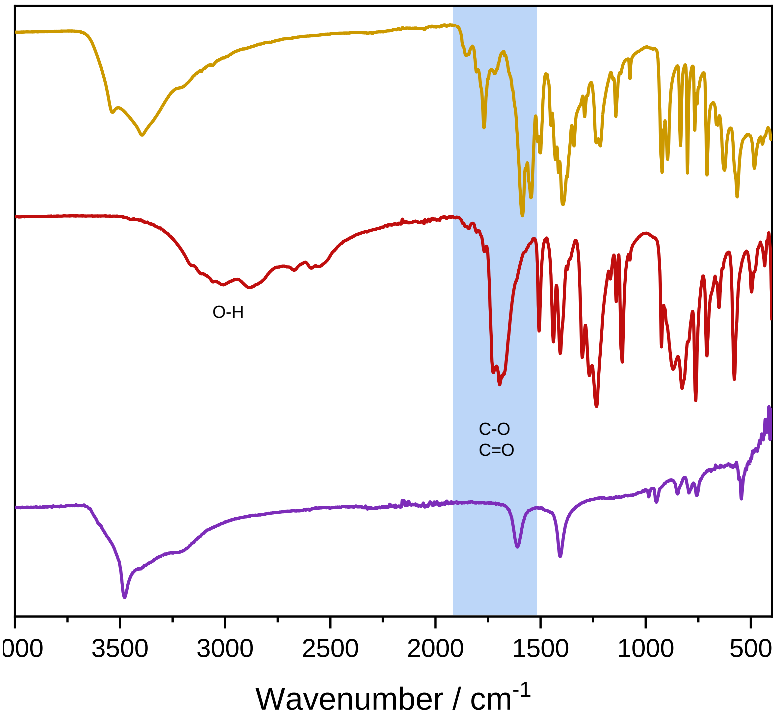

Figure S25. FT-IR spectra of $ZnCl_2$ (purple), PMDA (red) and the corresponding intermediate species (yellow), showing a shift of the C-O and C=O vibrational bands indicating the formation of an PMDA-$ZnCl_2$ adduct.

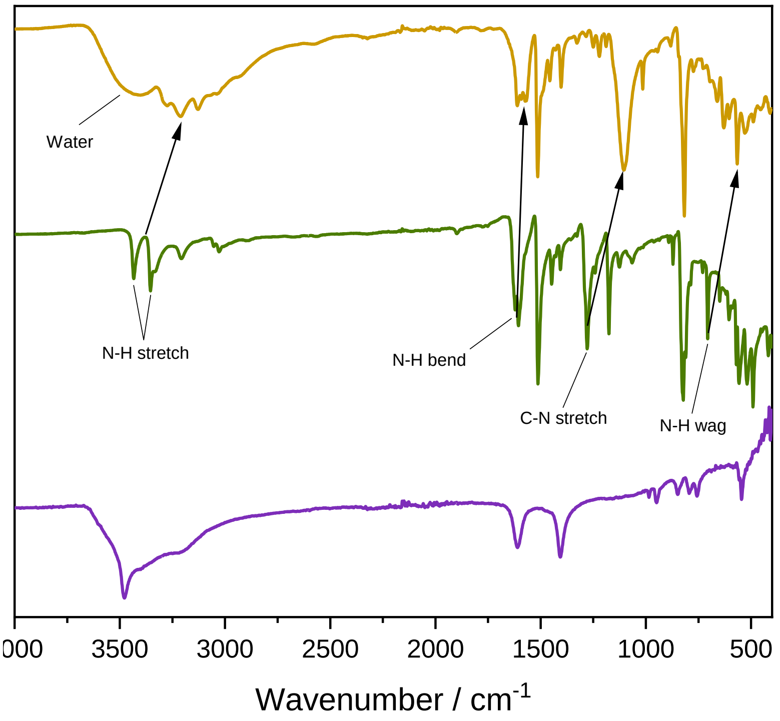

Figure S26. FT-IR spectra of $ZnCl_2$ (purple), PMDA (red) and the corresponding intermediate species (yellow), showing a shift of all amine-related vibrational bands towards lower wavenumber indicating the formation of an TAPB-$ZnCl_2$ adduct.

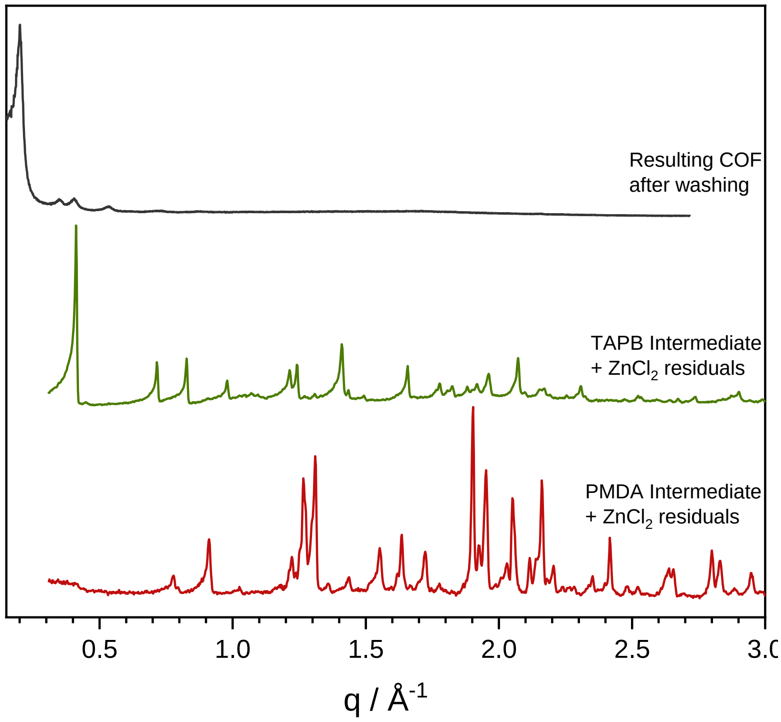

Figure S27. XRPD Patterns of the separately synthesized intermediate species of PMDA (red) and TAPB (green) and the resulting COF (black) after combining the intermediates and heating to 290 °C for 20 h.

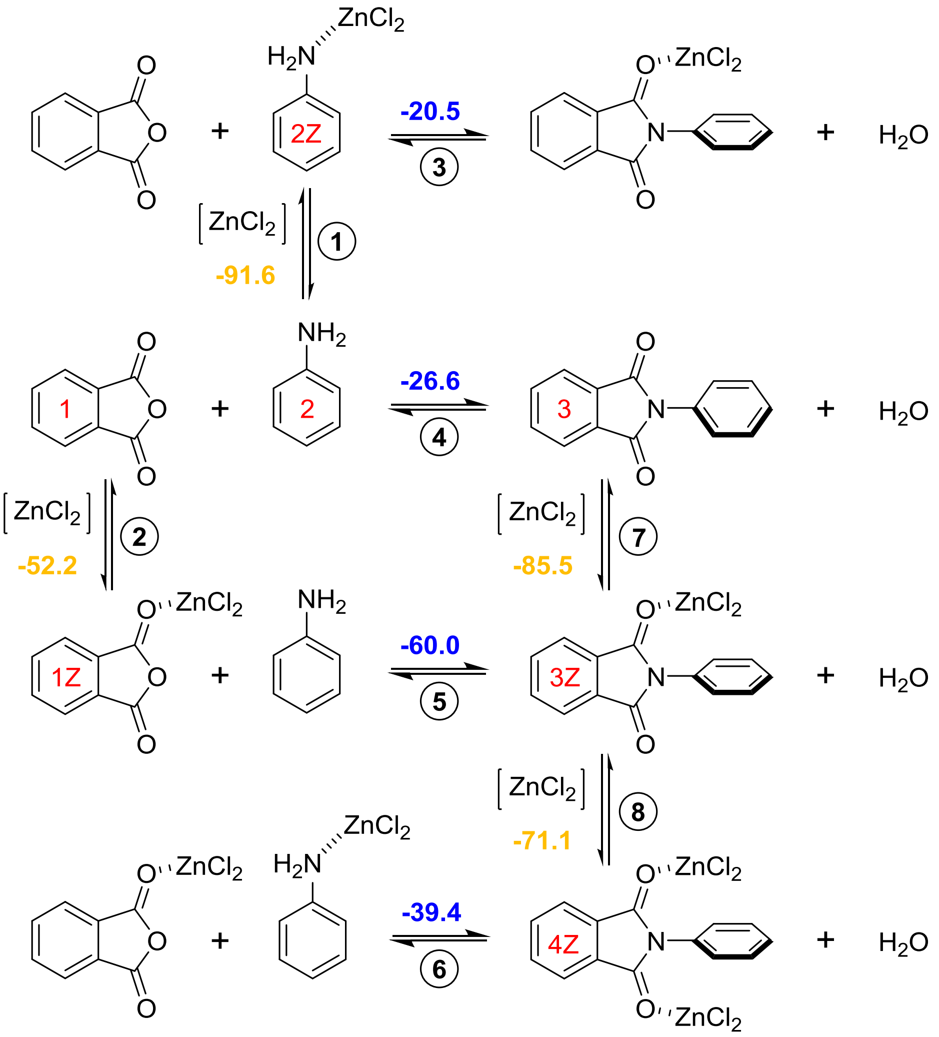

Figure S28. Summary of three individual polymerization reaction pathways, supplemented with calculated reaction enthalpies as total energy differences of the sum of reactants and products, obtained on PBE0-D3/def2-TZVP level of theory.

Table 4. Calculated total energies for investigated model compounds, obtained on PBE0-D3/def2-TZVP level of theory.

Compound	Label	Total Energy [H]
Phthalic anhydride	1	-532.572907
Phthalic anhydride · $ZnCl_2$	1Z	-3231.977974
Aniline	2	-287.371607
Aniline · $ZnCl_2$	2Z	-2986.791682
N-Phenylphthalimide	3	-743.577316
N-Phenylphthalimide · $ZnCl_2$	3Z	-3442.995082
N-Phenylphthalimide· 2·$ZnCl_2$	4Z	-6142.407343
Water	H_2O	-76.377335
$ZnCl_2$	Z	-2699.385181

Reactant				Product			Reaction Enthalpy [kJ/mol]
1	+	2Z	=	3Z	+	H_2O	-20.5
1	+	2	=	3	+	H_2O	-26.6
1Z	+	2	=	3Z	+	H_2O	-60.0
1Z	+	2Z	=	4Z	+	H_2O	-39.4
1	+	$ZnCl_2$	=	1Z			-52.2
2	+	$ZnCl_2$	=	2Z			-91.6
3	+	$ZnCl_2$	=	3Z			-85.5
3Z	+	$ZnCl_2$	=	4Z			-71.1

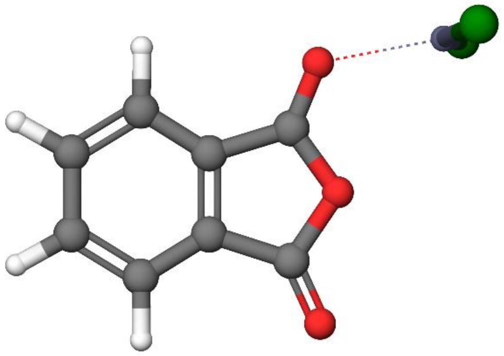

Figure 29. Optimized structure for the 1Z model system, obtained on PBE0-D3/def2-TZVP level of theory.

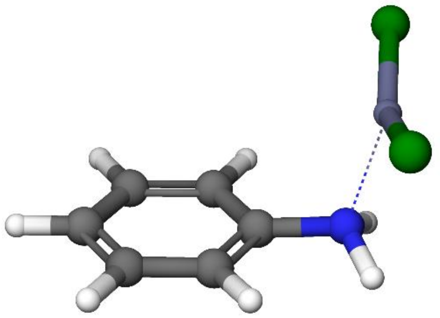

Figure 30. Optimized structure for the 2Z model system, obtained on PBE0-D3/def2-TZVP level of theory.

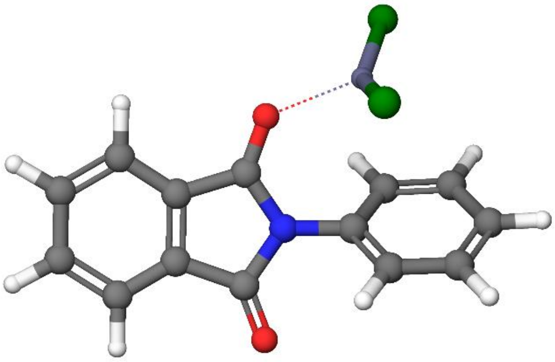

Figure 31. Optimized structure for the 3Z model system, obtained on PBE0-D3/def2-TZVP level of theory.

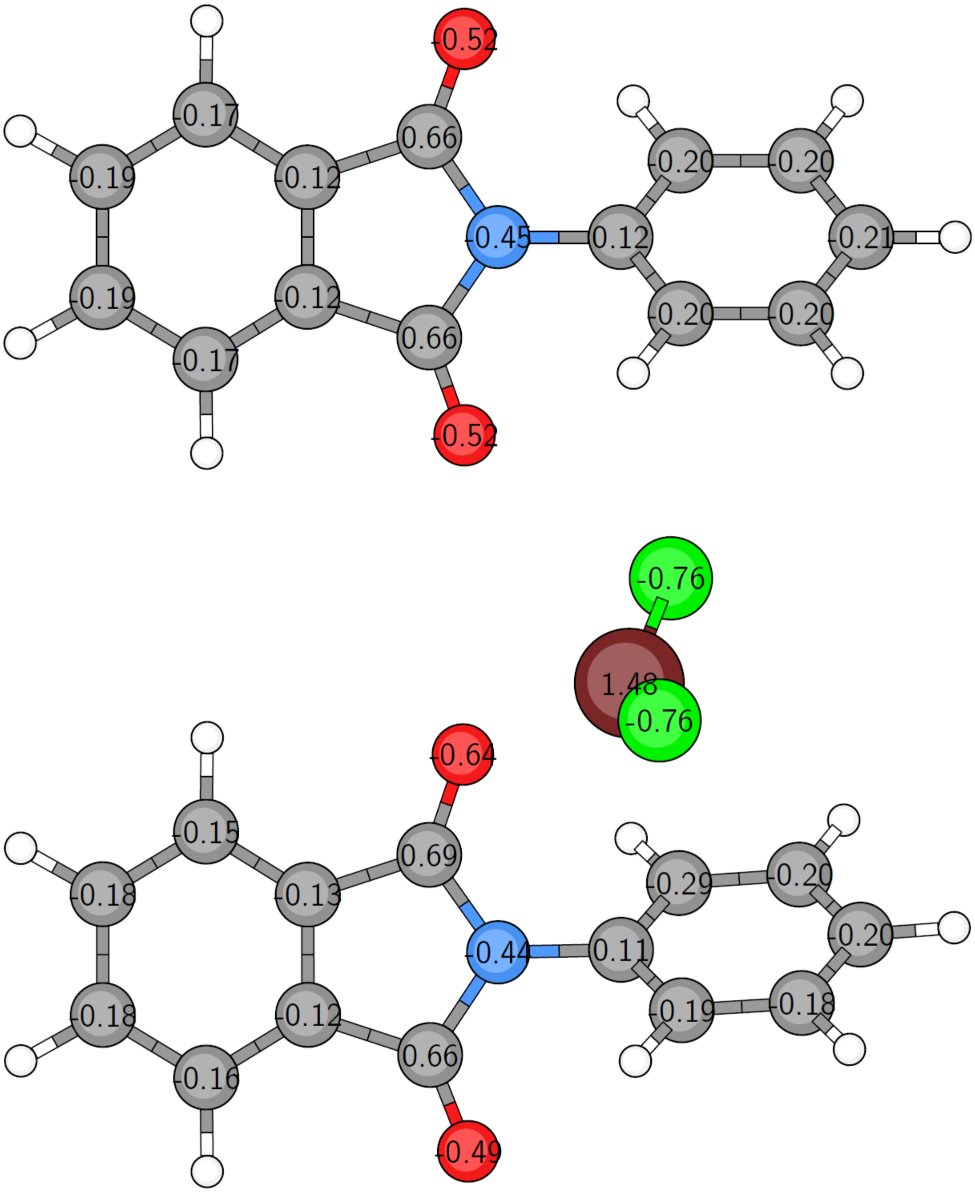

Figure 32. Comparison of partial charges, extracted from the summary of a Natural Population Analysis for the the **3** (top) and the **3Z** (bottom) model system.

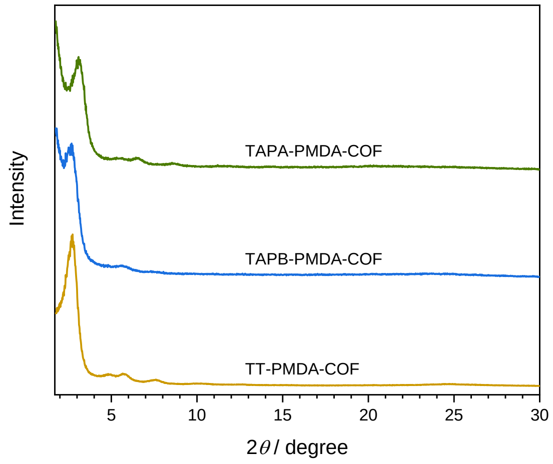

Figure S33. XRPD patterns of TAPA-PMDA-COF (green), TAPB-PMDA-COF (blue) and TT-PMDA-COF (yellow) synthesized according solvothermal literature procedure in NMP/Mesithylene/Isoquinoline.

Table S5. List of data obtained from peak fittings using a Voigt function and the resulting calculated Scherrer crystallite sizes of the ionothermal TAPB-PTCDA-COF.

**Ionothermal TAPB-PTCDA-COF**				
**Bragg Peak**	**Peak Position (2θ)**	**FWHM**	**Crystallite Size D (nm)**	**D (nm) Average**
100	2.11298	0.60025	132.3718305	**112.29**
200	4.37692	0.86225	92.20141425	

Table S6. List of data obtained from peak fittings using a Voigt function and the resulting calculated Scherrer crystallite sizes of the ionothermal and solvothermal TAPB-PMDA-COF.

**Ionothermal TAPB-PMDA-COF**				
**Bragg Peak**	**Peak Position (2θ)**	**FWHM**	**Crystallite Size D (nm)**	**D (nm) Average**
100	2.77758	0.38474	206.5447495	
110	4.91073	0.33593	236.7031444	
200	5.64845	0.42333	187.8895726	**193.51**
210	7.49409	0.41563	191.5474646	
230	12.37508	0.55154	144.8819576	
001	24.16267	2.34523	34.641411	**34.64**
**Solvothermal TAPB-PMDA-COF**				
**Bragg Peak**	**Peak Position (2θ)**	**FWHM**	**Crystallite Size D (nm)**	**D (nm) Average**
100	2.72016	0.70723	112.3610022	**112.36**

Table S7. List of data obtained from peak fittings using a Voigt function and the resulting calculated Scherrer crystallite sizes of the ionothermal and solvothermal TAPA-PMDA-COF.

Bragg Peak	Peak Position (2*θ*)	FWHM	Crystallite Size D (nm)	D (nm) Average
**Ionothermal TAPA-PMDA-COF**				
100	3.22244	0.48247	164.7234143	
110	5.62072	0.49822	159.6450274	
200	6.56218	0.50782	156.6955284	155.01
210	8.6424	0.5585	142.6484469	
230	14.30553	0.52911	151.3216167	
**Solvothermal TAPA-PMDA-COF**				
**Bragg Peak**	**Peak Position (2*θ*)**	**FWHM**	**Crystallite Size D (nm)**	**D (nm) Average**
100	3.12165	0.79129	100.4336845	
110	-	-	-	
200	-	-	-	121.77
210	8.63151	0.55668	143.1137922	
230	-	-	-	

Table S8. List of data obtained from peak fittings using a Voigt function and the resulting calculated Scherrer crystallite sizes of the ionothermal and solvothermal TT-PMDA-COF.

Bragg Peak	Peak Position (2*θ*)	FWHM	Crystallite Size D (nm)	D (nm) Average
**Ionothermal TT-PMDA-COF**				
100	2.67318	0.74271	106.9923722	
110	4.92101	0.74721	106.4171966	116.76
210	7.44826	0.80717	98.62952516	
230	12.48957	0.51565	154.9828203	
001	24.47828	2.95726	27.48838668	27.49
**Solvothermal TT-PMDA-COF**				
**Bragg Peak**	**Peak Position (2*θ*)**	**FWHM**	**Crystallite Size D (nm)**	**D (nm) Average**
100	2.66297	0.83045	95.68804849	
110	4.8814	0.84909	93.64710123	101.65
210	7.57262	0.68849	115.6392463	
230	-	-	-	
001	25.07428	3.51016	23.1850474	23.19

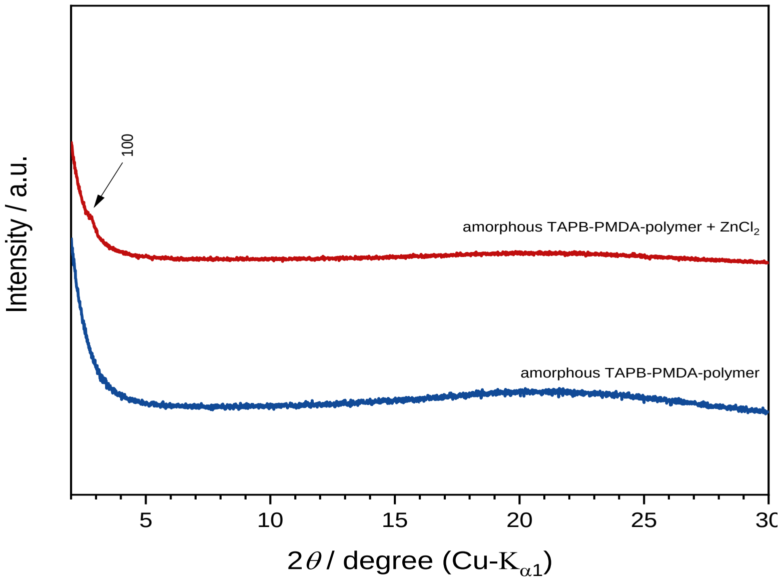

Figure S34. XRPD pattern of the amorphous TAPB-PMDA-polymer (blue) and the same polymer mixed with $ZnCl_2$ and heated to 300 °C for 48 h (red), showing a slight increase in structural orientation by the appearance of the (100) reflection of TAPB-PMDA-COF**.**

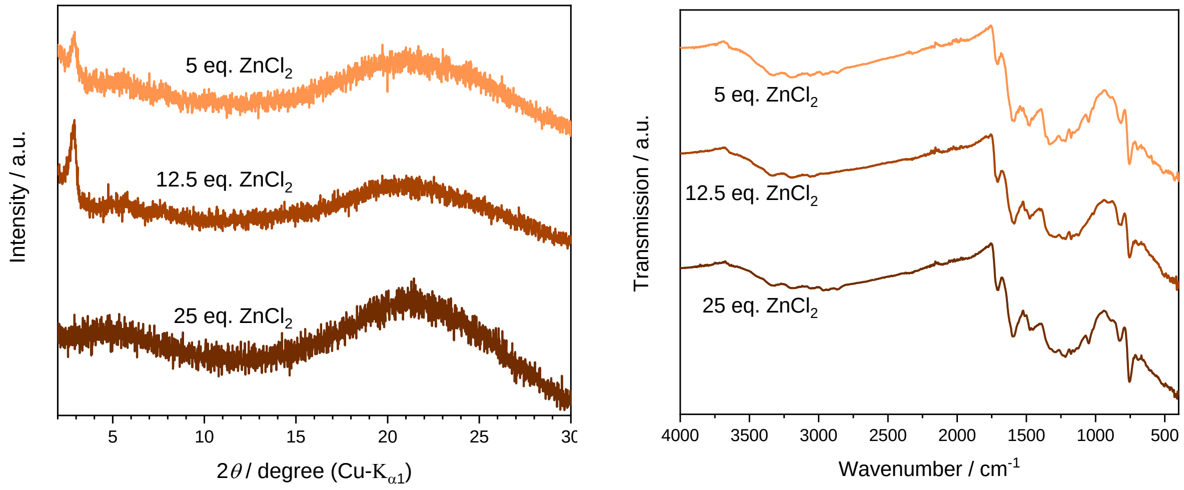

Figure S35. XRPD patterns (λ = Cu-K$_{\alpha1}$) (left) and IR spectra (right) of screening experiments on **TT-PMDA-COF** in $ZnCl_2$, in which the zinc chloride ratio was varied between 5 and 25 equivalents with respect to PMDA, with reaction time and temperature kept constant at 20 h and 300 °C, respectively. XRPD reveals that no product with satisfactory crystallinity is formed. The IR spectra show that although in each case imide condensation took place, a significant broadening of the vibrational bands is observed, which indicates the loss of short-range order and therefore points towards partial decomposition of the linker molecules - most probably the amine linker is affected, since PMDA is stable under these conditions (see TAPB-PMDA-COF).

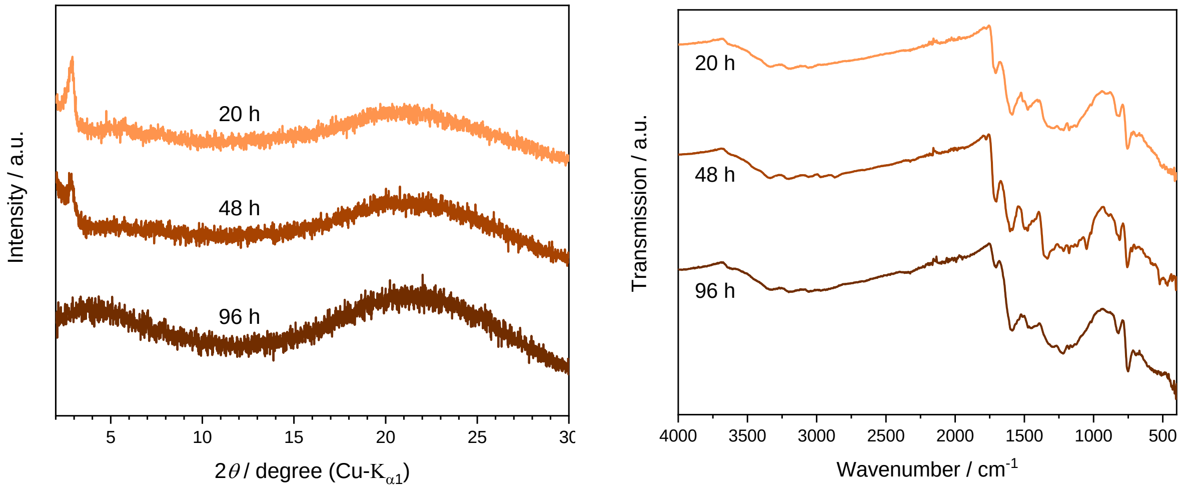

Figure S36. XRPD patterns (λ = Cu-$K_{\alpha1}$) (left) and IR spectra (right) of screening experiments on **TT-PMDA-COF** in $ZnCl_2$, in which the reaction time was varied between 20 and 96 h with the zinc chloride portion and reaction temperature kept constant at 12.5 eq. w.r.t. PMDA and 300 °C, respectively. XRPD reveals that no product with satisfactory crystallinity is formed. The IR spectra again show a significant broadening of the vibrational bands, which indicates the loss of short-range order and therefore points towards partial decomposition of the amine linker.

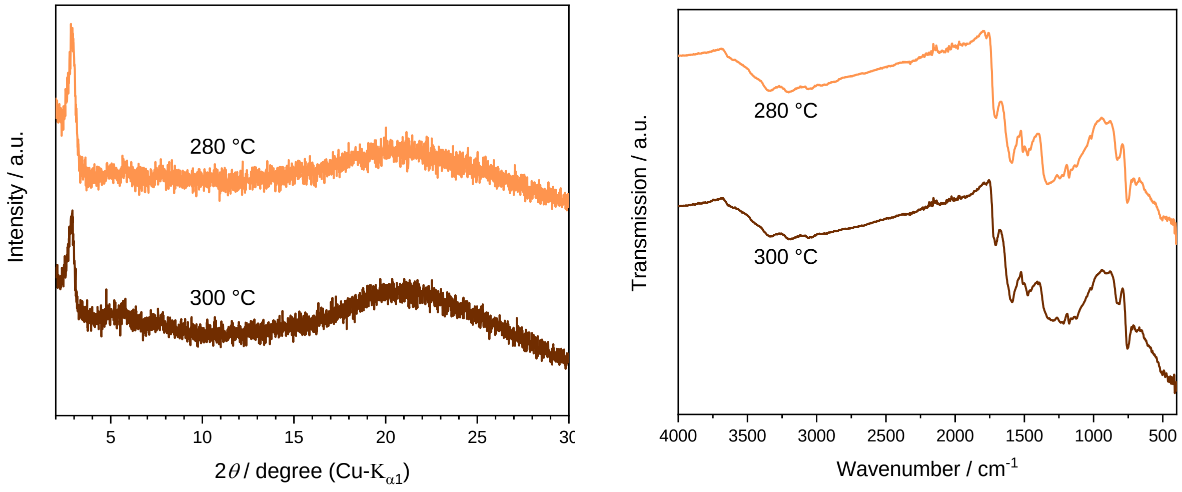

Figure S37. XRPD patterns (λ = Cu-$K_{\alpha1}$) (left) and IR spectra (right) of screening experiments using **TT-PMDA-COF** in $ZnCl_2$, in which the reaction temperature was varied between 280 and 300 °C with the zinc chloride portion and reaction time kept constant at 12.5 eq. w.r.t. PMDA and 48 h, respectively. XRPD reveals that in neither case a product with satisfactory crystallinity is formed. The IR spectra again show a significant broadening of the vibrational bands, which indicates the loss of short-range order and therefore points towards partial decomposition of the amine linker.

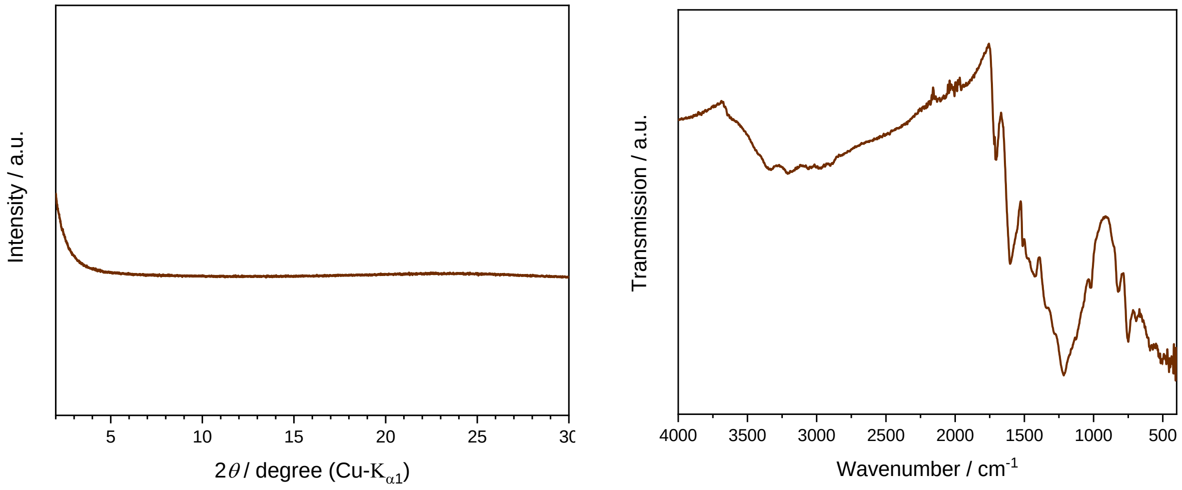

Figure S38. XRPD pattern (λ = Cu-$K_{\alpha1}$) (left) and IR spectrum (right) of **TAPA-PMDA-COF** synthesized in $ZnCl_2$ at 290 °C for 30 h. XRPD reveals that no crystalline product is formed. Although the imide-vibration can be identified at 1723 cm^{-1} the IR spectrum shows a significant broadening of the vibrational bands, which indicates the loss of short range order and therefore points towards partial decomposition of the amine linker.

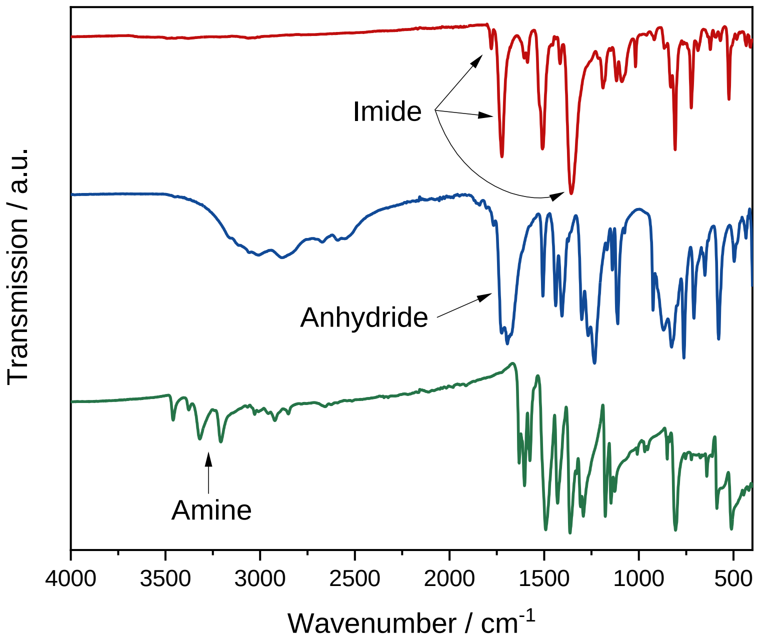

Figure S39. FT-IR spectra of **TT-PMDA-COF** (red) and its precursor molecules PMDA (blue) and TT (green). The absence of amine or anhydride vibrational bands in the COF indicate complete imide formation.

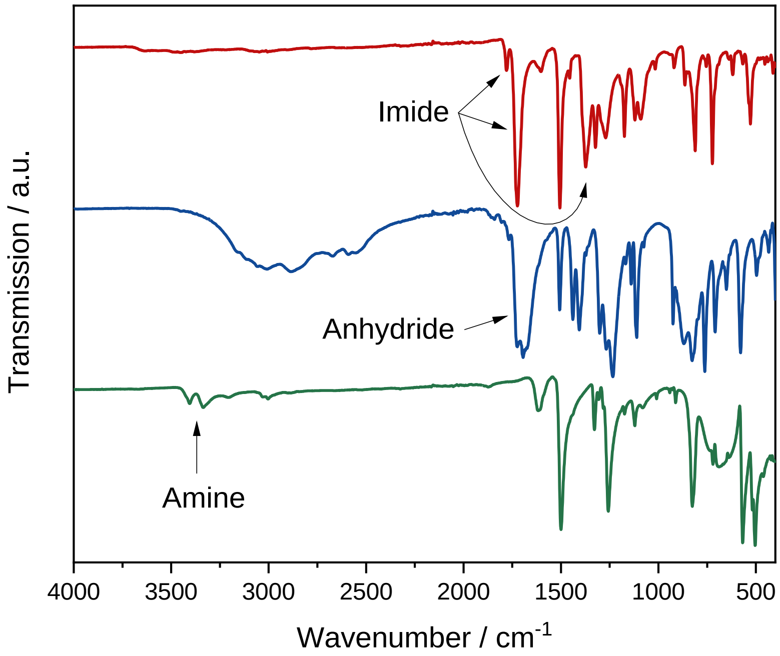

Figure S40. FT-IR spectra of **TAPA-PMDA-COF** (red) and its precursor molecules PMDA (blue) and TAPA (green). The absence of amine or anhydride vibrational bands in the COF indicate complete imide formation.

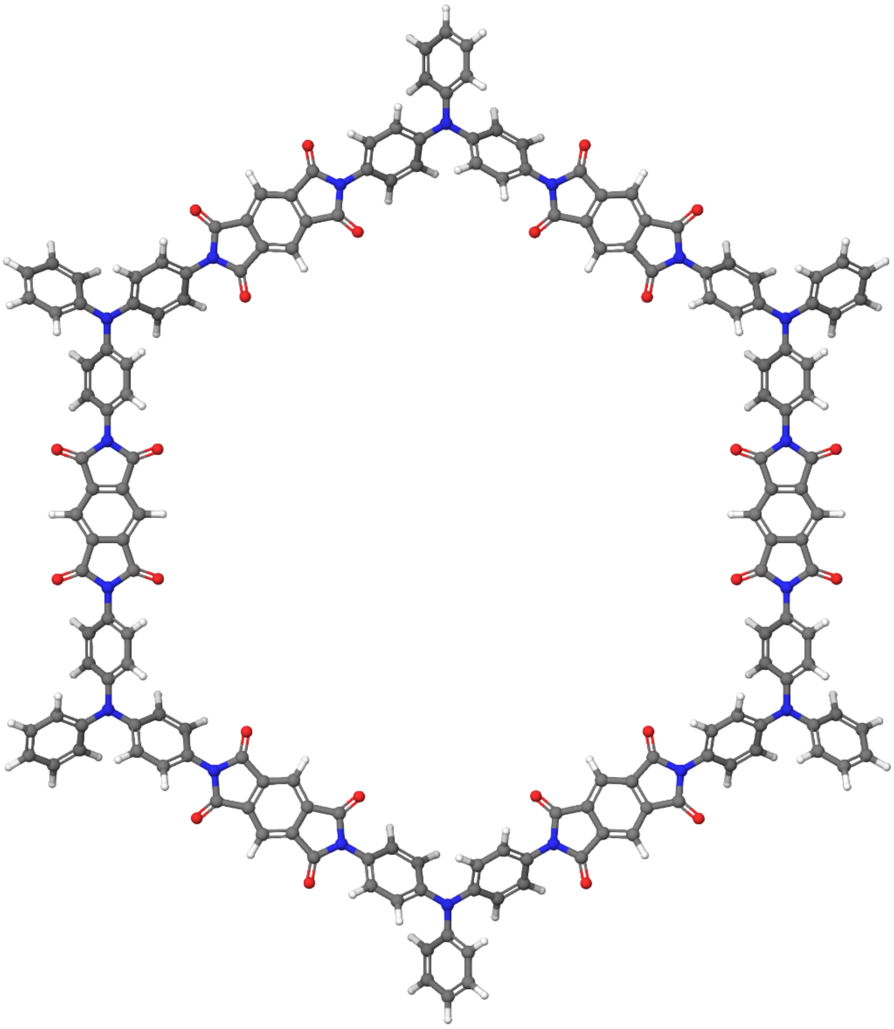

Figure S41. Structure of a single pore of the **TAPA-PMDA** COF, obtained by composing a supercell from a 2D periodic geometry optimization of the corresponding asymmetric unit on RI-PBE-D3/def2-TZVP level of theory.

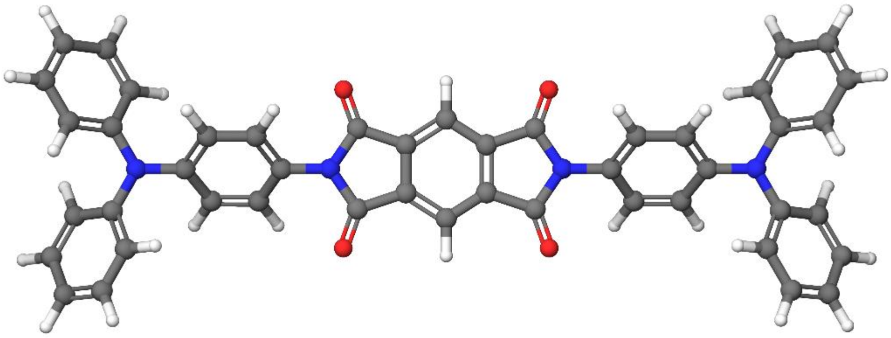

Figure S42. Structure of the **TAPA-PMDA** NMR model, modeled by cutting the supercell obtained from the 2D optimized structure of the TAPA-PMDA COF model.

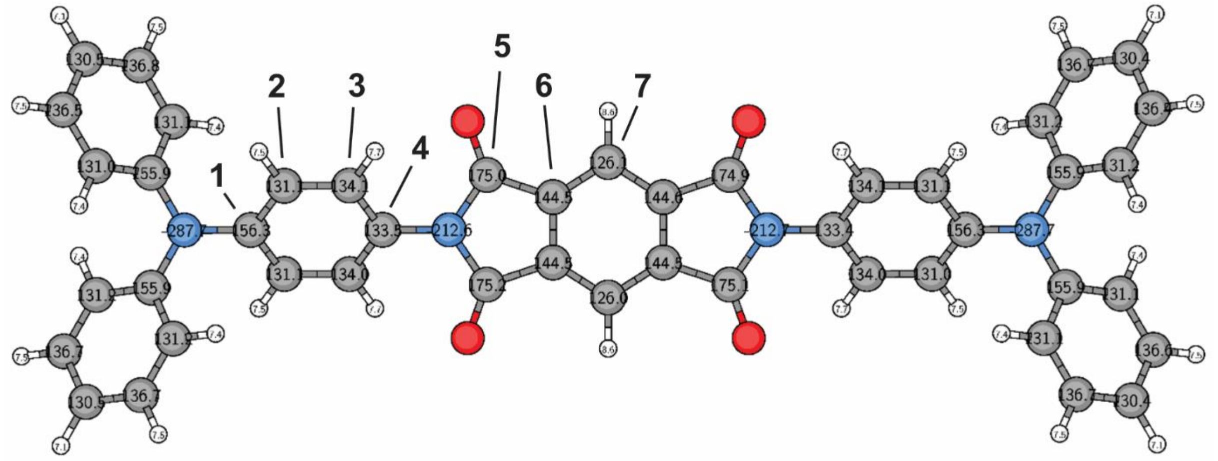

Figure S43. Calculated NMR chemical shifts for the **TAPA-PMDA** NMR model, obtained on B97-2/pcsSeg-2 level of theory.

Table S9. Calculated NMR chemical shifts for the **TAPA-PMDA** NMR model, obtained on B97-2/pcsSeg-2 level of theory.

Atom Label	Atom	NMR Chemical Shift [ppm]
1	C	156.3
2	C	131.1
3	C	134.1
4	C	133.5
5	C	175.0
6	C	144.5
7	C	126.1

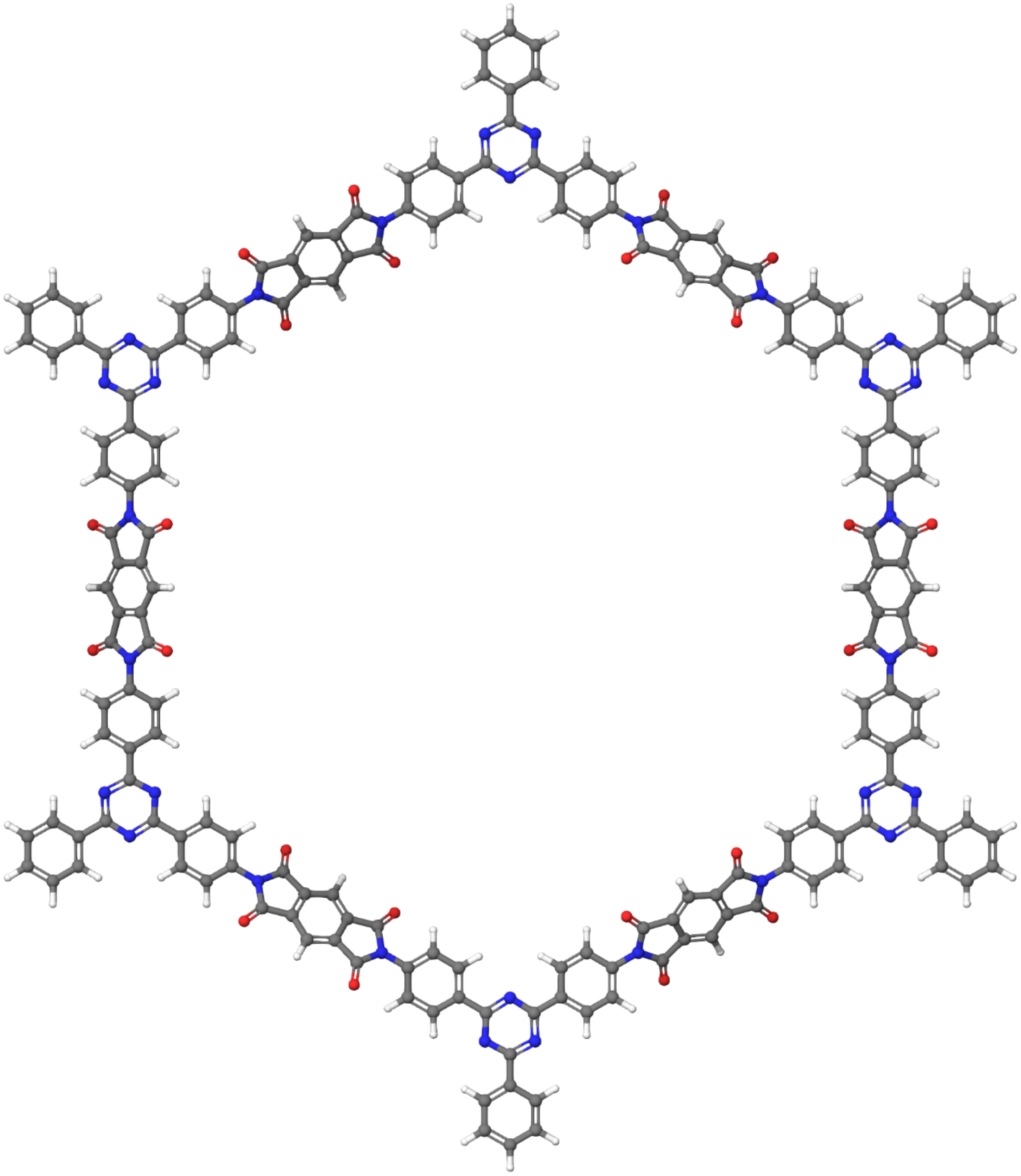

Figure S44. Structure of a single pore of the **TT-PMDA** COF, obtained by composing a supercell from a 2D periodic geometry optimization of the corresponding asymmetric unit on RI-PBE-D3/def2-TZVP level of theory.

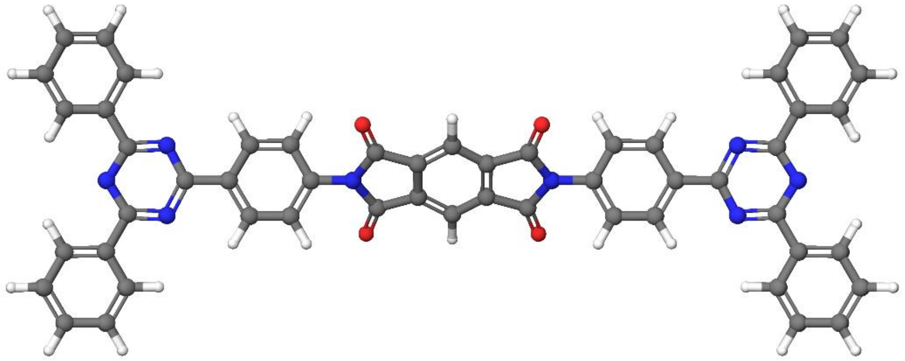

Figure S45. Structure of the **TT-PMDA** NMR model, modeled by cutting the supercell obtained from the 2D optimized structure of the TAPA-PMDA COF model.

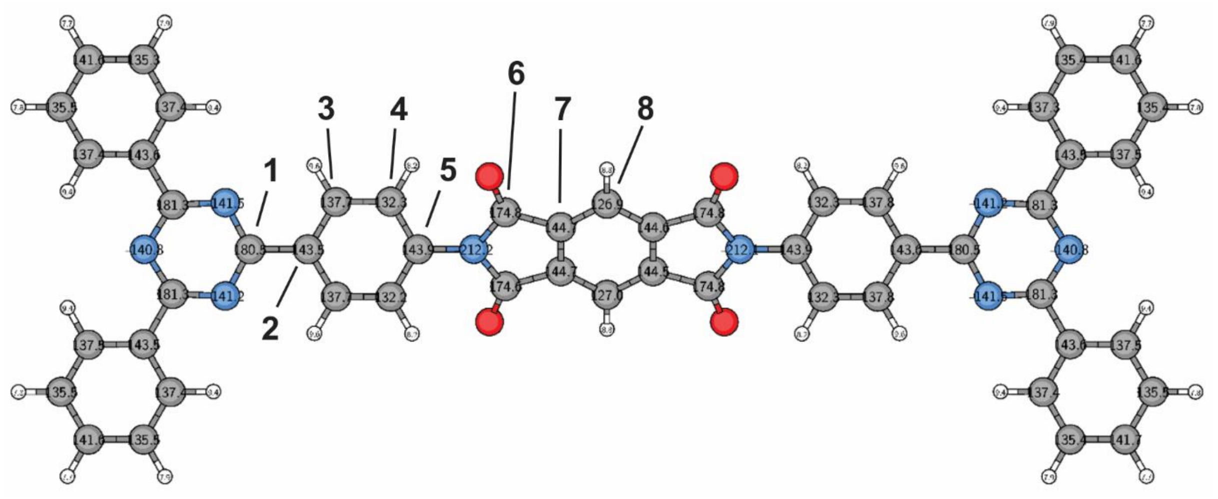

Figure S46. Calculated NMR chemical shifts for the **TT-PMDA** NMR model, obtained on B97-2/pcsSeg-2 level of theory.

Table S10. Calculated NMR chemical shifts for the **TT-PMDA** NMR model, obtained on B97-2/pcsSeg-2 level of theory.

Atom Label	Atom	NMR Chemical Shift [ppm]
1	C	180.5
2	C	143.5
3	C	137.7
4	C	132.3
5	C	143.9
6	C	174.8
7	C	144.7
8	C	126.9

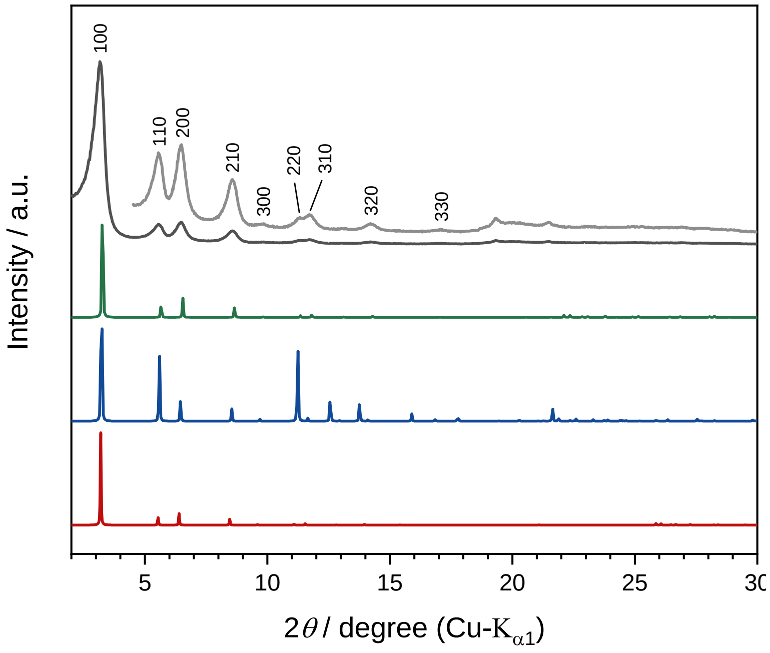

Figure S47. Experimental XRPD pattern (λ = Cu-K$_{\alpha 1}$) of **TAPA-PMDA-COF** (black) compared to simulated patterns of the COF assuming $P\bar{3}1m$ symmetry (green), $P\bar{3}1c$ symmetry (blue) and *Cmcm* symmetry (red).

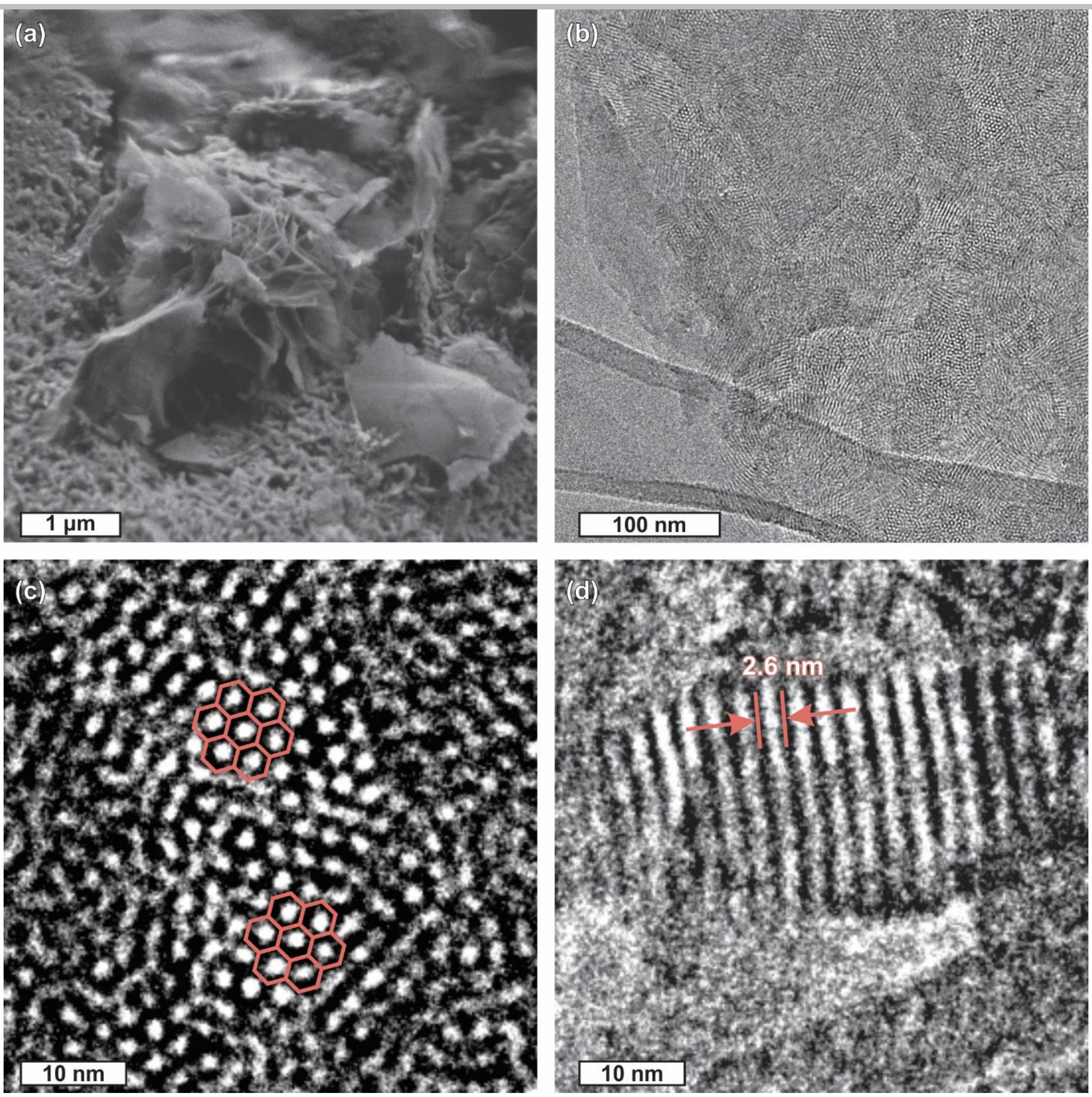

Figure S48. SEM image (a) and TEM images (b) – (d) of **TAPA-PMDA-COF**. (c) shows the hexagonal pores highlighted in red and (d) shows straight channels with a diameter of ~ 2.6 nm.

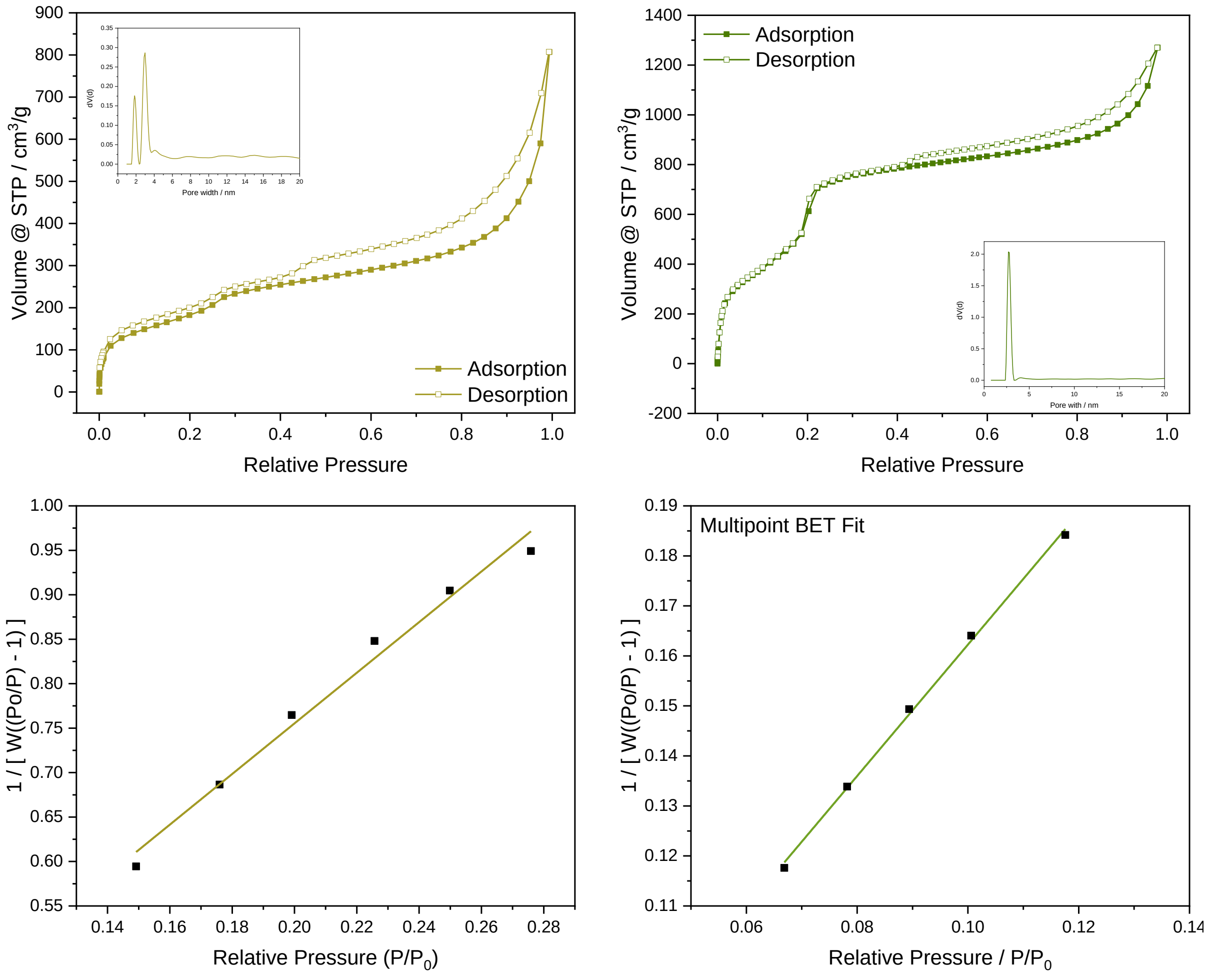

Figure S49. Top: Argon gas adsorption and desorption isotherms for **TT-PMDA** (yellow) and **TAPA-PMDA** (green). Insets: Respective calculated pore size distributions of PI-COFs from fitting the quenched solid-state density functional theory (QSDFT) model. Bottom: Multipoint BET fits of TT-PMDA (yellow) and TAPA-PMDA (green).

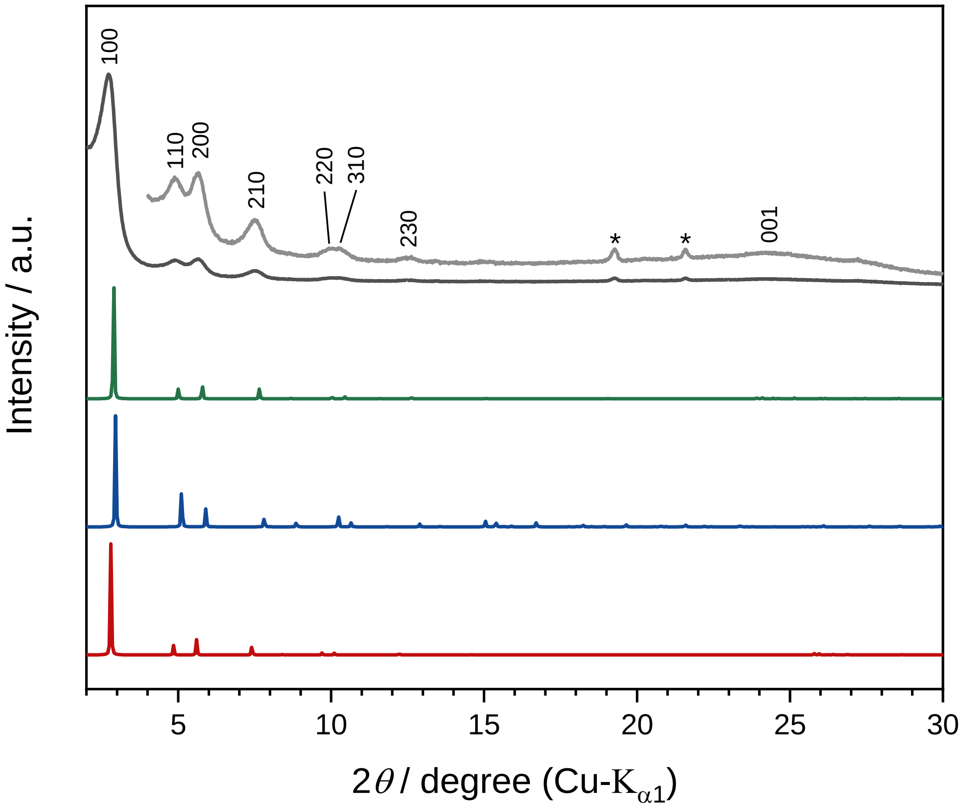

Figure S50. Experimental XRPD pattern (λ = Cu-$K_{α1}$) of **TT-PMDA-COF** (black) compared to simulated patterns of the COF assuming $P\bar{3}1m$ symmetry (green), $P\bar{3}1c$ symmetry (blue) and *Cmcm* symmetry (red). Reflections marked with an asterisk show minor impurities, which couldn't be eliminated.

Table S11. Rietveld refinement of **TAPA-PMDA-** and **TT-PMDA-COF** at room temperature.

Fitted Pattern	TAPA-PMDA-COF	TT-PMDA-COF
**Space group**	$P\bar{3}1m$	$P\bar{3}1m$
**Constraints**	*c* = fixed	-
**Rwp (%)**	9.031	2.220
**Cell Volume (Å³)**	3379(6)	3971(7)
**A (Å)**	31.1(6)	35.2(5)
**B (Å)**	31.1(6)	35.2(5)
**C (Å)**	4.018	3.7(2)
**α (°)**	90	90
**β (°)**	90	90
**γ (°)**	120	120

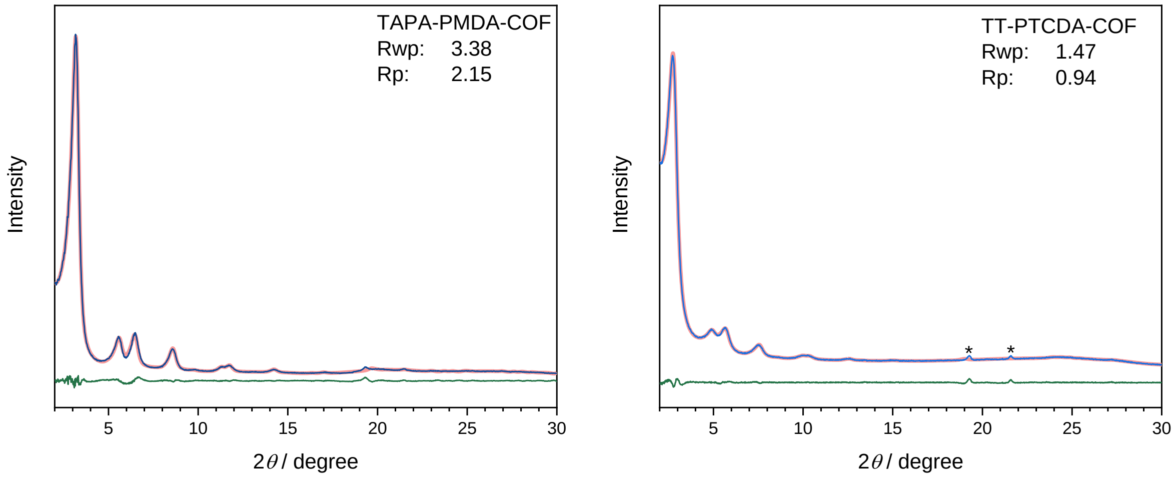

Figure S51. Pawley refinement of TAPA-PMDA-COF (left) and TT-PMDA-COF (right) with fixed lattice sizes and fit parameters obtained from Rietveld refinement. Reflections marked with an asterisk show minor impurities, which couldn't be eliminated.

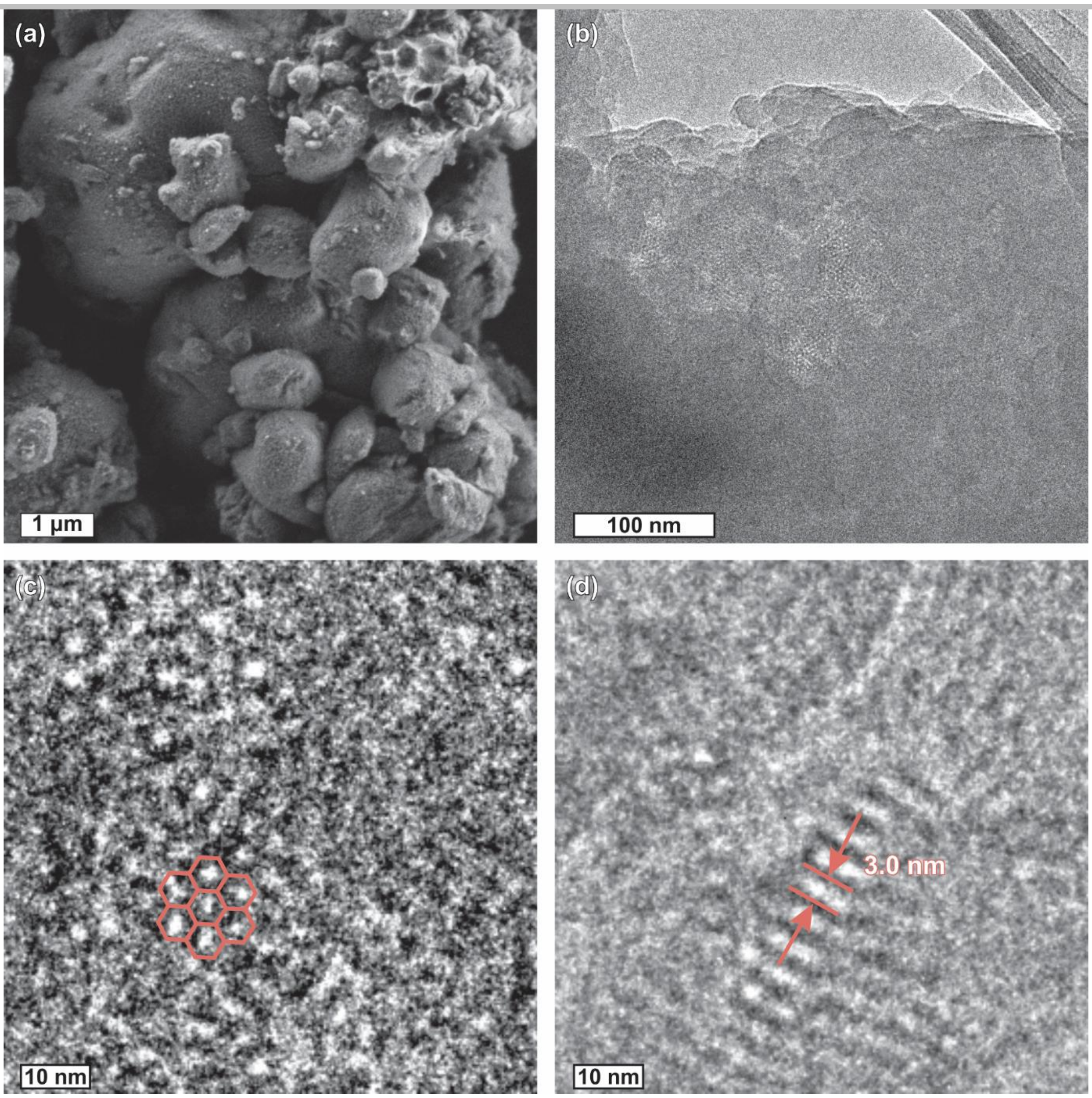

Figure S52. SEM image (a) and TEM images (b) – (e) of **TT-PMDA-COF**. (c) shows the hexagonal pores highlighted in red and (d) shows straight channels with a diameter of ~ 3.0 nm and (e) shows straight channels with a diameter of ~ 1.7 nm.

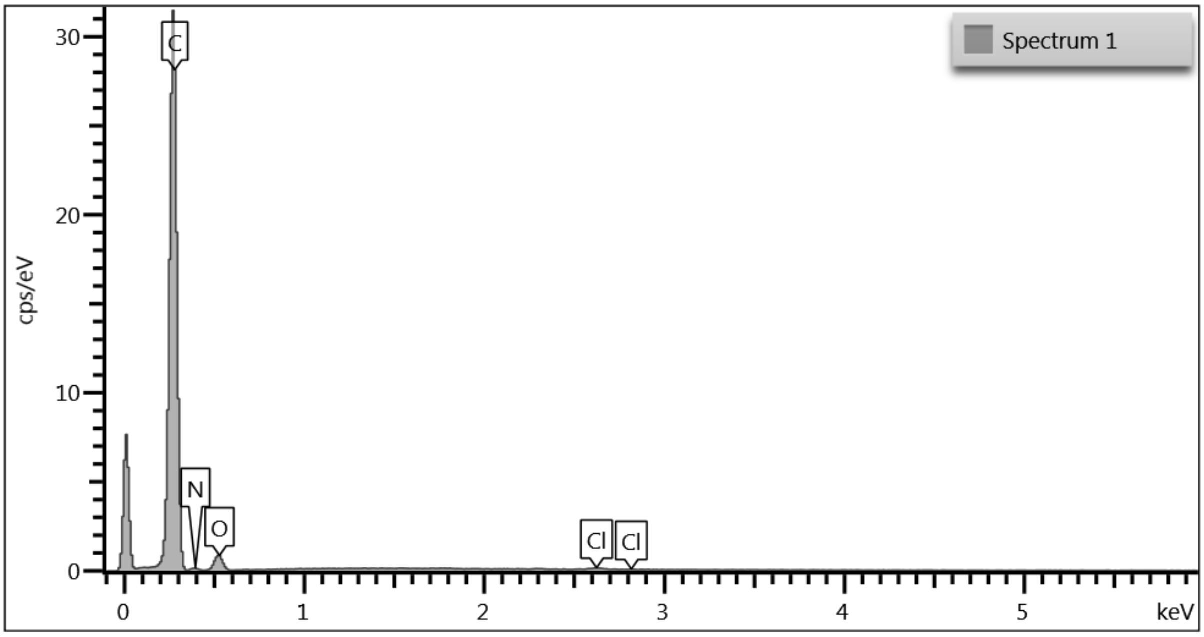

Figure S53. EDX spectrum of **TAPB-PTCDA-COF** showing only traces of chloride residuals in the COF.

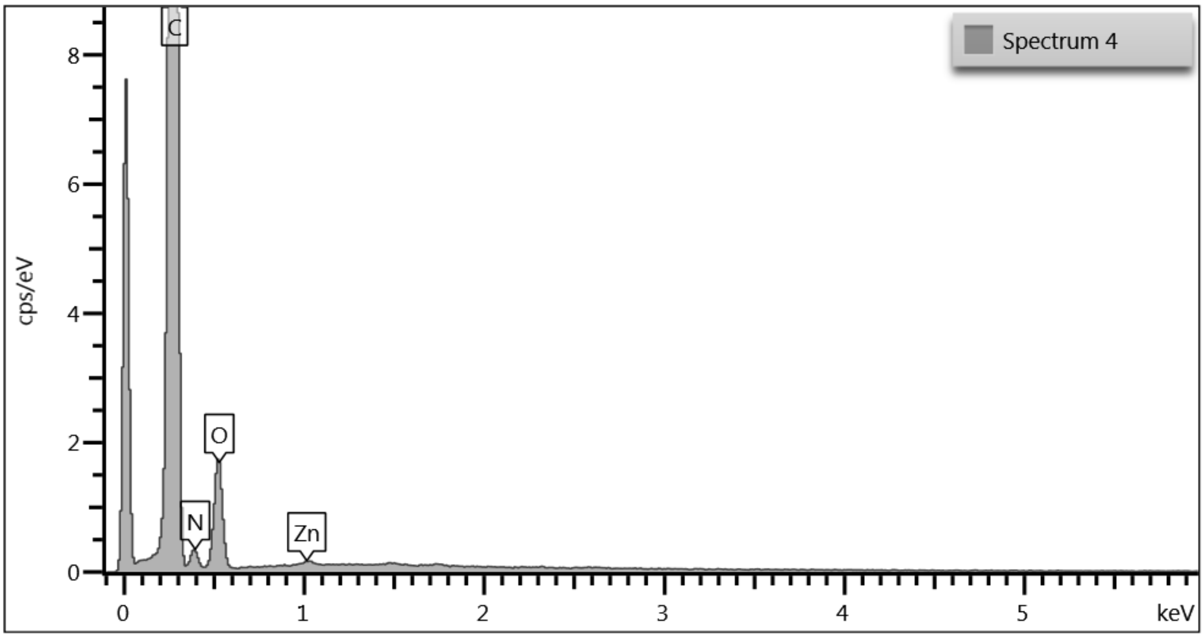

Figure S54. EDX spectrum of **TAPB-PMDA-COF** showing trace amounts of zinc residuals in the COF.

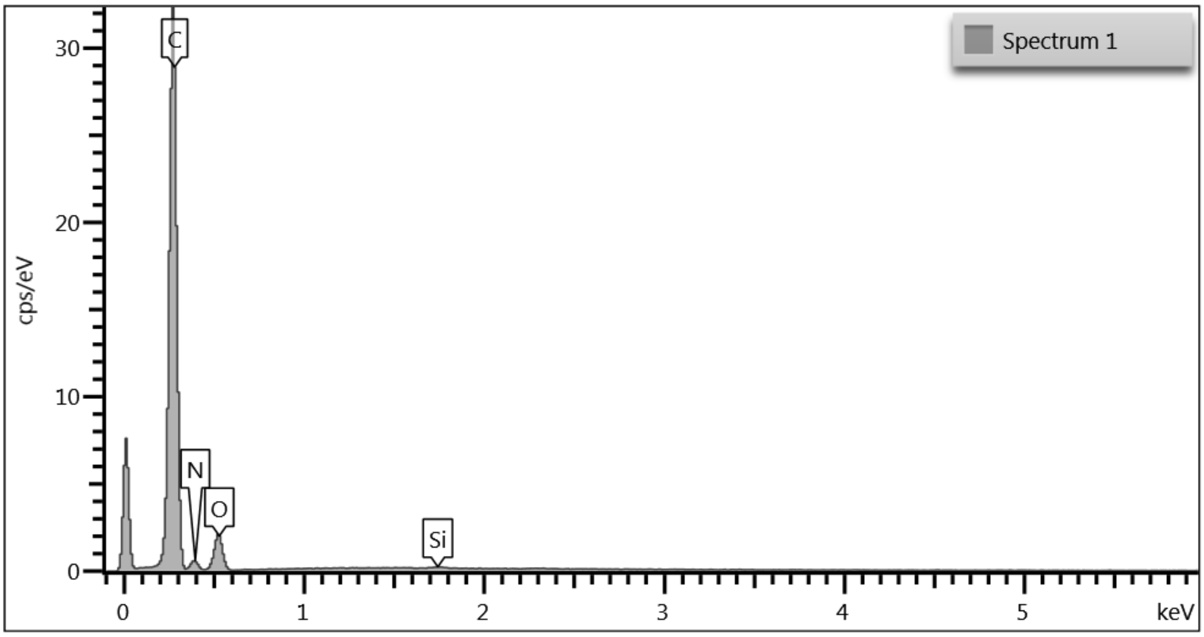

Figure S55. EDX spectrum of **TT-PMDA-COF** showing the absence of $ZnCl_2$ residuals in the COF.

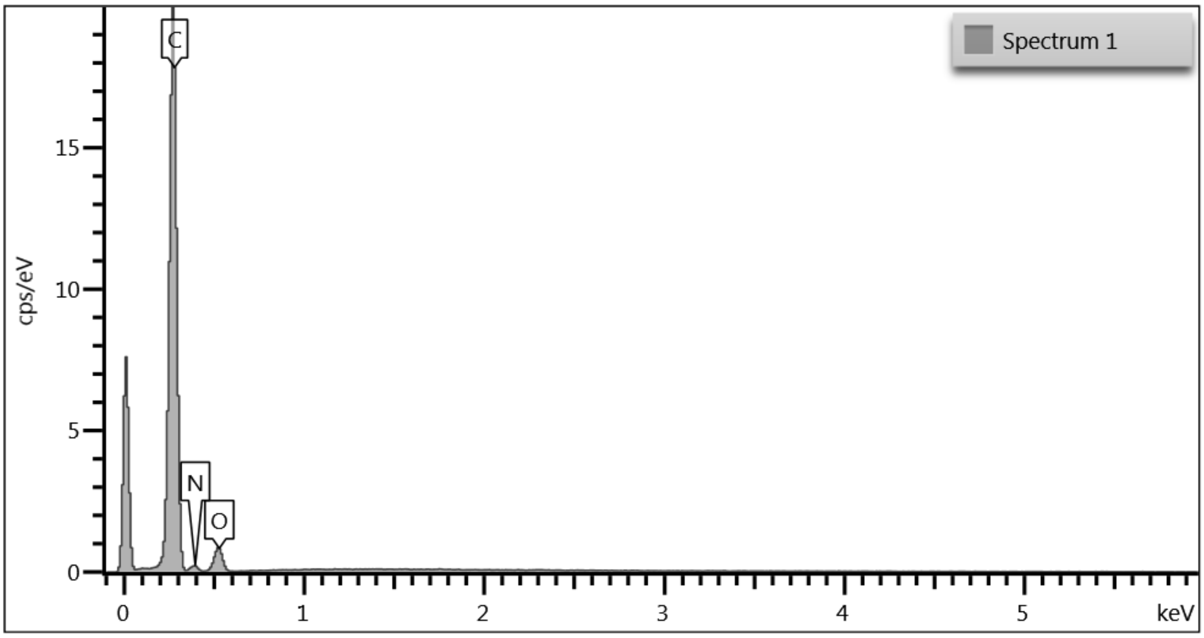

Figure S56. EDX spectrum of **TAPA-PMDA-COF** showing the absence of $ZnCl_2$ residuals in the COF.

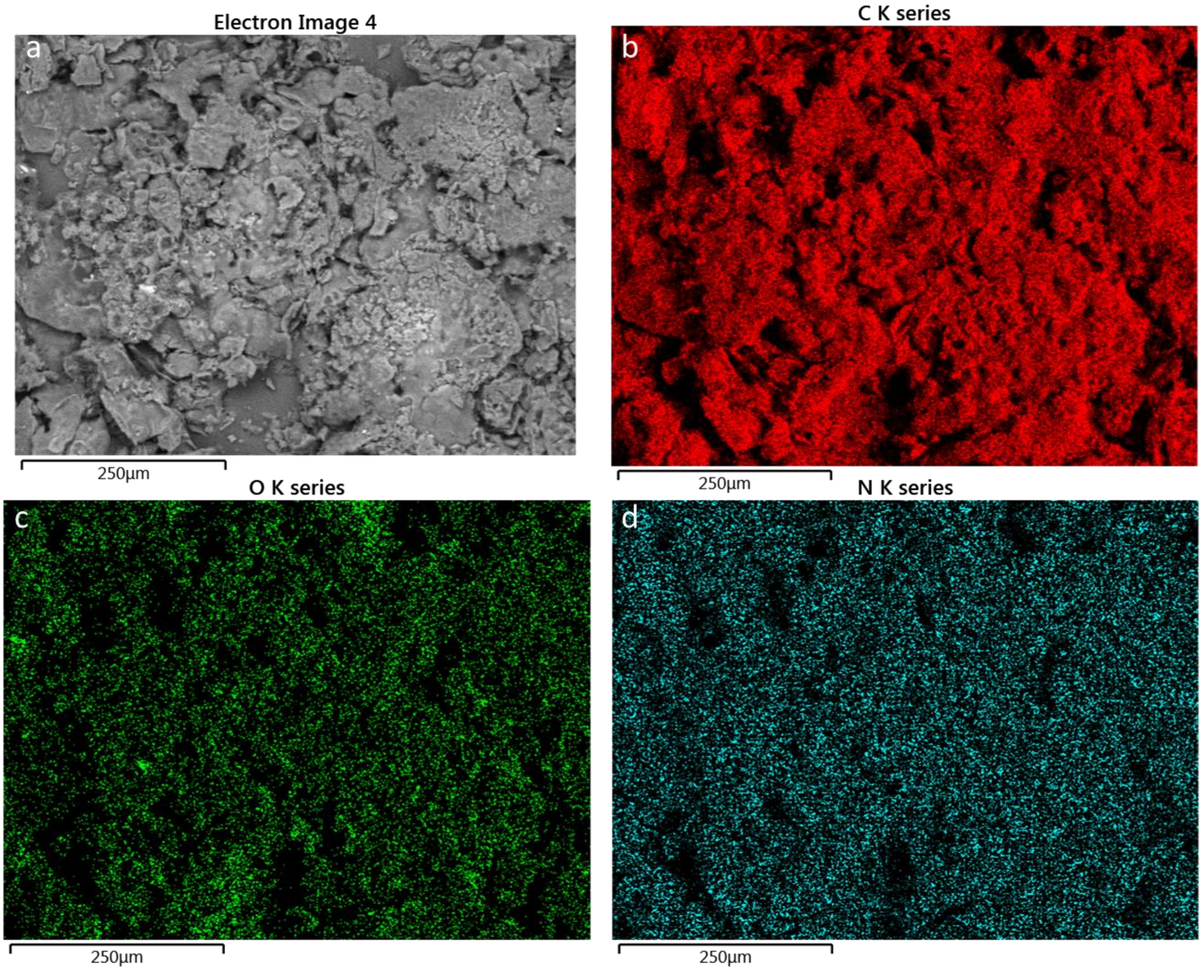

Figure S57. SEM image (a) and elemental mapping of carbon (b), oxygen (c) and nitrogen (d) of **TAPB-PTCDA-COF** showing a homogeneous distribution of the corresponding elements.

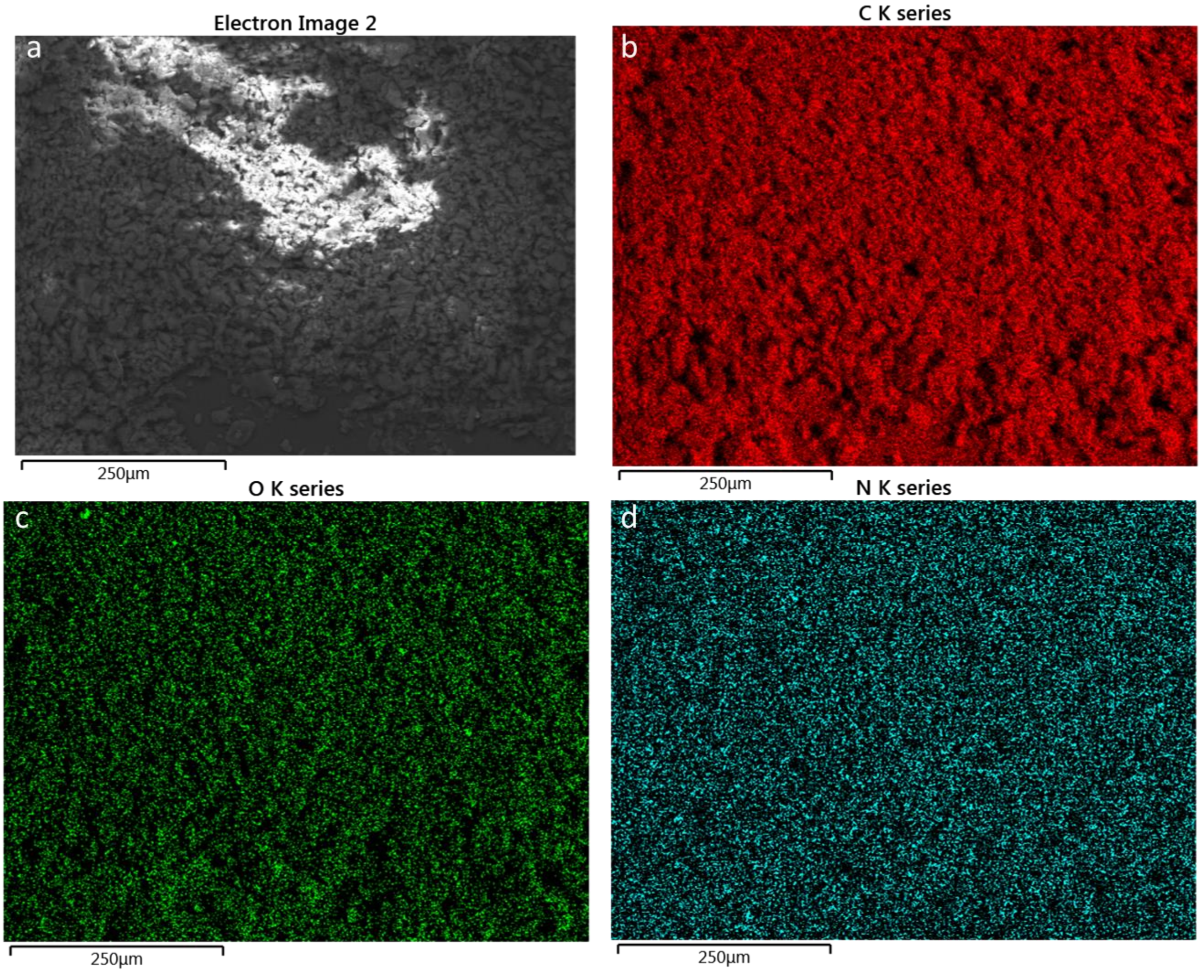

Figure S58. SEM image (a) and elemental mapping of carbon (b), oxygen (c) and nitrogen (d) of **TAPB-PMDA-COF** showing a homogeneous distribution of the corresponding elements.

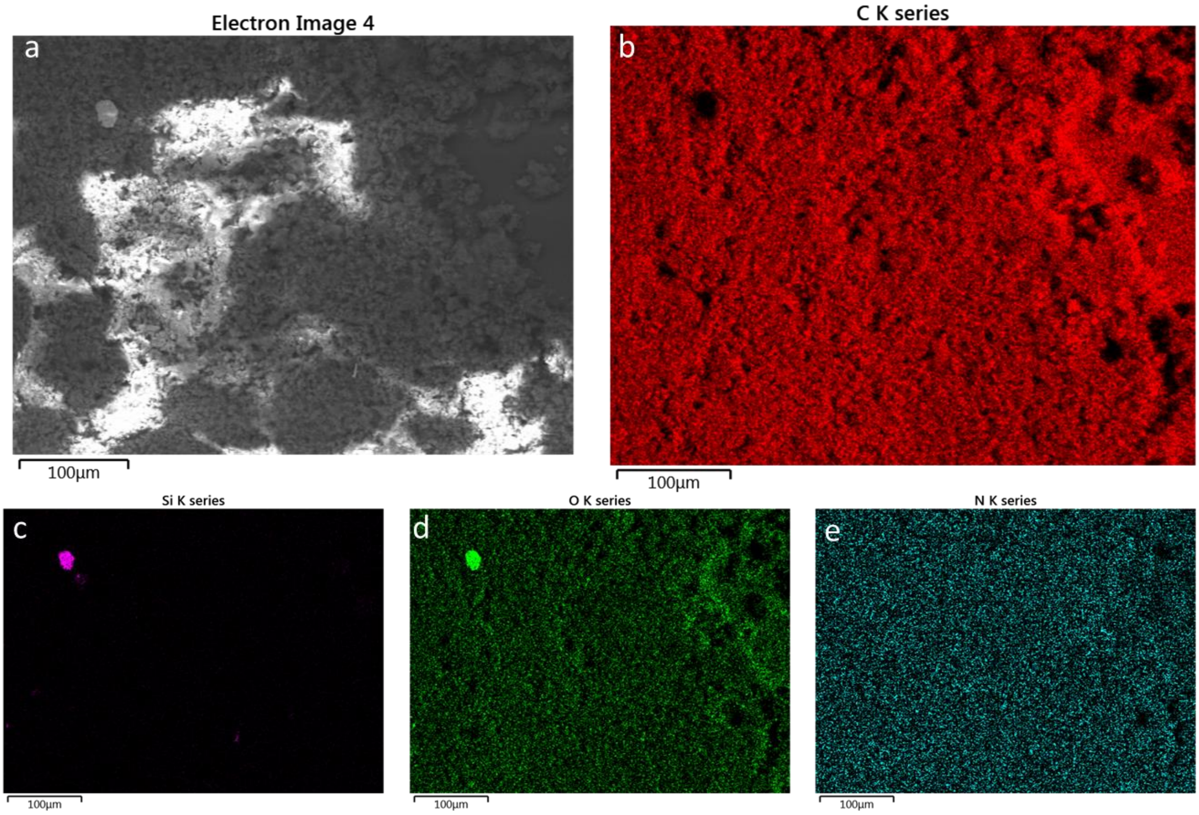

Figure S59. SEM image (a) and elemental mapping of carbon (b), silicon (c), oxygen (d) and nitrogen (e) of **TT-PMDA-COF** showing a homogeneous distribution of the corresponding elements. The silicon found in this sample corresponds to a quartz impurity.

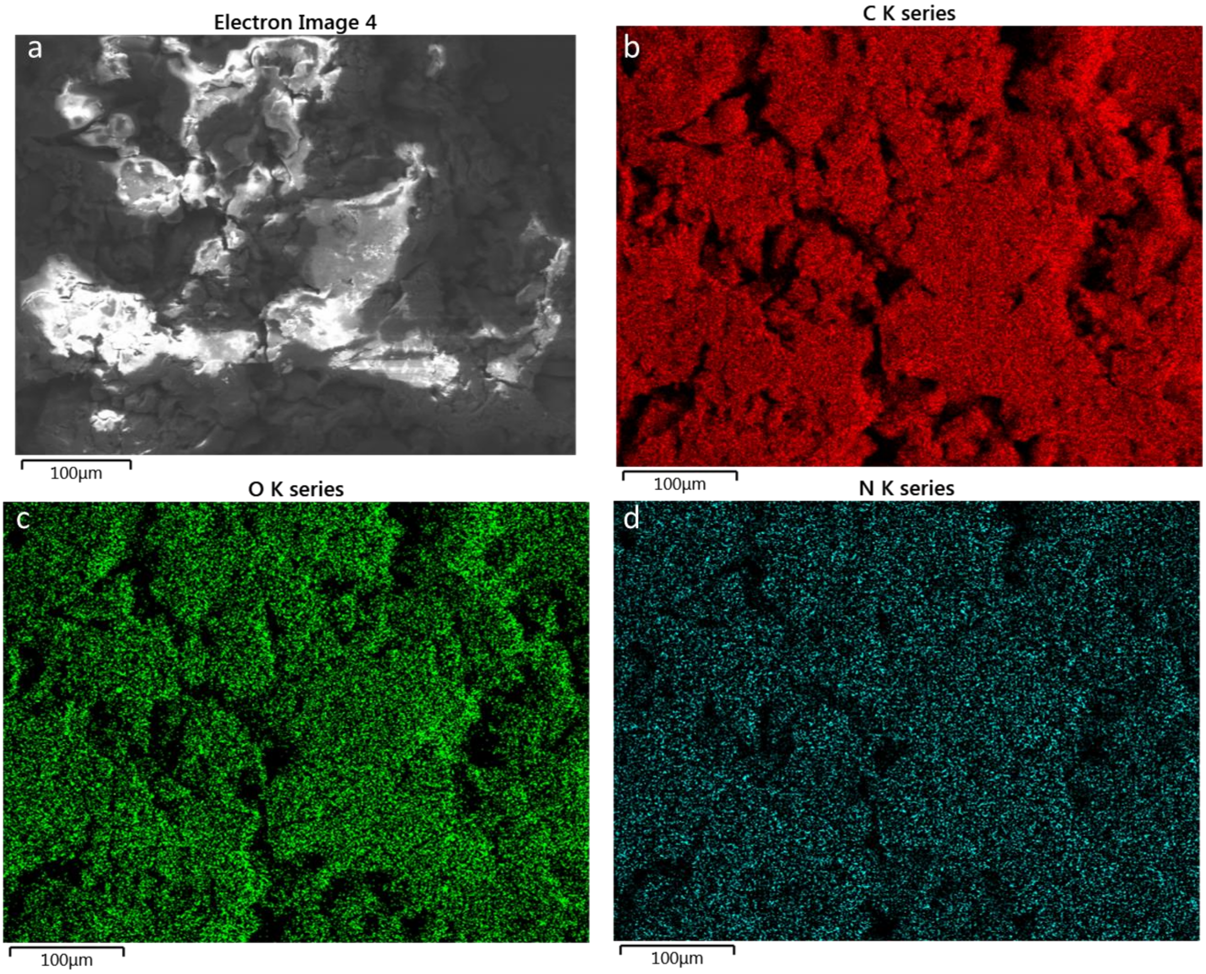

Figure S60. SEM image (a) and elemental mapping of carbon (b), silicon (c), oxygen (d) and nitrogen (e) of **TAPA-PMDA-COF** showing a homogeneous distribution of the corresponding elements.

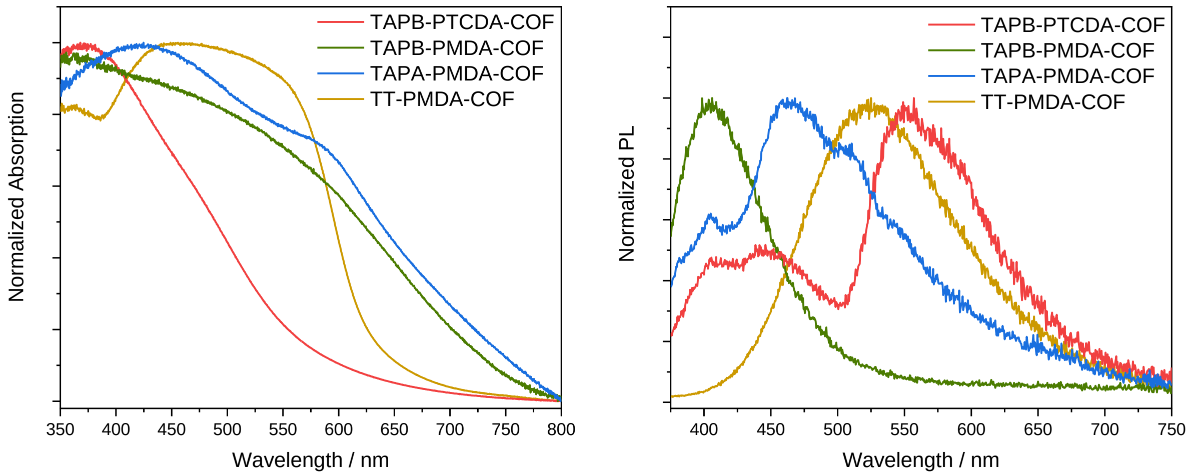

Figure S61. Diffuse reflectance spectra (left) and photoluminescence spectra (right) of the ionothermal imide-linked COFs TAPB-PTCDA (red), TAPB-PMDA (green), TAPA-PMDA (blue) and TT-PMDA (yellow).

References

[1] H. M. Rietveld, *J. Appl. Crystallogr.* **1969**, *2*, 65-71.
[2] B. Optics, *Chem. Eng. News* **2014**, 16-16.
[3] A. A. Coelho, *J. Appl. Crystallogr.* **2000**, *33*, 899-908.
[4] a) A. Schafer, C. Huber, R. Ahlrichs, *J. Chem. Phys.* **1994**, *100*, 5829-5835; b) J. P. Perdew, K. Burke, M. Ernzerhof, *Phys. Rev. Lett.* **1996**, *77*, 3865-3868; c) K. Eichkorn, F. Weigend, O. Treutler, R. Ahlrichs, *Theor. Chem. Acc.* **1997**, *97*, 119-124; d) S. Grimme, J. Antony, S. Ehrlich, H. Krieg, *J. Chem. Phys.* **2010**, *132*, 154104.
[5] a) A. M. Burow, M. Sierka, F. Mohamed, *J. Chem. Phys.* **2009**, *131*, 214101; b) L. Grajciar, *J. Comput. Chem.* **2015**, *36*, 1521-1535; c) A. M. Burow, M. Sierka, *J. Chem. Theory Comput.* **2011**, *7*, 3097-3104.
[6] a) R. Lazarski, A. M. Burow, L. Grajciar, M. Sierka, *J. Comput. Chem.* **2016**, *37*, 2518-2526; b) R. Lazarski, A. M. Burow, M. Sierka, *J. Chem. Theory Comput.* **2015**, *11*, 3029-3041.
[7] TURBOMOLE V7.3 2018, a development of University of Karlsruhe and Forschungszentrum Karlsruhe GmbH, 1989-2007, TURBOMOLE GmbH, since 2007; available from http://www.turbomole.com.
[8] a) C. Adamo, V. Barone, *J Chem Phys* **1999**, *110*, 6158-6170; b) M. Ernzerhof, G. E. Scuseria, *J. Chem. Phys.* **1999**, *110*, 5029-5036.
[9] A. E. Reed, R. B. Weinstock, F. Weinhold, *J. Chem. Phys.* **1985**, *83*, 735-746.
[10] a) P. J. Wilson, T. J. Bradley, D. J. Tozer, *J. Chem. Phys.* **2001**, *115*, 9233-9242; b) F. Jensen, *J. Chem. Theory Comput.* **2015**, *11*, 132-138.
[11] a) J. Kussmann, C. Ochsenfeld, *J. Chem. Phys.* **2013**, *138*, 134114; b) J. Kussmann, C. Ochsenfeld, *J. Chem. Theory Comput.* **2015**, *11*, 918-922.
[12] D. Mullangi, S. Shalini, S. Nandi, B. Choksi, R. Vaidhyanathan, *J. Mater. Chem. A* **2017**, *5*, 8376-8384.

Author Contributions

J. Maschita, T. Banerjee and F. Haase conceived the project. J. Maschita carried out the experiments. G. Savasci performed quantum-chemical calculations. T. Banerjee, C. Ochsenfeld and B. V. Lotsch supervised the project. The manuscript was written through contributions of all authors.
